# Exploring Polyene Construction Tactics for the Total Synthesis of Ocular Pyridinium Bisretinoids of Lipofuscin A2E and pdA2E

**DOI:** 10.1021/acs.joc.6c00763

**Published:** 2026-06-01

**Authors:** Brais Vidal, Claudio Martínez, Rosana Álvarez, Ángel R. de Lera

**Affiliations:** CINBIO, Departamento de Química Orgánica, 16784 Universidade de Vigo, IBIV, As Lagoas-Marcosende, 36310 Vigo, Spain

## Abstract

Alternative tactics for polyene construction have been explored for the total synthesis of lipofuscin fluorophores A2E and pentadienyl-extended analog pdA2E, starting from properly functionalized pyridines. The bidirectional acyclic cross-metathesis (ACM) reaction of a C2-dienyl,C4-vinylpyridine with an excess of a conjugated tetraene unexpectedly afforded a C2-dienyl,C4-tetraenylpyridine with extended conjugation at just one of the branches. Although the stepwise process involving *N*-alkylation and a second ACM of the resulting pyridinium ion with the same tetraene generated A2E, the inconsistent results of the last ACM led to completing the skeleton using instead a Horner–Wadsworth–Emmons (HWE) condensation reaction of the C2-pentadienal-functionalized pyridine with a trienylphosphonate. The ACM proceeded even in lower yields in this case, thus showing the limitations of ACM for polyene synthesis. As an alternative, the C4-tetraenyl- and C4-hexaenylpyridine branches present in A2E and pdA2E were constructed by a Suzuki–Miyaura cross-coupling of a pyridine alkenylboronate and a trienyl- or a pentaenyl iodide, respectively, while extending the common C2-pentaenyl arms by a HWE reaction. Pyridine alkylation completed the synthesis of the lipofuscin fluorophores and confirmed the stereostructure of pdA2E, namely, the double-bond geometries and the relative location of the longer unsaturated chain on the pyridinium ring.

## Graphical abstract

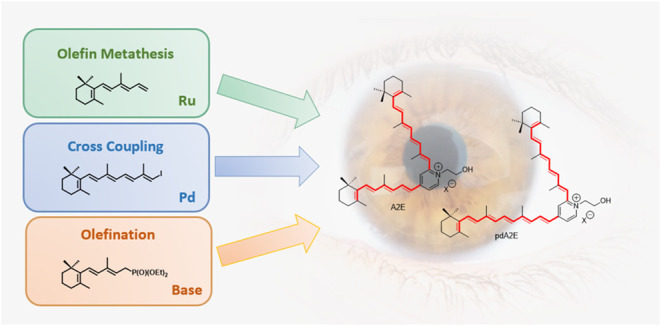

## Introduction

The visual process in vertebrates^1–3^ starts with the light-induced activation of the photoreceptors, which contain a protonated Schiff base derived from the condensation of 11-*cis*-retinal (**1**, Figure 1) and a lysine group (Lys296) of the protein opsin, the classical seven-transmembrane α-helical G-protein-coupled receptor. Light absorption triggers the visual cycle by first inducing the isomerization of the chromophore and the release of all-*trans*-retinal (**2**).^4^ The regeneration of the visual chromophore **1** to ensure sustained vision involves instead a complex phototransduction pathway or retinoid cycle, with formation of all-*trans*-retinyl esters (**4**) after reduction to all-*trans*-retinol (**3**) by retinol dehydrogenase 8 (Rdh8) in the outer segments of the visual rods and cones, the one-step conversion to 11-*cis*-retinol (**5**)^2^ promoted by isomerohydrolase retinal pigment epithelium-specific 65 kDa protein (RPE65),^2,5–7^ and final oxidation by retinol dehydrogenase 5 (Rdh5).

**Figure 1:**
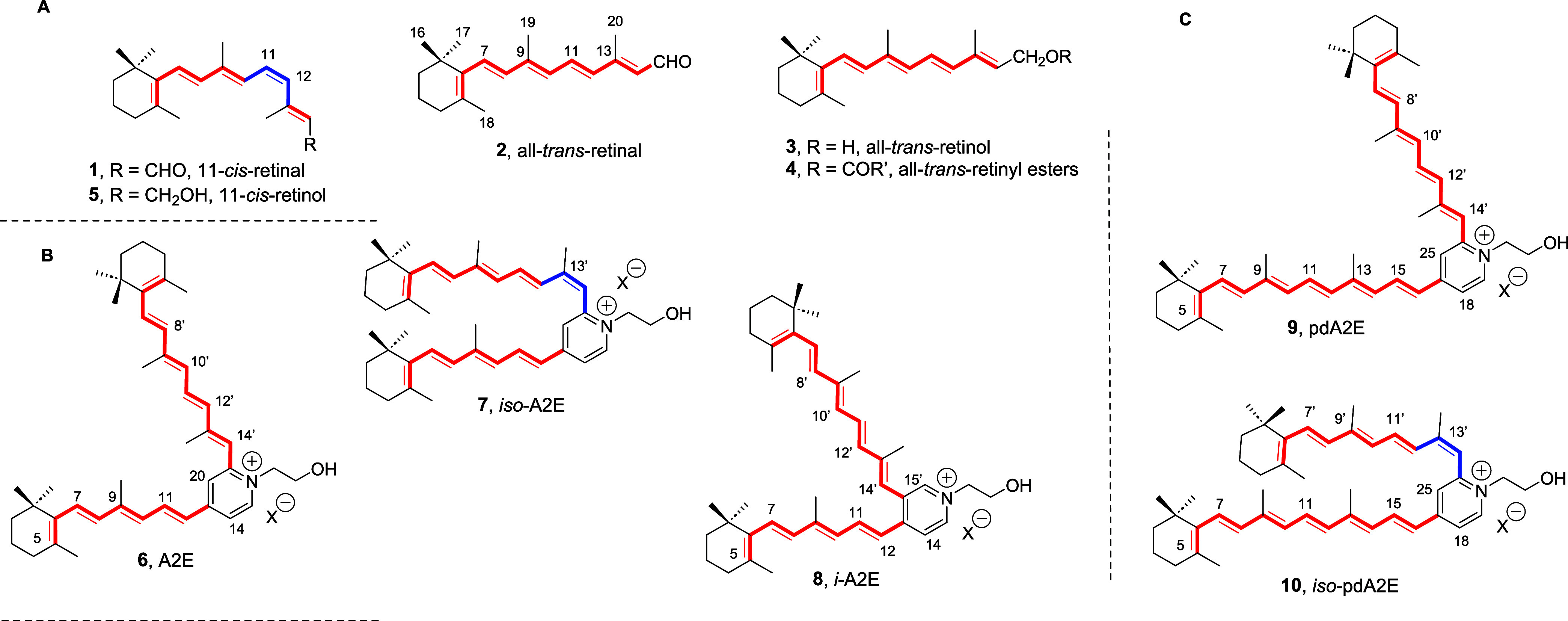
(A) Structures of retinoids implicated in the visual cycle. (B) Structures of bisretinoids with unsaturated fragments as substituents of *N*-pyridinium skeletons. (C) Pentadienyl-extended *N*-pyridinium bisretinoids. Numbering of the *N*-pyridinium bisretinoids follows that of retinoids (indicated for **2**) as shown on parent A2E (**6**),^12^ whereas those of pdA2E (**9**) and *iso*-pdA2E (**10**) formally extend the unsaturated fragment of A2E (**6**) with a pentadiene unit at the pyridine C4 position.

When these metabolic routes are perturbed,^8^ all-*trans*-retinal (**2**) can remain unprocessed and produce a buildup of so-called lipofuscin,^9^ the pro-inflammatory lipid-containing granules of fluorescent retinoids, lipids, and protein debris, considered partially responsible for age-related macular degeneration (AMD). Lipofuscin accumulates in the phagolysosomes of the retinal pigment epithelium (RPE), the monolayer adjacent to photoreceptor cells, and becomes one of the causative factors of blindness^10^ since it inhibits the visual cycle through direct interaction with RPE65.^2,5^

*N*-Retinylidene-*N*-retinylethanolamine or A2E (**6**),^7,10–13^ the most abundant of the lipofuscin fluorophores, was first structurally characterized and shown to be formed by enzymatic hydrolysis in the photoreceptor red outer segment membrane of the precursor A2PE,^6,7,14^ which results from the condensation of two molecules of all*-trans*-retinal (**2**) with dipalmitoyl-L-β-phosphatidylethanolamine.^15^ A ca. 4:1 photostationary equilibrium mixture of A2E (**6**) and its C13 = C14 *cis* (*Z*)-isomer, namely *iso*-A2E (**7**), has been isolated from human RPE cells and shown to be present upon irradiation of eye extracts.^7,13,16^

Additional ocular bisretinoids of lipofuscin (Figure 1B), including the positional isomer *i*-A2E (**8**)^17^ (first characterized as the 13′*Z* isomer and named *iiso*-A2E upon isolation, not shown)^18^ and formal pentadienyl-extended analogs, namely pdA2E (**9**)^19,20^ and its 13′*Z* isomer *iso*-pdA2E (**10**), have more recently been isolated in minute amounts from human eyes.^19,20^

The production of larger amounts (5.8% yield) of pdA2E (**9**) required incubation of all-*trans*-retinal (**2**) with excess ethanolamine in the absence of acetic acid for 4 days and HPLC purification from the mixture of products. Upon structural characterization, the new eye pigment was named pdA2E (**9**) since it is the result of formally adding a pentadiene fragment to the structure of parent A2E (**6**).^19,20^ Thus, when compared to A2E (**6**), both pdA2E (**9**) and its isomer *iso*-pdA2E (**10**) were proposed to feature an extended hexaenyl chain at the C4 position of the pyridinium skeleton, while preserving the pentaenyl chain at C2.

Given their relevant biological role in ARMD and its low percentage in the complex mixture of compounds that are present in the biological media, we targeted pdA2E (**9**),^19,20^ as a follow-up of our ongoing work on the stereocontrolled synthesis of some of the ocular bisretinoids of lipofuscin.^17,21^ In this regard, we have recently reported the synthesis and structural reassignment of the positional isomer *i*-A2E (**8**)^17^ and also the synthesis of enantiopure oxidized derivatives of A2E (not shown),^21^ namely, the first proposed mono-oxoA2E^22^ or furanoxide-A2E^23^ at each of the conjugated units, as well as the 5,8,5′,8′-bis-furanoxide-A2E^24^ using as a key step the Horner–Wadsworth–Emmons (HWE) condensation reaction.^25–28^

Although A2E (**6**) can be prepared in good yield (50–60%) by treatment of all-*trans*-retinal (**2**) with ethanolamine,^16^ under similar conditions, the amounts of the *cis*-isomer **7** ^16^ and those of *i*-A2E (**8**), pdA2E (**9**), and *iso*-pdA2E (**10**) as indicated above were shown to be very small, and the direct synthetic approach from **2** was deemed to be impractical.^19,20^

Being the proposed structure of pdA2E (**9**),^19,20^ the one corresponding to the pentadienyl-extended analog at C4 of the fluorescent pigment A2E (**6**) while sharing the C2-pentaenyl arm, we decided to first address the synthesis of A2E (**6**) as a model system for optimization purposes. Two total syntheses^12,29^ (for a formal synthesis, see ref^30^) have been developed (Scheme 1A) starting from appropriately functionalized 2,4-disubstituted pyridines (**12** and **14**), which construct both conjugated chains by either the Wittig reaction^12^ or Stille–Migita–Kosugi cross-coupling^29^ with the corresponding partners (**13** and **15**, respectively).

**Scheme 1:**
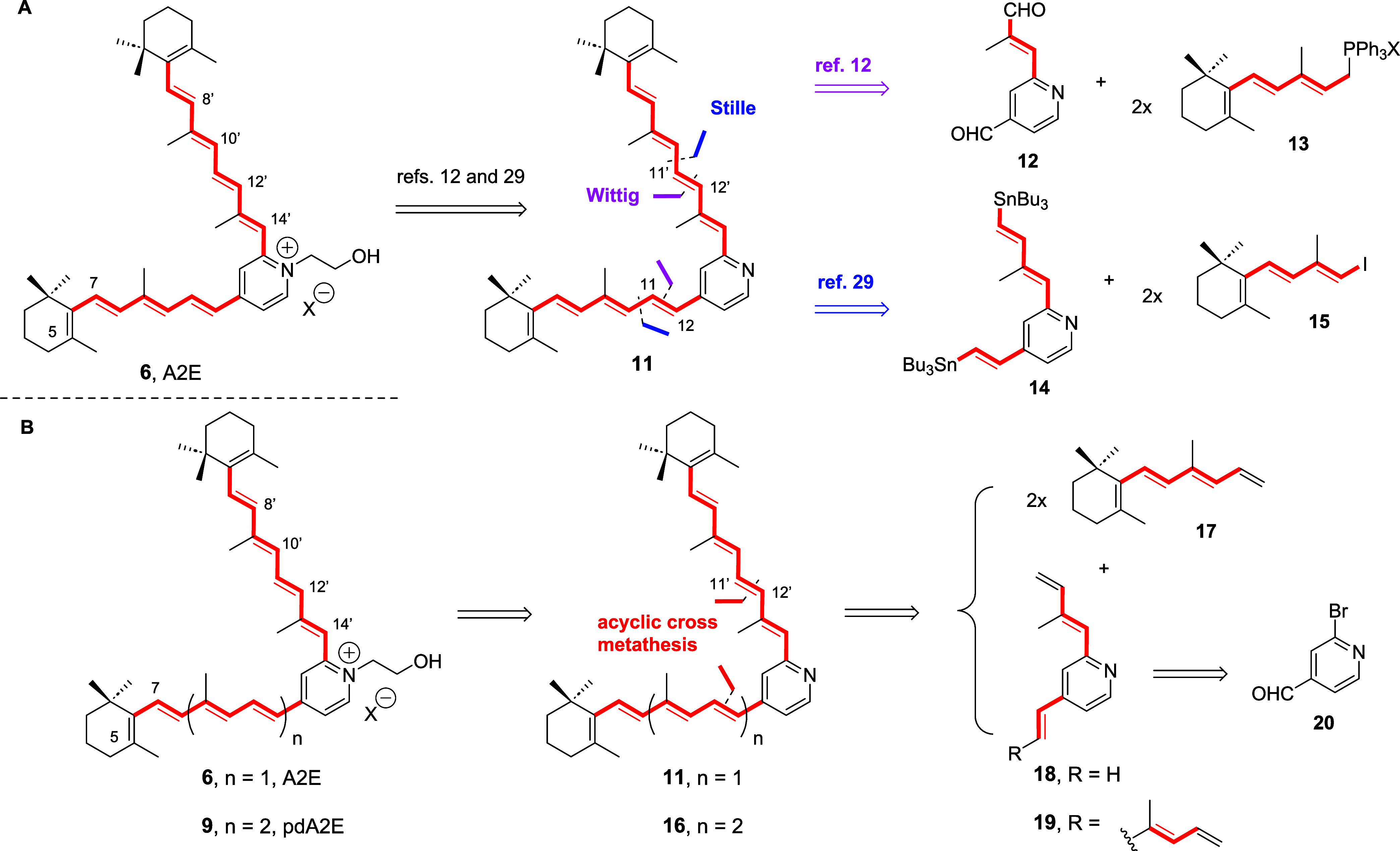
(A) Former Retrosynthetic Analysis of A2E (6). (B) ACM-Based Retrosynthetic Analysis of A2E (6) and pdA2E (9)

## Results and Discussion

Departing from these synthetic approaches to A2E (**6**),^12,29^ the synthesis of pyridine precursors of ocular bisretinoids **6** and **9** (**11** and **16**, respectively) was envisaged to be carried out using the acyclic cross-metathesis^31^ of common terminal tetraene **17** ^32^ and pyridines **18** and **19**, which incorporate the required unsaturated fragments as substituents at the C4 and C2 positions (Scheme 1B).

We have previously demonstrated that the acyclic cross-metathesis (ACM)^33–36^ of unsaturated components is a useful synthetic approach to polyenic structures including carotenoids containing up to 11 conjugated double bonds.^32,37^ However, alkenyl-substituted pyridines have rarely been used in metathesis processes,^38–40^ and therefore, no precedents on their ACM with complex polyenes such as **17** could be found.

The synthesis of pyridines bis-functionalized with two unsaturated fragments (**12** and **14**, Scheme 1A) has previously been carried out en route to A2E (**6**) starting from either 2-bromo-4-methylpyridine^12^ or 2,4-dibromopyridine.^29^ Our approach to **12** and **22** considered instead the use of commercial 2-bromopyridine-4-carboxaldehyde (**20**), which, properly functionalized, could allow us to extend the C4 branch by a Wittig methylenation^25,41,42^ and to construct the C2-unsaturated substituent by either a Suzuki–Miyaura cross-coupling reaction^43–45^ with commercial 2-(methylprop-1-en-1-yl)­boronic acid (**21**) followed by selective oxidation at the allylic position or by a Stille–Migita–Kosugi cross-coupling reaction^46–49^ with (*E*)-2-methyl-3-(tri-*n*-butylstannyl)­acrylaldehyde (**25**).^50^

In the event, upon heating a mixture of **20** and alkenylboronic acid **21** in 1,4-dioxane containing Pd­(OAc)_2_, PPh_3_, and Na_2_CO_3_ to 95 °C for 12 h, derivative **22** ^12^ was obtained in 87% yield. Methylenation of **22** was carried out in moderate yield (47%) by the Wittig reaction^25,41,42^ with the phosphorane reagent generated upon treatment of Ph_3_PCH_3_Br with KHMDS in THF/DMPU at 0 °C. Allylic oxidation of **23** with SeO_2_ in dioxane at 100 °C for 24 h and further stirring the reaction mixture with NaHCO_3_ for 2 h led to enal **24**, albeit in modest yields (49%). A subsequent Wittig olefination^25,41,42^ under the same conditions provided (58% yield) the diene substituent of C2-pentadienyl,C4-ethenylpyridine **18**.

Alternatively, the reaction of **20** with alkenylstannane **25** ^51^ under catalysis of Pd­(PPh_3_)_2_Cl_2_ in toluene at 110 °C afforded, after 20 h, dialdehyde **12** ^12,30,52^ in 54% yield. This intermediate could also be obtained in a similar yield through SeO_2_-promoted allylic oxidation of **22**.^12^ Bidirectional Wittig methylenation^25,41,42^ of dialdehyde **12** as indicated above gave rise efficiently (95%) to the required (*E*)-2-(2-methylbuta-1,3-dien-1-yl)-4-vinylpyridine (**18**), being therefore a most convenient approach.

In our experience, the second-generation Hoveyda–Grubbs II catalyst (HGII cat) **26** ^53^ proved to be the most appropriate for the acyclic cross-metathesis reaction of precursor hexaenes to afford the conjugated undecaenyl structures of polyenic carotenoids and apocarotenoids.^32,37^ Unfortunately, all attempts to carry out the metathesis of **18** and excess (5 equiv) of **17** in CH_2_Cl_2_ under these conditions^32,37,53^ led to the recovery of the components even upon heating up to 70 °C. It has been concluded from prior reports^38–40^ that *N*-heteroaromatics have a deleterious impact on metathesis by coordination and ligand displacement, inducing the deactivation of the ruthenium catalyst. The formation of bis-pyridine ruthenium carbene complexes has also been reported^54–59^ and some of them were found to undergo decomposition upon heating at 60 °C, leading to the assumption that donor ligands capable of replacing the phosphine and stabilizing the methylidene species, most notably amines and heteroaromatics,^38–40^ could cause deactivation of the catalyst.^54–59^

Considering the putative interference of the pyridine nitrogen^38–40^ with the ruthenium catalysts,^54–59^ the successful precedents on ACM of pyridinium salts,^60,61^ and our own experience on RCM reactions of bispyridinium dienes,^62^ we decided to first perform the alkylation of **18** with either 2-iodoethanol (**29**) or its TBS-protected derivative (not shown), but all attempts to promote the process even after heating in nitromethane at 100 °C proved unfruitful.

However, when the pyridine nitrogen was presumably forming a complex^63^ upon treatment with BF_3_ · OEt_2_ in dichloroethane or dichloroethane for 15 min,^64,65^ the addition of excess tetraene **17** (5 mol equivalents) and HGII catalyst (**26**)^53^ and further heating the resulting mixture at 50–60 °C for several hours provided a cross-metathesis product **28** in variable yields (7–71%) (see Table 1, S.I.). The use of nitro-Grela catalyst (**27**)^66^ in dichloroethane was more reliable, but the yield of **28** was 43% (see Table 1, S.I.).

Surprisingly, this compound showed ^1^H NMR signals corresponding to only one trimethylcyclohexenetrienyl fragment within the extended unsaturated arms attached to the pyridine nucleus. The proposed positional selectivity of the ACM reaction was determined by heteronuclear multiple bond correlation (HMBC) NMR analysis of the spectra of **28** in CD_2_Cl_2_, which confirmed the proximity of H-12 to both H-14 and H-20 pyridine atoms on the tetraenyl side chain (A2E numbering, see Figure 1). Moreover, the maximum wavelength of the UV absorption spectra (λ_max_ 347 nm in MeOH) is like the value assigned to the tetraenylpyridinium fragment present in A2E (**6**) (cf., for A2E,^12^ the λ_max_ values are 439 and 336 nm). These reaction conditions eventually promoted also the thermal isomerization of the mixture of double-bond isomers^12^ putatively generated in the ACM reaction.

All attempts to force the second ACM, even after heating the mixture up to 70 °C, led to partial recovery of the reactants as well as sample degradation. Interestingly, when the pyridine derivative **28** was subjected to *N*-alkylation with 2-iodoethanol (**29**) in nitromethane at 100 °C for 18h, pyridinium salt **30** could now be generated. Without further purification, intermediate **30** was treated with conjugated tetraene **17** under similar cross-metathesis reaction conditions with **26** or **27** at 60 °C for 20 h but in dichloromethane/DMF mixtures, and the bispolyenic pyridinium salt A2E (**6**) was formed in 30% combined yield (Scheme 2).

**Scheme 2:**
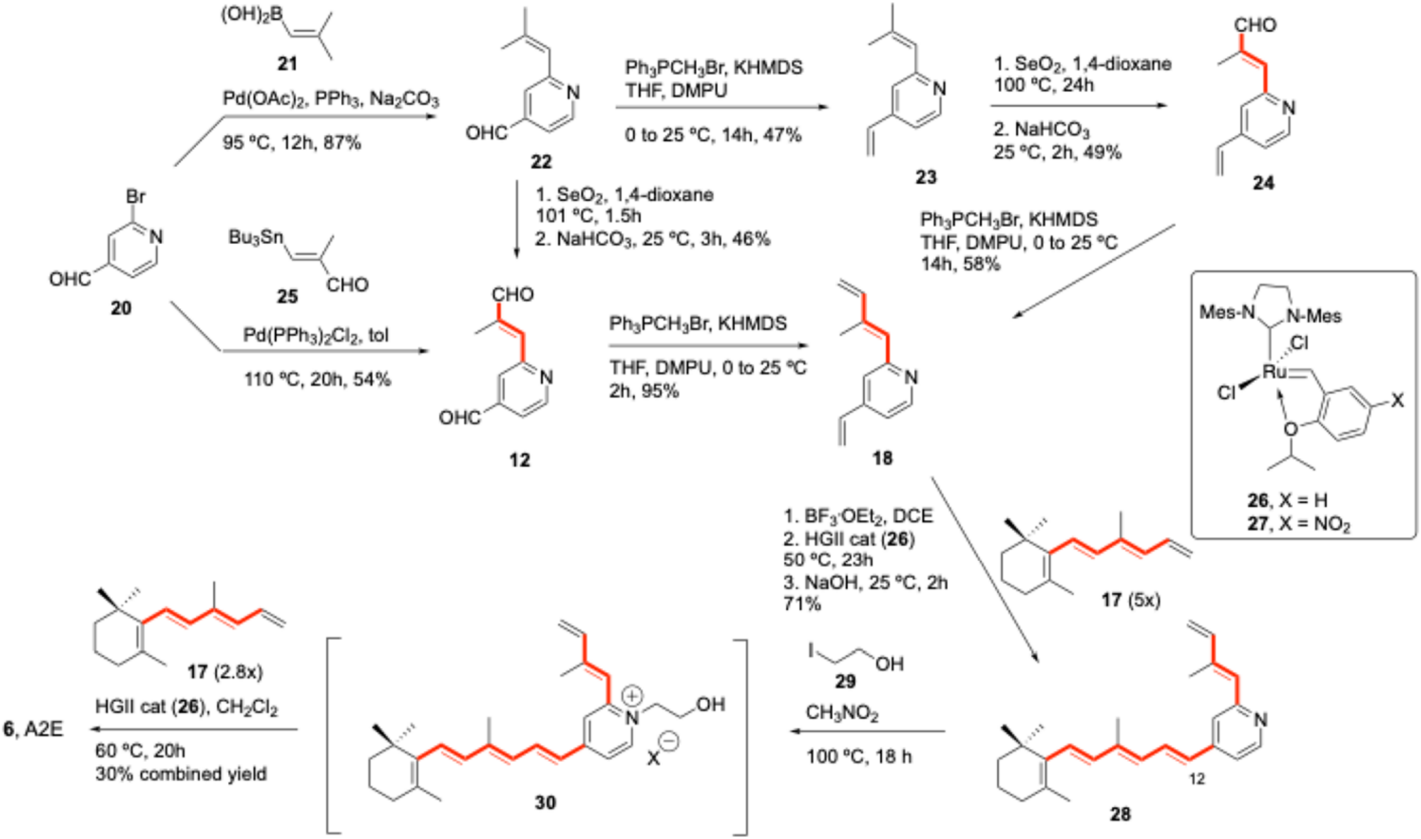
ACM-Based Synthesis of A2E (6)

However, and much to our dismay, upon attempts to improve the efficacy of the process and to establish the ACM reaction of these substrates as a general protocol, we noticed the capricious nature of the reaction with either **17** or the C4-polyenyl pyridinium salt (namely, **30**), which was found to be highly dependent on the quality and aging of the ruthenium catalyst (with the results using **27** being more reliable), leading in many experiments to recovery of starting materials or degradation of the samples.

Thus, a new approach to A2E (**6**) was considered, which replaced the second ACM with a stereochemically reliable Horner–Wadsworth–Emmons (HWE) condensation reaction.^25–28,67,68^ Our prior experience on carotenoids^69^ confirmed the efficacy of the classical HWE reaction,^25–28,67,68^ for polyene construction, which in this case would require the use of trienylphosphonate **40** (Scheme 3) as an alternative partner to construct the C11 = C12 disubstituted double bond of the longer unsaturated branch of bispolyenic pyridinium salt **6**.

**Scheme 3:**
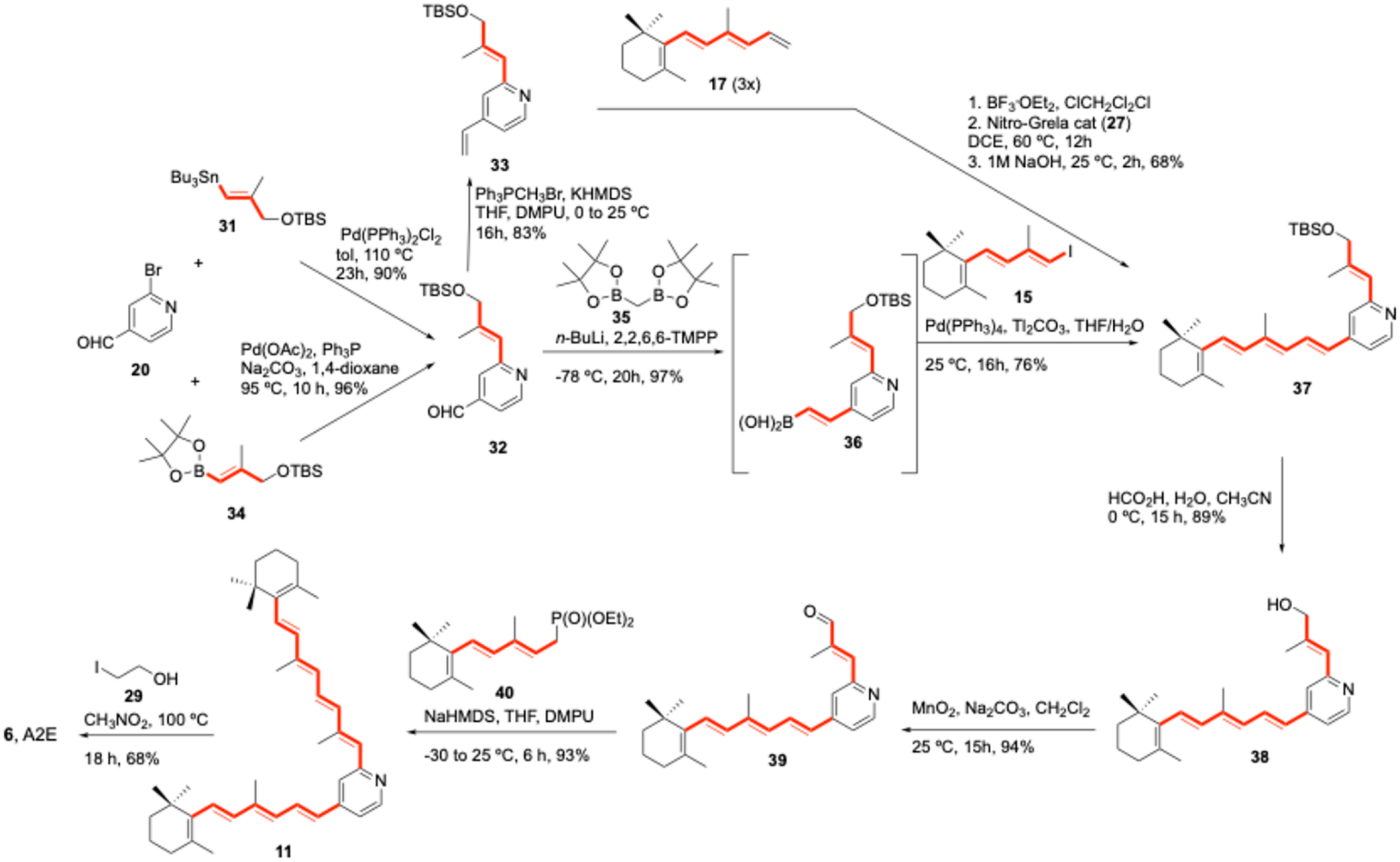
Alternative Synthetic Approaches to A2E (6)

The functionalized C2-alkenylpyridine derivative **32** could be formed in high yields using either the Stille cross-coupling reaction of **20** with protected (*E*)-3-tributylstannyl-2-methylpropenol (**31**)^70^ (90% yield) or the Suzuki–Miyaura cross-coupling^43–45^ with alternative partner **34** ^21^ (96% yield) followed by a Wittig-based methylenation reaction (83% yield). Under the best conditions, using an excess of tetraene **17** (3 mol equivalents), the ACM promoted by the nitro-Grela catalyst (**27**),^66^ in dichloroethane at 60 °C for 23 h afforded the C4-tetraenyl-substituted pyridine **37** in 68% yield (Scheme 3).

In order to employ the more reliable Pd-catalyzed Suzuki–Miyaura cross-coupling reaction^43–45^ to reach A2E (**6**), alkenyl extension at the C4 position of the pyridine ring was carried out by treatment of pyridine carbaldehyde **32** with bis­[(pinacolato)­boryl]­methane (**35**) and lithium 2,2,6,6-tetramethylpiperidine using the boron-Wittig olefination variant,^71^ which proceeded in almost quantitative yield and stereoselectively afforded alkenylboronic acid **36** as the *E* isomer.

We have already reported^17^ that the reaction conditions of the boron methylenation reaction of related pyridine carbaldehydes generated the corresponding boronic acids as reaction products, which might be related to the involvement of the pyridine nitrogen to form activated boronates, themselves more prone to undergo hydrolysis under the workup conditions. Upon stirring solutions of **36** and trienyl iodide **15** ^72^ in THF/H_2_O solvent mixtures in the presence of catalytic amounts of Pd­(PPh_3_)_4_ and promoted by Tl_2_CO_3_, targeted C2-alkenyl,C4-tetraenylpyridine **37** was formed in 76% yield.

The C2-unsaturated branch of A2E (**6**) was completed from disubstituted pyridine **37** by silyl ether deprotection under acidic conditions (89% yield), oxidation of **38** with manganese oxide (94% yield), and a highly efficient (93% yield) HWE condensation reaction of **39** with trienylphosphonate **40** using an excess (1.7 mol equivalents) of the anion generated with NaHMDS (1.5 mol equivalents) in THF and DMPU, as previously demonstrated with structurally related bis-polyenylpyridines.^17^ Uneventful alkylation of **11** with 2-iodoethanol (**29**) as indicated above led finally to A2E (**6**) in higher yields and synthetic reliability than using the ACM reaction (Scheme 3).

Exploring the efficiency of the synthetic approaches to these visual pigments,^17^ we then addressed the synthesis of pentadienyl-extended pyridinium salt pdA2E (**9**) in a similar manner. Having hexaenyl and pentaenyl unsaturated chains at C4 and C2, respectively, the retrosynthetic approach to pdA2E (**9**) would depart from that of A2E (**6**) depicted in Scheme 1B by using a C19-iodopentaenyl component (**43**) to connect to common alkenylboronic acid **36** prior to chain extension at C2. Trienylphosphonate **40** could be exploited as a partner to not only get **16** from **41** but also complete the skeleton of pentaenyl iodide **43** from iodoalkenal **42** (Scheme 4).

**Scheme 4:**
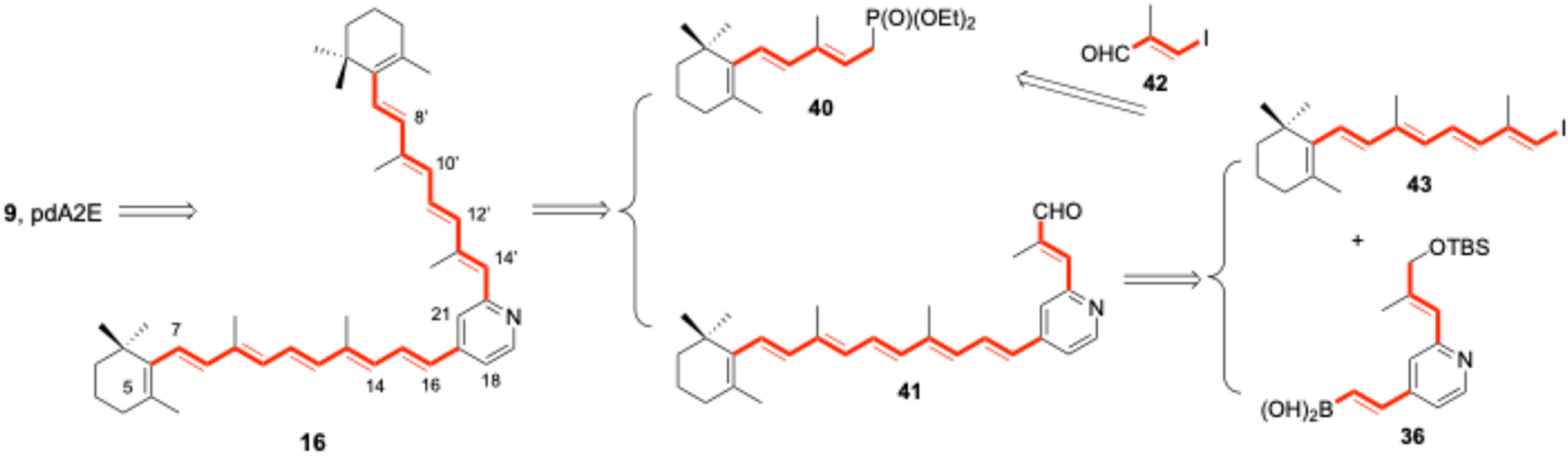
Retrosynthetic Analysis of pdA2E (9)

The optimized reaction conditions^17^ for the HWE condensation reaction^25–27,67,68,73^ of heptatrienylphosphonate **40** ^74–77^ and (*E*)-3-iodo-2-methylpropenal **42** ^78,79^ afforded the stereochemically homogeneous pentaenyl iodide **43** in 66% yield. The Suzuki–Miyaura cross-coupling reaction^43–45^ of **43** with alkenylboronic acid **36** promoted by Pd­(PPh_3_)_4_ and Tl_2_CO_3_ at ambient temperature constructed stereoselectively the hexaene branch of **44** in 72% yield (Scheme 5). Deprotection of **44** (HCO_2_H, H_2_O, 0 °C, 14h, 69%) was followed by oxidation of the allylic alcohol of **45** using MnO_2_ and Na_2_CO_3_ to afford enal **41** in 88% yield. The second HWE reaction^25–28,67,68^ of **41** with excess phosphonate **40** ^74–77^ under the same conditions described above was again highly stereoselective for the construction of the pentaene branch and afforded **16** in 94% yield. Pyridine alkylation of **16** with 2-iodoethanol (**29**) in nitromethane took place under relatively milder conditions by heating to 80 °C for 18h and generated pdA2E (**9**) in almost quantitative yield.

**Scheme 5:**
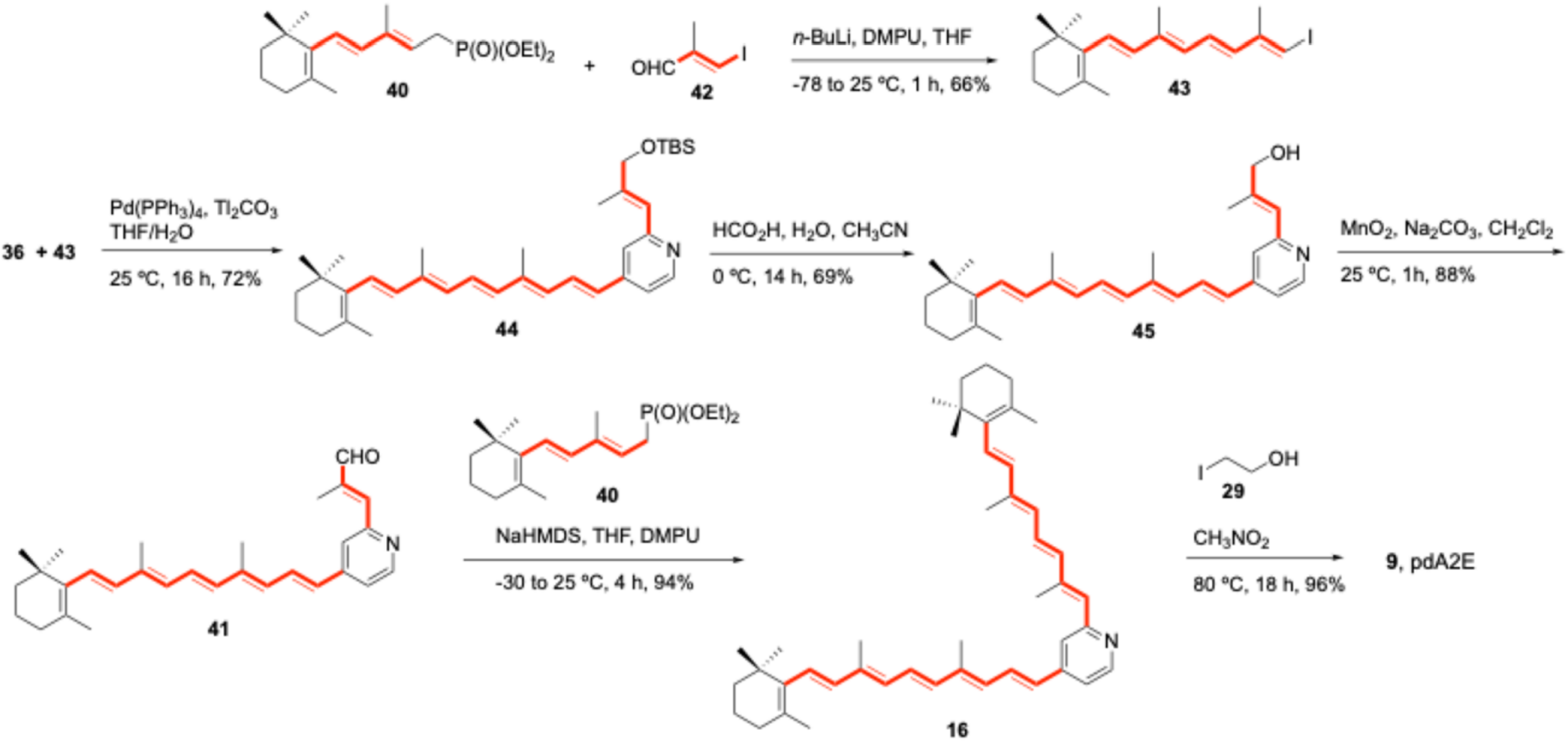
Stepwise HWE Reactions and Suzuki–Miyaura Cross-Coupling for the Total Synthesis of pdA2E (9)

The ^1^H NMR and ^13^C NMR spectroscopic data, including NOESY correlations, and the UV data of the synthetic sample matched those of the natural product.^19,20^ When compared to A2E (**6**), additional resonances in the ^1^H and ^13^C NMR spectra for a methyl group and three alkenyl hydrogens confirmed the presence of a C13–C16 pentadienyl fragment on the hexaenyl substituent at the C4 position of the pyridinium ion of pdA2E (**9**), in full consistency with the data for the proposed structure.^19,20^ The experimental absorption values in the UV spectra, namely, 338 nm (ε_M_ ≈ 35,700 M^–1^ · cm^–1^) and 490 nm (ε_M_ ≈ 43,800 M^–1^ · cm^–1^), were also coincidental with those described for natural pdA2E (**9**), 340 nm (ε_M_ ≈ 14,000 M^–1^ · cm^–1^) and 492 nm (ε_M_ ≈ 12,000 M^–1^ · cm^–1^).^19,20^

## Summary

AMD, the most common cause of acquired vision loss in the elderly, is characterized by the overload along life in the RPE of lipofuscin, a mixture of fluorescent bisretinoids, lipids, and protein debris, which causes photoreceptor cell loss in aging human eyes. Bis-polyenylpyridinium salt pdA2E, a byproduct or retinal metabolism, which has recently been isolated as a component of lipofuscin fluorophores from animal and human eyes and considered partially responsible for AMD, is a formal pentadienyl-extended pyridinium bisretinoid. pdA2E was proposed to contain a conjugated C4-hexaenyl branch on the pyridinium salt while preserving the C2-pentaenyl substituent of parent A2E (**6**). pdA2E (**9**) has been prepared as a minor component upon treatment of an excess of all-*trans*-retinal with ethanolamine in a process accelerated by acidic media. The stereocontrolled total synthesis of pdA2E (**9**) has been completed starting from 2,4-bis-functionalized pyridine **36**, after exploring synthetic methodologies using A2E (**6**) as a pyridinium bisretinoid model system. Although the stepwise and interrupted ACM processes of 2,4-dialkenylatedpyridine with excess tetraene **17** afforded A2E (**6**), the key bond formation step was found to be unreliable and hardly reproducible. A more efficient synthetic approach to A2E (**6**) combined the Suzuki–Miyaura cross-coupling reaction and the HWE condensation for construction of the C4-tetraenyl and C2-pentadienyl branches, respectively, ending with uneventful pyridine alkylation. This sequence was easily extended to pdA2E (**9**) by simply attaching the pentaenyl iodide **43** to the same reaction partner and exploiting similar functionalization strategies. Our work on the synthesis and mechanism of formation of positional isomers and stereoisomers of both A2E (**6**) and longer polyenic analogs such as pdA2E (**9**), aimed to shed some light on the putative role of these bisretinoids in ocular diseases, will also pave the way to get further insights into the biogenetic formation of pdA2E (**9**) by the reaction of bisretinoid pyridinium salt A2E (**6**) and another molecule of all-*trans*-retinal, as previously proposed.^19,20^

## Supplementary Material



## Data Availability

The data underlying this study are available in the published article and its Supporting Information.

## Notes

Experimental procedures, characterization data, and copies of NMR spectra (PDF)

## References

[1] Zhang J., Choi E. H., Tworak A., Salom D., Leinonen H., Sander C. L., Hoang T. V., Handa J. T., Blackshaw S., Palczewska G., Kiser P. D., Palczewski K. (2019). Photic generation
of 11-*cis*-retinal in bovine retinal pigment epithelium. J. Biol. Chem..

[2] Palczewski K., Kiser P. D. (2020). Shedding new light on the generation
of the visual chromophore. Proc. Natl. Acad.
Sci. U.S.A..

[3] Chen S., Getter T., Salom D., Wu D., Quetschlich D., Chorev D. S., Palczewski K., Robinson C. V. (2022). Capturing a rhodopsin
receptor signalling cascade across a native membrane. Nature.

[4] Gruhl T., Weinert T., Rodrigues M. J., Milne C. J., Ortolani G., Nass K., Nango E., Sen S., Johnson P. J. M., Cirelli C., Furrer A., Mous S., Skopintsev P., James D., Dworkowski F., Båth P., Kekilli D., Ozerov D., Tanaka R., Glover H., Bacellar C., Brünle S., Casadei C. M., Diethelm A. D., Gashi D., Gotthard G., Guixà-González R., Joti Y., Kabanova V., Knopp G., Lesca E., Ma P., Martiel I., Mühle J., Owada S., Pamula F., Sarabi D., Tejero O., Tsai C.-J., Varma N., Wach A., Boutet S., Tono K., Nogly P., Deupi X., Iwata S., Neutze R., Standfuss J., Schertler G., Panneels V. (2023). Ultrafast structural changes direct
the first molecular events of vision. Nature.

[5] Moiseyev G., Nikolaeva O., Chen Y., Farjo K., Takahashi Y., Ma J.-x. (2010). Inhibition of the visual cycle by A2E through direct interaction
with RPE65 and implications in Stargardt disease. Proc. Natl. Acad. Sci. U.S.A..

[6] Sparrow J. R., Gregory-Roberts E., Yamamoto K., Blonska A., Ghosh S. K., Ueda K., Zhou J. (2012). The bisretinoids of retinal pigment
epithelium. Prog. Retinal Eye Res..

[7] Kim H. J., Sparrow J. R. (2021). Bisretinoid phospholipid
and vitamin A aldehyde: shining light. J. Lipid
Res..

[8] Yakovleva M. A., Radchenko A. S., Feldman T. B., Kostyukov A. A., Arbukhanova P. M., Borzenok S. A., Kuzmin V. A., Ostrovsky M. A. (2020). Fluorescence
characteristics of lipofuscin fluorophores from human retinal pigment
epithelium. Photochem. Photobiol. Sci..

[9] Murdaugh L. S., Mandal S., Dill A. E., Dillon J., Simon J. D., Gaillard E. R. (2011). Compositional studies
of human RPE lipofuscin: mechanisms of molecular modifications. J. Mass Spectrom..

[10] Mata N. L., Weng J., Travis G. H. (2000). Biosynthesis of a major lipofuscin
fluorophore in mice and humans with ABCR-mediated retinal and macular
degeneration. Proc. Natl. Acad. Sci. U.S.A..

[11] Sakai N., Decatur J., Nakanishi K. (1996). Ocular Eye
Pigment ″A2-E″: An Unprecedented Pyridinium Bisretinoid. J. Am. Chem. Soc..

[12] Ren R. X.-F., Sakai N., Nakanishi K. (1997). Total Synthesis
of the Ocular Age Pigment A2-E: A Convergent Pathway. J. Am. Chem. Soc..

[13] Sparrow J. R., Fishkin N., Zhou J., Cai B., Jang Y. P., Krane S., Itagaki Y., Nakanishi K. (2003). A2E, a byproduct
of the visual cycle. Vision Res..

[14] Wu Y., Zhou J., Fishkin N., Rittmann B. E., Sparrow J. R. (2011). Enzymatic Degradation of A2E, a Retinal
Pigment Epithelial Lipofuscin Bisretinoid. J.
Am. Chem. Soc..

[15] Bui T. V., Han Y., Radu R. A., Travis G. H., Mata N. L. (2006). Characterization
of Native Retinal Fluorophores Involved in Biosynthesis of A2E and
Lipofuscin-associated Retinopathies. J. Biol.
Chem..

[16] Parish C. A., Hashimoto M., Nakanishi K., Dillon J., Sparrow J. (1998). Isolation
and one-step preparation of A2E and *iso*-A2E, fluorophores
from human retinal pigment epithelium. Proc.
Natl. Acad. Sci. U.S.A..

[17] Vidal B., Iglesias-Menduiña O., Vaz B., Álvarez R., Martínez C., de Lera Á. R. (2025). Stereochemical
Reassignment by Total Synthesis of an Ocular Pyridinium Bisretinoid
of Retinal Pigment Epithelium Lipofuscin: iiso-A2E Is i-A2E. Org. Lett..

[18] Li J., Yao K., Yu X., Dong X., Gan L., Luo C., Wu Y. (2013). Identification of a Novel Lipofuscin Pigment (*iiso*A2E) in Retina and Its Effects in the Retinal Pigment
Epithelial Cells. J. Biol. Chem..

[19] Zhao J., Yao K., Jin Q., Jiang K., Chen J., Liu Z., Li J., Wu Y. (2014). Preparative and Biosynthetic Insights Into pdA2E and *iso*pdA2E, Retinal-Derived Fluorophores of Retinal Pigment Epithelial
Lipofuscin Retinal-Derived Fluorophores pdA2E and *iso*pdA2E. Invest. Ophthamol. Vis. Sci..

[20] Wu Y., Jin Q., Yao K., Zhao J., Chen J., Wu X., Gan L., Li J., Song X., Liu X., Cai X. (2014). Retinal metabolism
in humans induces the formation of an unprecedented lipofuscin fluorophore
“pdA2E”. Biochem. J..

[21] Vidal B., Rodríguez R., Peña-Gallego A., Álvarez R., Martínez C., de Lera Á. R. (2026). Enantiopure Pyridinium Bisretinoids of Ocular Lipofuscin
with Hexahydrobenzofuran Structure: Total Synthesis and Structure-Dependent
Aggregated Morphology. J. Org. Chem..

[22] Ben-Shabat S., Itagaki Y., Jockusch S., Sparrow J. R., Turro N. J., Nakanishi K. (2002). Formation
of a Nonaoxirane from A2E, a Lipofuscin Fluorophore related to Macular
Degeneration, and Evidence of Singlet Oxygen Involvement. Angew. Chem., Int. Ed..

[23] Avalle L. B., Wang Z., Dillon J. P., Gaillard E. R. (2004). Observation of A2E oxidation products in human retinal
lipofuscin. Exp. Eye Res..

[24] Dillon J., Wang Z., Avalle L. B., Gaillard E. R. (2004). The photochemical oxidation of A2E results in the formation
of a 5,8,5′,8′-bis-furanoid oxide. Exp. Eye Res..

[25] Nicolaou K. C., Härter M. W., Gunzner J. L., Nadin A. (1997). The Wittig
and Related Reactions in Natural Product Synthesis. Liebigs Ann..

[26] Kobayashi K., Tanaka K., Kogen H. (2018). Recent topics of the
natural product synthesis by Horner–Wadsworth–Emmons
reaction. Tetrahedron Lett..

[27] Roman D., Sauer M., Beemelmanns C. (2021). Applications
of the Horner–Wadsworth–Emmons Olefination in Modern
Natural Product Synthesis. Synthesis.

[28] Babar J., Ahmad S., Parveen B., Ali K. G., Mushtaq A., Zahoor A. F., Ahmad R., Mansha A., Irfan A. (2025). Exploring the Synthetic Potential
of Horner-Wadsworth-Emmons Reaction Toward the Synthesis of Polyketide
Based Natural Products: A Review. Top. Curr.
Chem..

[29] Sicre C., Cid M. M. (2005). Convergent Stereoselective
Synthesis of the Visual Pigment A2E. Org. Lett..

[30] Tanaka K., Katsumura S. (2000). Novel Synthesis
of the Ocular Age Pigment A2E: New Method for Substituted Pyridine
Synthesis via Azaelectrocyclization. Org. Lett..

[31] Chatterjee A. K., Choi T.-L., Sanders D. P., Grubbs R. H. (2003). A General Model for Selectivity in Olefin Cross Metathesis. J. Am. Chem. Soc..

[32] Fontán N., Domínguez M., Álvarez R., de Lera Á. R. (2011). Synthesis
of C40-Symmetrical Fully Conjugated Carotenoids by Olefin Metathesis. Eur. J. Org. Chem..

[33] Grubbs, R. H. Handbook of Metathesis; Wiley-VCH: Weinheim, 2003.

[34] Nicolaou K.
C., Bulger P. G., Sarlah D. (2005). Metathesis Reactions in Total Synthesis. Angew. Chem., Int. Ed..

[35] Hoveyda A. H., Zhugralin A. R. (2007). The remarkable metal-catalyzed olefin metathesis reaction. Nature.

[36] Fürstner A. (2011). Metathesis
in total synthesis. Chem. Commun..

[37] Domínguez M., Pequerul R., Alvarez R., Giménez-Dejoz J., Birta E., Porté S., Rühl R., Parés X., Farrés J., de Lera A. R. (2018). Synthesis of apocarotenoids by acyclic cross metathesis
and characterization as substrates for human retinaldehyde dehydrogenases. Tetrahedron.

[38] Wilson G. O., Porter K. A., Weissman H., White S. R., Sottos N. R., Moore J. S. (2009). Stability of Second Generation Grubbs’
Alkylidenes to Primary Amines: Formation of Novel Ruthenium-Amine
Complexes. Adv. Synth. Catal..

[39] Lafaye K., Bosset C., Nicolas L., Guérinot A., Cossy J. (2015). Beyond catalyst deactivation: cross-metathesis
involving olefins containing N-heteroaromatics. Beilstein J. Org. Chem..

[40] Ireland B. J., Dobigny B. T., Fogg D. E. (2015). Decomposition of a Phosphine-Free
Metathesis Catalyst by Amines and Other Bronsted Bases: Metallacyclobutane
Deprotonation as a Major Deactivation Pathway. ACS Catal..

[41] Hoffmann R. W. (2001). Wittig
and His Accomplishments: Still Relevant Beyond His 100th Birthday. Angew. Chem., Int. Ed..

[42] Farfán P., Gómez S., Restrepo A. (2019). Dissection of the Mechanism of the Wittig Reaction. J. Org. Chem..

[43] Miyaura N., Suzuki A. (1995). Palladium-Catalyzed Cross-Coupling Reactions of Organoboron
Compounds. Chem. Rev..

[44] Suzuki A. (2005). Carbon-carbon bonding made easily. Chem. Commun..

[45] Suzuki A. (2011). Cross-Coupling Reactions Of Organoboranes:
An Easy Way To Construct C-C Bonds (Nobel Lecture). Angew. Chem., Int. Ed..

[46] Espinet P., Echavarren A. M. (2004). The Mechanisms of the Stille Reaction. Angew. Chem., Int. Ed..

[47] Heravi M. M., Hashemi E., Azimian F. (2014). Recent developments of the Stille
reaction as a revolutionized method in total synthesis. Tetrahedron.

[48] Hashemi E., Teimoury M. (2026). Advancing total synthesis through
the Stille cross-coupling: recent innovations and case studies. RSC Adv..

[49] Anwar L., Ahmad S., Ghulam Ali K., Parveen B., Zahoor A. F., Naqvi S. A. R., Ashraf J., Nazeer U. (2026). Recent synthetic innovations
in Stille coupling reaction: A review. Tetrahedron.

[50] Asano M., Inoue M., Watanabe K., Abe H., Katoh T. (2006). Synthetic Studies toward GKK1032s, Novel Antibiotic
Antitumor Agents: Enantioselective Synthesis of the Fully Elaborated
Tricyclic Core via an Intramolecular Diels–Alder Cycloaddition. J. Org. Chem..

[51] Lipshutz B. H., Clososki G. C., Chrisman W., Chung D. W., Ball D. B., Howell J. (2005). New Conjunctive Reagents as Cross-Coupling Partners
En Route to Retinoid-like Polyenes. Org. Lett..

[52] Tanaka K., Mori H., Yamamoto M., Katsumura S. (2001). Significant Acceleration of 6π-Azaelectrocyclization
Resulting from a Remarkable Substituent Effect and Formal Synthesis
of the Ocular Age Pigment A2-E by a New Method for Substituted Pyridine
Synthesis. J. Org. Chem..

[53] Garber S. B., Kingsbury J. S., Gray B. L., Hoveyda A. H. (2000). Efficient and Recyclable Monomeric
and Dendritic Ru-Based Metathesis Catalysts. J. Am. Chem. Soc..

[54] Sanford M. S., Love J. A., Grubbs R. H. (2001). A Versatile Precursor for the Synthesis
of New Ruthenium Olefin Metathesis Catalysts. Organometallics.

[55] Ung T., Hejl A., Grubbs R. H., Schrodi Y. (2004). Latent Ruthenium Olefin
Metathesis Catalysts That Contain an *N*-Heterocyclic
Carbene Ligand. Organometallics.

[56] Suriboot J., Hu Y., Malinski T. J., Bazzi H. S., Bergbreiter D. E. (2016). Controlled Ring-Opening Metathesis
Polymerization with Polyisobutylene-Bound Pyridine-Ligated Ru­(II)
Catalysts. ACS Omega.

[57] McClennan W. L., Rufh S. A., Lummiss J. A. M., Fogg D. E. (2016). A General Decomposition Pathway for Phosphine-Stabilized
Metathesis Catalysts: Lewis Donors Accelerate Methylidene Abstraction. J. Am. Chem. Soc..

[58] Polyanskii K. B., Alekseeva K. A., Raspertov P. V., Kumandin P. A., Nikitina E. V., Gurbanov A. V., Zubkov F. I. (2019). Hoveyda–Grubbs catalysts with
an N→Ru coordinate bond in a six-membered ring. Synthesis of
stable, industrially scalable, highly efficient ruthenium metathesis
catalysts and 2-vinylbenzylamine ligands as their precursors. Beilstein J. Org. Chem..

[59] Jawiczuk M., Marczyk A., Trzaskowski B. (2020). Decomposition of Ruthenium Olefin
Metathesis Catalyst. Catalysts.

[60] Fu G. C., Nguyen S. T., Grubbs R. H. (1993). Catalytic
ring-closing metathesis of functionalized dienes by a ruthenium carbene
complex. J. Am. Chem. Soc..

[61] Fürstner A., Leitner A. (2003). A Catalytic Approach
to (*R*)-(+)-Muscopyridine with Integrated “Self-Clearance”. Angew. Chem., Int. Ed..

[62] Pérez-Balado C., Rodríguez-Graña P., Nebiosso A., Minichiello A., Miceli M., Altucci L., de Lera A. R. (2007). Bispyridinium dienes,
a novel class of histone deacetylase inhibitors with selective activities. J. Med. Chem..

[63] Kessar S.
V., Singh P. (1997). Lewis Acid
Complexation of Tertiary Amines and Related Compounds: A Strategy
for α-Deprotonation and Stereocontrol. Chem. Rev..

[64] Jaric M., Haag B. A., Unsinn A., Karaghiosoff K., Knochel P. (2010). Highly Selective Metalations of Pyridines
and Related Heterocycles Using New Frustrated Lewis Pairs or tmp-Zinc
and tmp-Magnesium Bases with BF_3_·OEt_2_. Angew. Chem., Int. Ed..

[65] Chen Q., du Jourdin X. M., Knochel P. (2013). Transition-Metal-Free BF_3_-Mediated Regioselective Direct Alkylation and Arylation of Functionalized
Pyridines Using Grignard or Organozinc Reagents. J. Am. Chem. Soc..

[66] Grela K., Harutyunyan S., Michrowska A. (2002). A highly efficient ruthenium catalyst
for metathesis reactions. Angew. Chem., Int.
Ed..

[67] Horner L. (1964). Darstellung und Eigenschaften optisch
aktiver, tertiärer Phosphine. Pure Appl.
Chem..

[68] Wadsworth W. S. (1977). Synthetic
Applications of Phosphoryl-Stabilized Anions. Org. React..

[69] Rivas A., Castiñeira M., Álvarez R., Vaz B., de Lera A. R. (2022). Stereoselective
Synthesis of Bisfuranoxide (Aurochrome, Auroxanthin) and Monofuranoxide
(Equinenone 5′,8′-Epoxide) Carotenoids by Double Horner–Wadsworth–Emmons
Reaction. J. Nat. Prod..

[70] Burghart J., Sorg A., Brückner R. (2011). Stereocomplementary
Syntheses of 1,ω-Distannylated *E*, *Z*-Isomeric Conjugated Trienes, Tetraenes, and Pentaenes. Chem. - Eur. J..

[71] Coombs J. R., Zhang L., Morken J. P. (2015). Synthesis of Vinyl Boronates from
Aldehydes by a Practical Boron–Wittig Reaction. Org. Lett..

[72] Rivas A., Pérez-Revenga V., Alvarez R., de Lera A. R. (2019). Bidirectional Hiyama–Denmark Cross-Coupling
Reactions of Bissilyldeca-1,3,5,7,9-pentaenes for the Synthesis of
Symmetrical and Non-Symmetrical Carotenoids. Chem. - Eur. J..

[73] Gu, Y. ; Tian, S.-K. Olefination Reactions of Phosphorus-Stabilized Carbon Nucleophiles. In Stereoselective Alkene Synthesis; Wang, J. , Ed.; Springer Berlin, 2012; Vol. 327, pp 197–238. 10.1007/128_2012_314 22371171

[74] Acemoglu M., Prewo R., Bieri J. H., Eugster C. H. (1984). (6′*RS*,8′*RS*,2*E*)- und (6′*RS*,8′*SR*,2*E*)-3-Methyl-3-(2′,2′,6′-trimethyl-7′-oxabicyclo­[4.3.0]­non-9′-en-8′-yl)-2-propenal
([(5*RS*,8*RS*)- und (5*RS*,8*SR*)-5,8-Epoxy-5,8-dihydro-ionyloinden]­acetaldehyd):
Synthese und Röntgenstrukturanalyse. Helv. Chim. Acta.

[75] Acemoglu M., Eugster C. H. (1984). (5*R*,6*S*,5′*R*,6′*S*)-5,6,5′,6′-Diepoxy-β,β-cartin:
Synthese, Spectroskopische, chiroptische und chromatographische Eigenschaften. Helv. Chim. Acta.

[76] Azim E.-M., Auzeloux P., Maurizis J.-C., Braesco V., Grolier P., Veyre A., Madelmont J.-C. (1996). Synthesis
of all-*trans*-beta-carotene, retinoids and derivatives
labeled with ^14^C. J. Labelled Compd.
Radiopharm..

[77] Domínguez M., Alvarez R., Borrás E., Farrés J., Parés X., de Lera A. R. (2006). Synthesis of Enantiopure
C_3_- and C_4_-Hydroxyretinals and Their Enzymatic
Reduction by ADH8 from *Xenopus laevis*. Org. Biomol. Chem..

[78] Baker R., Castro J. L. (1990). Total synthesis of (+)-macbecin I. J. Chem. Soc., Perkin Trans..

[79] Menche D., Hassfeld J., Li J., Mayer K., Rudolph S. (2009). Modular Total
Synthesis of Archazolid A and B. J. Org. Chem..

