# Supplementary material for: Exploring Polyene Construction Tactics for the Total Synthesis of Ocular Pyridinium Bisretinoids of Lipofuscin A2E and pdA2E
Source: J Org Chem. 2026 Jun 1;91(23):7995–8003. doi: 10.1021/acs.joc.6c00763 (PMC13270528; doi:10.1021/acs.joc.6c00763)

Supporting information (S.I.)

Exploring Polyene Construction Tactics for the Total Synthesis of Ocular Pyridinium Bisretinoids of Lipofuscin A2E and pdA2E

Brais Vidal, Claudio Martínez, Rosana Álvarez,* and Ángel R. de Lera*

[a] CINBIO, Departamento de Química Orgánica, Universidade de Vigo, IBIV, As Lagoas-Marcosende, 36310 Vigo, Spain

qolera@uvigo.es

Table of Contents

1. General remarks S2
2. Optimization of the olefin metathesis reaction S3
3. Experimental procedures S4
3. Comparison of ^{1}H-NMR spectra of pdA2E (**9**) (natural) and pdA2E (**9**) (synthetic). S25
4. References S26
5. Copies of NMR spectra S27

1. General remarks

Solvents were dried using a Puresolv™ solvent purification system. All other reagents were commercial compounds of the highest purity available. If not specified, all reactions were carried out under an argon atmosphere. Those not involving aqueous reagents were carried out in oven dried glassware. For reactions that require heating, a metallic heating block was used and the indicated is the external temperature. All solvents and anhydrous solutions were transferred through syringes and cannulas previously dried in the oven for at least 12h and kept in a desiccator. Analytical TLC was performed on aluminium plates with Merck Kieselgel $60F_{254}$ and visualized by UV irradiation (254 nm) or by staining with a solution of phosphomolybdic acid in ethanol. Flash column chromatography was carried out using Merck Kieselgel 60 (230–400 mesh) with a CombiFlash® Rf Teledyne Isco.

HRMS (ESI^+) were measured with an FT-ICR-MS Solarix 7T mass spectrometer (Bruker Daltonics). ^{1}H-NMR spectra were recorded in $CDCl_3$, C_6D_6, CD_2Cl_2 and CD_3OD at 298 K with a Bruker AMX-400 spectrometer at 400.16 MHz with residual protic solvent as the internal reference [$CDCl_3$, δ= 7.26 ppm, C_6D_6, δ= 7.16 ppm; CD_2Cl_2, δ= 5.32 ppm; CD_3OD = 3.31 ppm]; chemical shifts (δ) are given in parts per million (ppm) and coupling constants (*J*) are given in Hertz (Hz). The proton spectra are reported as follows: δ (multiplicity, coupling constant *J*, number of protons). ^{13}C-NMR spectra were recorded in $CDCl_3$, C_6D_6, CD_2Cl_2 and CD_3OD at 298 K with the same spectrometer operating at 100.63 MHz with the central peak of $CDCl_3$ (δ = 77.26 ppm), C_6D_6 (δ = 128.06 ppm), CD_2Cl_2 (δ = 53.84 ppm) and CD_3OD (δ = 49.0 ppm) as the internal reference. DEPT-135 pulse sequences and HSQC bidimensional NMR spectra were used to aid in the assignment of signals in the ^{13}C- and ^{1}H-NMR spectra. Structural assignments were made with additional information from gCOSY, gHSQC, and gHMBC experiments. NOE-1D experiments were also performed in selected cases. UV/Vis spectra were recorded on a Cary 100 Bio spectrophotometer. Infrared spectra (IR) were obtained on a JASCO FT/IR-4200 infrared spectrometer, from a thin film deposited onto a NaCl glass. IR data include only characteristic absorptions. Peaks are quoted in wave numbers (cm^{-1}), and their relative intensities are reported as follows: s = strong, m = medium, w = weak.

2. Optimization of the olefin metathesis reaction

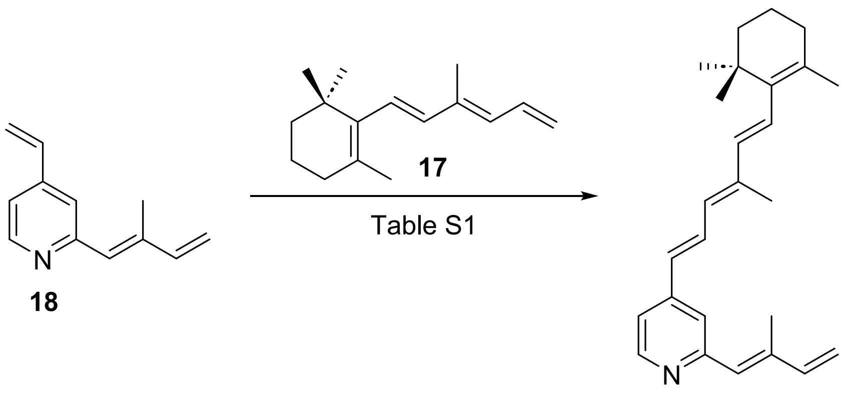

Table S1. Optimization of the olefin metathesis reaction.

Entry	Reaction conditions	Yield of 28 (%)
**1**	HG(II) (**26**), $BF_3{\cdot}OEt_2$, DCM, 50 ºC, 18h	**-**
**2**	HG(II) (**26**), $BF_3{\cdot}OEt_2$, DCM, 50 ºC, 18h	**18**
**3**	HG(II) (**26**), $BF_3{\cdot}OEt_2$, DCM, 60 ºC, 18h	**32**
**4**	HG(II) (**26**), $BF_3{\cdot}OEt_2$, DCM, 60 ºC, 18h	**7**
**5**	HG(II) (**26**), $BF_3{\cdot}OEt_2$, DCM, 60 ºC, 18h	**21**
**6**	HG(II) (**26**), $BF_3{\cdot}OEt_2$, DCM, 60 ºC, 23h	**-**
**7**	HG(II) (**26**), $BF_3{\cdot}OEt_2$, DCM, 60 ºC, 18h	**10**
**8**	HG(II) (**26**), $BF_3{\cdot}OEt_2$, DCM, 60 ºC, 72h	**-**
**9**	HG(II) (**26**), $BF_3{\cdot}OEt_2$, DCM, 60 ºC, 25h	**-**
**10**	HG(II) (**26**), $BF_3{\cdot}OEt_2$, DCM, 60 ºC, 21h	**-**
**11**	HG(II) (**26**), $BF_3{\cdot}OEt_2$, DCM, 60 ºC, 22h	**-**
**12**	HG(II) (**26**), $BF_3{\cdot}OEt_2$, NaOH, DCE, 60 ºC, 18h	**71**
**13**	Nitro-Grela (**27**), $BF_3{\cdot}OEt_2$, NaOH, DCE, 60 ºC, 19h	**43**

3. **Experimental procedures**

2-(2-Methylprop-1-en-1-yl)isonicotinaldehyde (22).

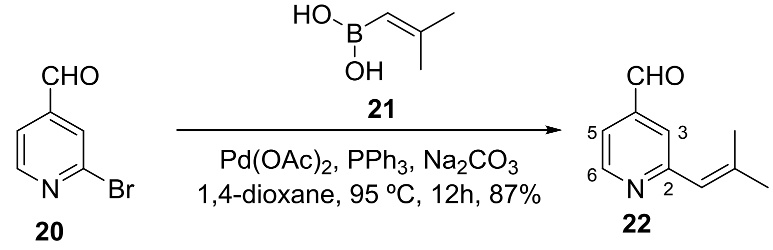

In a sealing tube, 2-bromoisonicotinaldehyde **20** (0.5 g, 2.69 mmol) and 2-(methylprop-1-en-1-yl)boronic acid **21** (0.54 g, 5.54 mmol) were dissolved in 1,4-dioxane (25 mL). Palladium acetate (0.060 g, 0.27 mmol), triphenylphosphine (0.18 g, 0.67 mmol) and Na_2CO_3 (2.7 mL, 2M, 5.38 mmol) were then added. The mixture was backfilled with argon and was heated at 95 °C for 12h. Water was added and the mixture was extracted with CH_2Cl_2 (3x). The combined organic layers were dried over Na_2SO_4, filtered and the solvent was evaporated. The residue was purified by flash column chromatography (silica gel, 98:2 *v*/*v* *n*-hexane/Et_3N; then, from 100:0 to 80:20 *v*/*v* *n*-hexane/EtOAc) to afford 0.38 g (87% yield) of a yellow oil, which was identified as 2-(2-methylprop-1-en-1-yl)isonicotinaldehyde **22**. The spectroscopic data of the obtained compound matched those for the same product previously reported in the literature.[1] **^{1}H-NMR** (400.16 MHz, $CDCl_3$): δ 10.05 (s, 1H, CHO), 8.81 (d, *J* = 5.0 Hz, 1H, H_6), 7.54 (s, 1H, H_3), 7.45 (dd, *J* = 5.0, 1.6 Hz, 1H, H_5), 6.41 (s, 1H, C<u>H</u>=CCH$_3$)$_2$), 2.12 (s, 3H, CH_3), 1.99 (s, 3H, CH_3) ppm. **^{13}C{^{1}H}-NMR** (100.63 MHz, $CDCl_3$): δ 191.9, 159.1, 150.4, 142.9, 141.7, 124.2, 122.4, 118.6, 27.6, 19.9 ppm.

(*E*)-2-(2-Methyl-3-oxoprop-1-en-1-yl)isonicotinaldehyde (12).

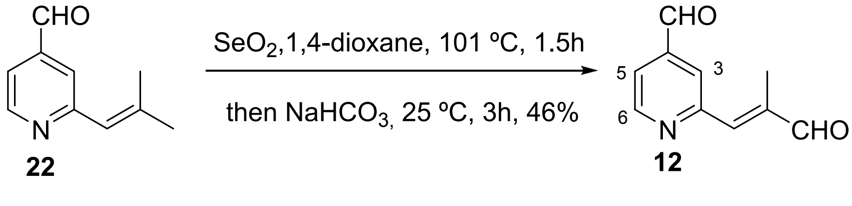

In a sealing tube containing a solution of 2-(2-methylprop-1-en-1-yl)isonicotinaldehyde **22** (0.1 g, 0.62 mmol) in 1,4-dioxane (5 mL), SeO_2 (0.08 g, 0.74 mmol) was added. The mixture was stirred at 101 °C for 1.5h. $NaHCO_3$ (0.12 g, 1.36 mmol) was added and the mixture was stirred for 3h at room temperature. The mixture was filtered through

Celite® and the solvent was evaporated. The residue was purified by flash column chromatography (silica gel, 98:2 *v/v* *n*-hexane/Et_3N; then, from 100:0 to 80:20 *v/v* *n*-hexane/EtOAc) to afford 0.05 g (46% yield) of a pink solid, which was identified as (*E*)-2-(2-methyl-3-oxoprop-1-en-1-yl)isonicotinaldehyde **12**. The spectroscopic data of the obtained compound matched those for the same product previously reported in the literature.[1] **^{1}H-NMR** (400.16 MHz, $CDCl_3$): δ 10.13 (s, 1H, CHO), 9.68 (s, 1H, CHO), 9.00 (d, *J* = 4.9 Hz, 1H, H_6), 7.88 (s, 1H, $H_{1'}$), 7.67 (dd, *J* = 4.9, 1.4 Hz, 1H, H_5), 7.33 (d, *J* = 1.4 Hz, 1H, H_3), 2.29 (d, *J* = 1.5 Hz, 3H, CH_3) ppm. **^{13}C{^{1}H}-NMR** (100.63 MHz, $CDCl_3$): δ 195.4, 191.1, 156.1, 151.4, 145.7, 142.7, 142.1, 123.9, 121.7, 11.1 ppm.

2-(2-Methylprop-1-en-1-yl)-4-vinylpyridine (23).

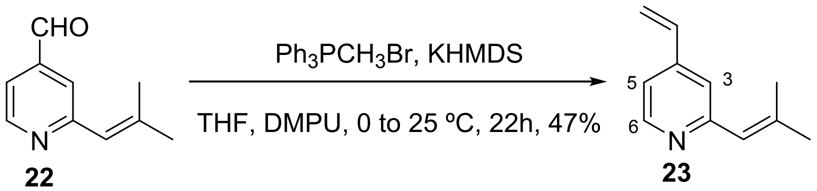

To a cooled (0 °C) suspension of methyltriphenylphosphonium bromide (0.24 g, 0.68 mmol) in THF (8 mL) and DMPU (1 mL) was added dropwise KHMDS (1.4 mL, 0.5 M in toluene, 0.68 mmol) and the mixture was stirred for 30 min. Then, 2-(2-methylprop-1-en-1-yl)isonicotinaldehyde **22** (0.1 g, 0.62 mmol) was added and the reaction mixture was stirred at 25 °C for 22h. Then, water was added and the reaction mixture was extracted with EtOAc (3x). The combined organic layers were washed with brine, dried over Na_2SO_4, filtered and the solvent was evaporated. The residue was purified by flash column chromatography (silica gel, 98:2 *v/v* *n*-hexane/Et_3N; then, from 95:5 to 80:20 *v/v* *n*-hexane/EtOAc) to afford 0.047 g (47% yield) of a white solid, which was identified as 2-(2-methylprop-1-en-1-yl)-4-vinylpyridine **23**. **^{1}H-NMR** (400.16 MHz, $CDCl_3$): δ 8.51 (d, *J* = 5.1 Hz, 1H, H_6), 7.14 (s, 1H, H_3), 7.07 (d, *J* = 5.2 Hz, 1H, H_5), 6.65 (dd, *J* = 17.6, 10.9 Hz, 1H, <u>CH</u>=CH_2), 6.33 (s, 1H, H_3), 5.94 (d, *J* = 17.6 Hz, 1H, =CH), 5.45 (d, *J* = 10.9 Hz, 1H, =CH), 2.07 (s, 3H, CH_3), 1.95 (s, 3H, CH_3) ppm. **^{13}C{^{1}H}-NMR** (100.63 MHz, $CDCl_3$): δ 157.9, 149.8, 144.6, 140.7, 135.2, 125.0, 121.0, 118.1, 117.5, 27.4, 19.8 ppm. **IR** (NaCl): υ 3088 (w, C-H), 2972 (w, C-H), 2927 (w, C-H), 2910 (w, C-H), 1655 (s), 1591 (s), 1543 (s) cm^{-1}. **HRMS** (ESI^+): calcd. for $C_{11}H_{14}N$ ($[M+H]^+$), 160.1121; found, 160.1120.

(*E*)-2-Methyl-3-(4-vinylpyridin-2-yl)acrylaldehyde (24).

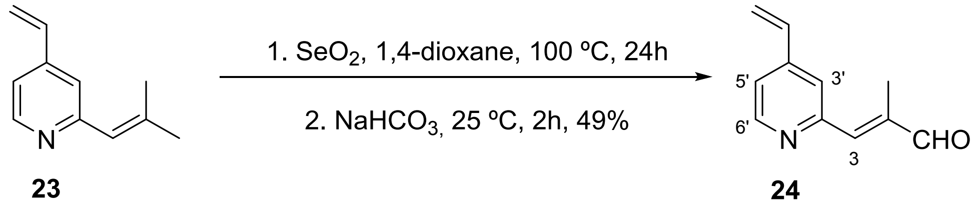

To a solution of 2-(2-methylprop-1-en-1-yl)-4-vinylpyridine **23** (32.3 mg, 0.20 mmol) in 1,4-dioxane (2 mL) was added SeO_2 (38.3 mg, 0.35 mmol) and the resulting mixture was heated to reflux for 24h. After cooling down to room temperature, $NaHCO_3$ (85.2 mg, 1.01 mmol) was added and the mixture was stirred for 2h, and then filtered off through $NaHCO_3$ and Celite®. The residue was purified by flash column chromatography (silica gel, 98:2 *v*/*v* *n*-hexane/Et_3N; then, from 90:10 to 60:40 *v*/*v* *n*-hexane/EtOAc) to afford 17.2 mg (49% yield) of a dark solid, which was identified as (*E*)-2-methyl-3-(4-vinylpyridin-2-yl)acrylaldehyde **24**. **^{1}H-NMR** (400.16 MHz, $CDCl_3$): δ 9.64 (s, 1H, CHO), 8.68 (d, *J* = 5.1 Hz, 1H, $H_{6'}$), 7.47 (s, 1H, H_3), 7.28 - 7.24 (m, 2H, $H_{3'}$ + $H_{5'}$), 6.70 (dd, *J* = 17.6, 10.9 Hz, 1H, <u>CH</u>=CH_2), 6.01 (d, *J* = 17.6 Hz, 1H, =CH), 5.55 (d, *J* = 10.9 Hz, 1H, =CH), 2.25 (s, 3H, CH_3) ppm. **^{13}C{^{1}H}-NMR** (100.63 MHz, $CDCl_3$): δ 196.0, 155.0, 150.4, 147.7, 145.5, 141.7, 134.5, 123.3, 120.4, 119.5, 11.2 ppm. **IR** (NaCl): υ 2924 (w, C-H), 1684 (s, C=O), 1630 (w), 1590 (w) cm^{-1}. **HRMS** (ESI^+): calcd. for $C_{11}H_{12}NO$ ($[M+H]^+$), 174.0913; found, 174.0911.

(*E*)-2-(2-Methyl-3-oxoprop-1-en-1-yl)isonicotinaldehyde (12).

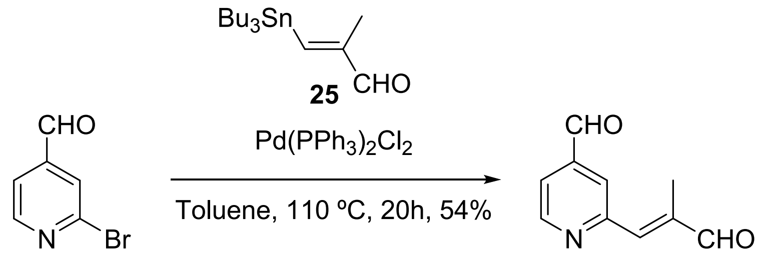

To a solution of 2-bromoisonicotinaldehyde **20** (50 mg, 0.27 mmol) in toluene (1 mL), $Pd(PPh_3)_2Cl_2$ (8 mg, 0.01 mmol) and *(E)*-2-methyl-3-(tributylstannyl)acrylaldehyde **25** (145 mg, 0.40 mmol) were added. The mixture was thoroughly degassed (with three Argon-cooling cycles) and stirred at 110 °C for 20 h. A saturated aqueous solution of

NH_4Cl was added and the mixture was extracted with EtOAc (3x). The combined organic layers were washed with a 1M aqueous solution of KF (3x), dried over Na_2SO_4, filtered and the solvent was evaporated. The residue was purified by flash column chromatography (silica gel, 98:2 *v/v* *n*-hexane/Et_3N; then, from 90:10 to 50:50 *v/v* *n*-hexane/EtOAc) to afford 25 mg (54%) of an pink solid, which was identified as *(E)*-2-(2-methyl-3-oxoprop-1-en-1-yl)isonicotinaldehyde **12**. The spectroscopic data is listed above, following the alternative procedure.

(*E*)-2-(2-Methylbuta-1,3-dien-1-yl)-4-vinylpyridine (18).

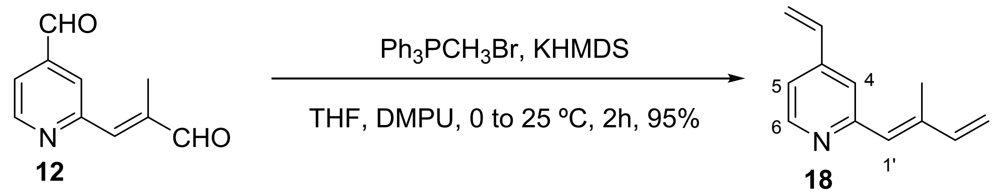

To a cooled (0 °C) solution of methyltriphenylphosphonium bromide (0.54 g, 1.50 mmol) in THF (9 mL) and DMPU (1 mL), KHMDS (3.0 mL, 0.5 M in toluene, 1.50 mmol) was added and the resulting mixture was stirred for 30 min. After that, a solution of (*E*)-2-(2-methyl-3-oxoprop-1-en-1-yl)isonicotinaldehyde **12** (0.12 g, 0.69 mmol) in THF (1 mL) was added dropwise and the mixture was stirred at 25 °C for 2h. A saturated aqueous solution of NH_4Cl was added and the mixture was extracted with EtOAc (3x). The combined organic layers were washed with brine, dried over Na_2SO_4, filtered and the solvent was evaporated. The residue was purified by flash column chromatography (silica gel, 98:2 *v/v* *n*-hexane/Et_3N; then, from 100:0 to 95:5 *v/v* *n*-hexane/EtOAc) to afford 0.11 g (95% yield) of a yellow oil, which was identified as (*E*)-2-(2-methylbuta-1,3-dien-1-yl)-4-vinylpyridine **18**. **^{1}H-NMR** (400.16 MHz, C_6D_6): δ 8.50 (d, *J* = 5.1 Hz, 1H, H_6), 6.86 (s, 1H, $H_{1'}$), 6.61 (dd, *J* = 5.1, 1.7 Hz, 1H, H_5), 6.54 (ddd, *J* = 17.3, 10.6, 0.8 Hz, 1H, <u>CH</u>=CH_2), 6.46 (s, 1H, H_3), 6.27 (dd, *J* = 17.6, 10.8 Hz, 1H, <u>CH</u>=CH_2), 5.57 (dd, *J* = 17.6, 0.8 Hz, 1H, =CH), 5.35 (d, *J* = 17.3 Hz, 1H, =CH), 5.12 (d, *J* = 10.6 Hz, 1H, =CH), 5.08 (d, *J* = 10.8, 0.8 Hz, 1H, =CH), 2.49 (d, *J* = 1.3 Hz, 3H, CH_3) ppm. **^{13}C{^{1}H}-NMR** (100.63 MHz, C_6D_6): δ 158.1, 149.9, 144.6, 142.7, 140.7, 135.4, 130.9, 122.7, 117.9, 117.7, 114.6, 13.6 ppm. **IR** (NaCl): υ 3088 (w, C-H), 3006 (w, C-H), 2917 (w, C-H), 1606 (s, C=C), 1589 (m), 1542 (m) cm^{-1}. **HRMS** (ESI$^+$): calcd. for $C_{12}H_{14}N$ ($[M + H]^+$), 172.1121; found, 172.1123.

(*E*)-2-(2-Methylbuta-1,3-dien-1-yl)-4-vinylpyridine (18).

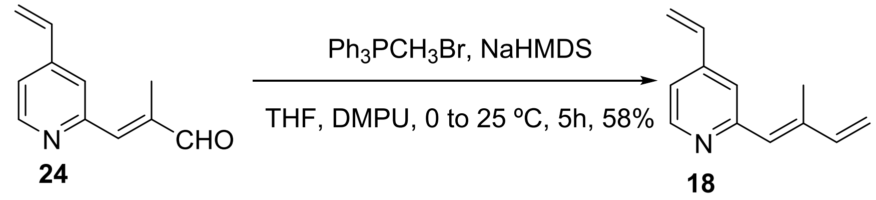

To a cooled (0 °C) suspension of methyltriphenylphosphonium bromide (60.1 mg, 0.16 mmol) in THF (2 mL) and DMPU (0.2 mL) was added dropwise NaHMDS (0.16 mL, 1 M in THF, 0.16 mmol) and the mixture was stirred for 30 min. Then, (*E*)-2-methyl-3-(4-vinylpyridin-2-yl)acrylaldehyde **24** (23.8 mg, 0.14 mmol) was added and the reaction mixture was stirred at 25 °C for 5h. Then, water was added and the reaction mixture was extracted with EtOAc (3x). The combined organic layers were washed with brine, dried over Na_2SO_4, filtered and the solvent was evaporated. The residue was purified by flash column chromatography (silica gel, 98:2 *v*/*v* *n*-hexane/Et_3N; then, from 95:5 to 90:10 *v*/*v* *n*-hexane/EtOAc) to afford 13.6 mg (58% yield) of a yellow oil, which was identified as (*E*)-2-(2-methylbuta-1,3-dien-1-yl)-4-vinylpyridine **18**. The spectroscopic data is listed above, following the alternative procedure.

Compound 28.

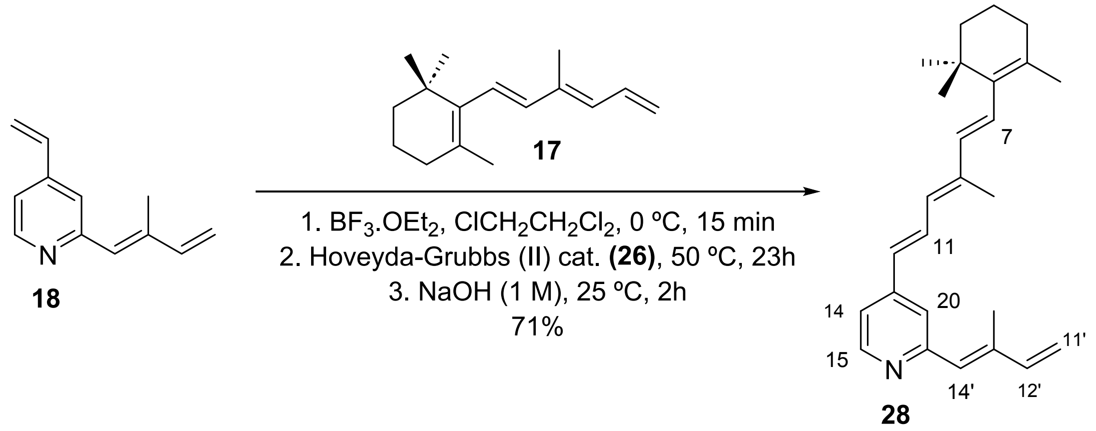

To a cooled (0 °C) stirred solution of (*E*)-2-(2-methylbuta-1,3-dien-1-yl)-4-vinylpyridine **18** (20 mg, 0.12 mmol) in 1,2-dichloroethane (2 mL), $BF_3{\cdot}OEt_2$ (0.02 mL, 0.13 mmol) was added dropwise and the mixture was stirred at 0 °C for 15 min. Then, 2nd generation Hoveyda-Grubbs' catalyst **26** (7.3 mg, 0.01 mmol) and 1,3,3-trimethyl-2-((1*E*,3*E*)-3-methylhexa-1,3,5-trien-1-yl)cyclohex-1-ene (**17**) [2,3] (126.3 mg, 0.58 mmol) were added

and the resulting mixture was stirred at 50 °C for 23h. NaOH (0.24 mL, 1 M in water, 0.24 mmol) was then added and the solution was stirred for 2h at 25 °C. Finally, the mixture was filtered through Celite® and the solvent was evaporated. The residue was purified by flash-column chromatography (C18 silica gel, from 50:50 to 100:0 *v*/*v* CH_3CN/H_2O) to afford 30 mg (71% yield) of a dark orange oil, which was identified as compound **28**. **^{1}H-NMR** (400.16 MHz, CD_2Cl_2): δ 8.48 (d, *J* = 5.1 Hz, 1H, H_{15}), 7.38 (dd, *J* = 15.4, 11.4 Hz, 1H, H_{11}), 7.21 (s, 1H, H_{20}), 7.11 (d, *J* = 5.2 Hz, 1H, H_{14}), 6.57 (dd, *J* = 17.3, 10.7 Hz, 1H, $H_{12'}$), 6.50-6.43 (m, 2H, $H_{14'}$ + H_{12}), 6.30 (d, *J* = 16.1 Hz, 1H, H_7), 6.22 (d, *J* = 11.4 Hz, 1H, H_{10}), 6.16 (d, *J* = 16.1 Hz, 1H, H_8), 5.42 (d, *J* = 17.3 Hz, 1H, $H_{11'}$), 5.20 (d, *J* = 10.7 Hz, 1H, $H_{11'}$), 2.26 (s, 3H, CH_3), 2.07-1.97 (m, 5H, CH_3 + CH_2), 1.71 (s, 3H, CH_3), 1.66-1.56 (m, 2H, CH_2) 1.50-1.44 (m, 2H, CH_2), 1.02 (s, 6H, 2xCH_3) ppm. **^{13}C{^{1}H}-NMR** (100.63 MHz, CD_2Cl_2): δ 157.0, 149.2, 144.9, 141.8, 139.6, 139.3, 137.4, 137.0, 130.3, 129.7, 129.3, 128.9, 128.7, 128.5, 121.7, 117.5, 114.1, 39.4, 34.0, 32.8, 28.5 (2x), 21.2, 19.0, 12.7, 12.4 ppm. **HRMS** (ESI$^+$): Calcd for $C_{26}H_{34}N$ ([M + H]$^+$), 360.2686; found, 360.2686. **UV** (MeOH): λ_{max} 347 nm.

A2E (6).

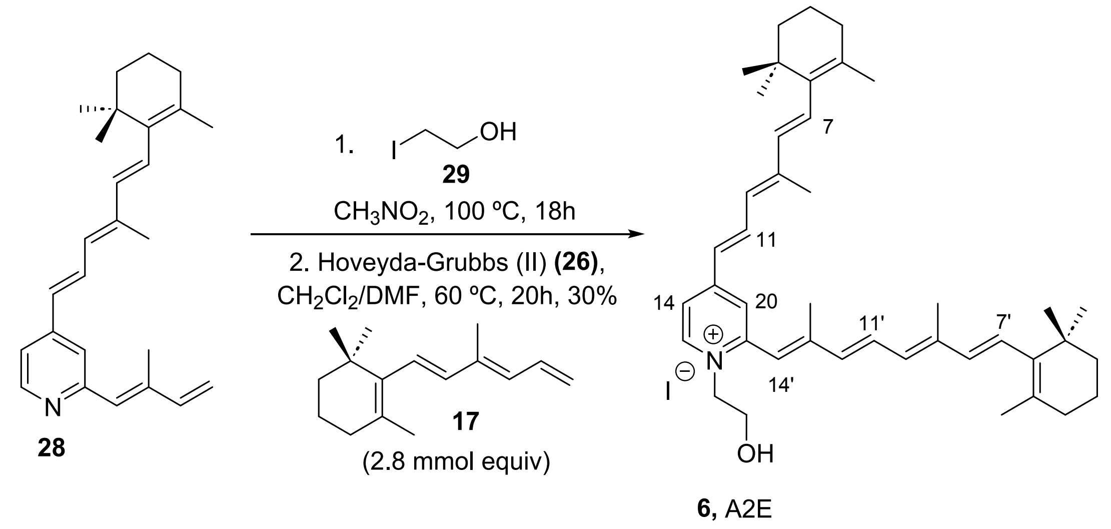

To a solution of **28** (14 mg, 0.04 mmol) in CH_3NO_2 (0.7 mL), 2-iodoethanol **29** (0.03 mL, 0.39 mmol) was added. The resulting solution was heated at 100 °C for 18h. The solvent was evaporated under vacuum, the residue was triturated with *n*-hexane and Et_2O mixtures and the solvents were removed.

Over a solution of the crude obtained above (19.7 mg, 0.04 mmol) in CH_2Cl_2 (0.7 mL), 2 drops of DMF, 2nd generation Hoveyda-Grubbs' catalyst **26** (2.3 mg, 0.004 mmol) and 1,3,3-trimethyl-2-((1*E*,3*E*)-3-methylhexa-1,3,5-trien-1-yl)cyclohex-1-ene **17** (24.1 mg, 0.11 mmol) were added and the resulting mixture was stirred at 60 °C for 20h. Finally, the mixture was filtered through Celite® and the solvent was evaporated. The residue was purified by flash-column chromatography (Grade IV alumina, from 100:0 to 90:10 *v/v* CH_2Cl_2/CH_3OH) to afford 7.1 mg (30% yield) of a red syrup, which was identified as A2E (**6**). The spectroscopic data of the obtained compound matched those for the same product previously reported in the literature.[1] **^{1}H-NMR** (400.16 MHz, CD_3OD): δ 8.56 (d, *J* = 6.9 Hz, 1H, H_{15}), 8.01 (dd, *J* = 15.2, 11.7 Hz, 1H, H_{11}), 7.96 (d, *J* = 6.9 Hz, 1H, H_{14}), 7.87 (s, 1H, H_{20}), 7.12 (dd, *J* = 15.1, 11.4 Hz, 1H, $H_{11'}$), 6.78 (d, *J* = 15.2 Hz, 1H, H_{12}), 6.72 (s, 1H, $H_{14'}$), 6.64 (d, *J* = 15.1 Hz, 1H, $H_{12'}$), 6.54 (d, *J* = 16.0 Hz, 1H, H_7), 6.42 (d, *J* = 11.7 Hz, 1H, H_{10}), 6.34 (d, *J* = 16.0 Hz, 1H, $H_{7'}$), 6.28 (d, *J* = 16.0 Hz, 1H, H_8), 6.25 (d, *J* = 11.4 Hz, 1H, $H_{10'}$), 6.17 (d, *J* = 16.0 Hz, 1H, $H_{8'}$), 4.56 (t, *J* = 5.0 Hz, 2H, CH_2), 3.93 (t, *J* = 5.0 Hz, 2H, CH_2), 2.18 (s, 3H, CH_3), 2.16 (d, *J* = 1.0 Hz, 3H, CH_3), 2.11 – 2.02 (m, 7H, CH_2 + CH_3), 1.75 (s, 3H, CH_3), 1.73 (s, 3H, CH_3), 1.69 – 1.63 (m, 4H, 2xCH_2), 1.53 – 1.48 (m, 4H, 2xCH_2), 1.06 (s, 6H, 2xCH_3), 1.05 (s, 6H, 2xCH_3) ppm.

(*E*)-2-(3-((*tert*-Butyldimethylsilyl)oxy)-2-methylprop-1-en-1-yl)isonicotinaldehyde (32).

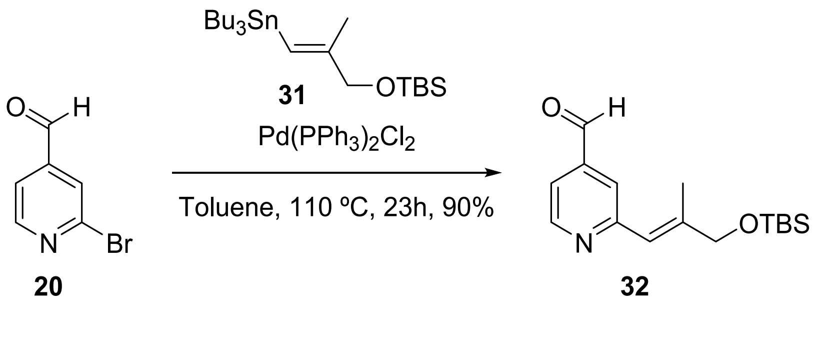

To a degassed solution of 2-bromoisonicotinaldehyde **20** (0.3 g, 1.61 mmol) and (*E*)-*tert*-butyldimethyl((2-methyl-3-(tributylstannyl)allyl)oxy)silane **31** (1.15 g, 2.42 mmol) in toluene (10 mL), $Pd(PPh_3)_2Cl_2$ (45.3 mg, 0.06 mmol) was added. The mixture was stirred at 110 °C for 23h. A saturated aqueous solution of NH_4Cl was added and the mixture was extracted with EtOAc (3x). The combined organic layers were dried over Na_2SO_4, filtered and the solvent was evaporated. The residue was purified by flash column

chromatography (silica gel, 98:2 *v/v* *n*-hexane/Et_3N; then, from 100:0 to 90:10 *v/v* n-hexane/EtOAc) to afford 0.42 g (90% yield) of a colourless oil, which was identified as **32**. The spectroscopic data of the obtained compound matched those for the same product previously reported in the literature.[4] **^{1}H-NMR** (400.16 MHz, C_6D_6): δ 9.33 (s, 1H, CHO), 8.51 (d, *J* = 4.8 Hz, 1H, H_6), 7.18 (s, 1H, H_3), 6.87 (d, *J* = 4.8 Hz, 1H, H_5), 6.81 (s, 1H, $H_{1'}$), 4.07 (s, 2H, 2x$H_{3'}$), 2.17 (s, 3H, CH_3), 1.02 (s, 9H, SiC($\underline{CH_3}$)$_3$), 0.10 (s, 6H, 2xSi-CH_3) ppm. **^{13}C{^{1}H}-NMR** (100.63 MHz, C_6D_6): δ 191.2, 159.2, 150.5, 144.6, 142.1, 123.1, 122.1, 118.4, 68.4, 26.1 (3x), 18.6, 15.8, -5.2 (2x) ppm.

(*E*)-2-(3-((*tert*-Butyldimethylsilyl)oxy)-2-methylprop-1-en-1-yl)isonicotinaldehyde (32).

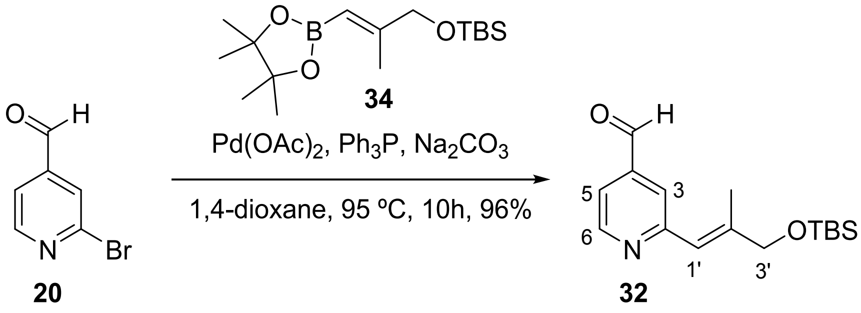

$Pd(OAc)_2$ (24.1 mg, 0.11 mmol), PPh_3 (70.5 mg, 0.27 mmol) and 2-bromoisonicotinaldehyde **20** (0.2 g, 1.07 mmol) were added to a sealing tube. Then, compound **34** (0.67 g, 2.15 mmol) in 1,4-dioxane (10 mL) and Na_2CO_3 (1.1 mL, 2M, 2.2 mmol) were added and the tube was sealed. The reaction mixture was stirred at 95 °C for 10h. After cooling down to room temperature, the mixture was extracted with CH_2Cl_2 (3x). The combined organic layers were dried over Na_2SO_4, filtered and the solvent was evaporated. The residue was purified by flash column chromatography (silica gel, 98:2 *v/v* *n*-hexane/Et_3N; then, from 100:0 to 90:10 *v/v* *n*-hexane/EtOAc) to afford 0.30 g (96% yield) of a white solid, which was identified as (*E*)-2-(3-((*tert*-butyldimethylsilyl)oxy)-2-methylprop-1-en-1-yl)isonicotinaldehyde **32**.

(*E*)-2-(3-((*tert*-Butyldimethylsilyl)oxy)-2-methylprop-1-en-1-yl)-4-vinylpyridine (33).

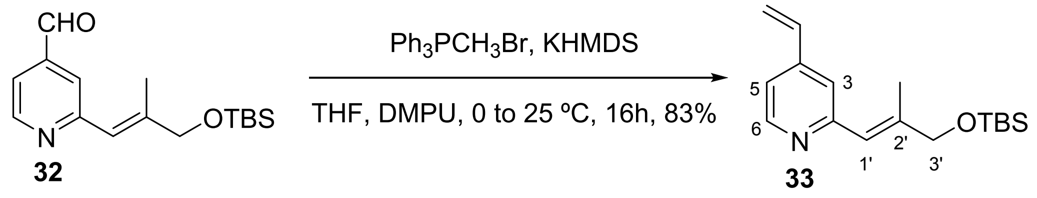

To a cooled (0 °C) solution of methyltriphenylphosphonium bromide (0.29 g, 0.82 mmol) in THF (9 mL) and DMPU (1 mL), KHMDS (1.64 mL, 0.5 M in toluene, 0.82 mmol) was added and the resulting mixture was stirred for 30 min. After that, a solution of (*E*)-2-(3-((*tert*-butyldimethylsilyl)oxy)-2-methylprop-1-en-1-yl)isonicotinaldehyde **32** (0.2 g, 0.69 mmol) in THF (1 mL) was added dropwise and the mixture was stirred at 25 °C for 16h. A saturated aqueous solution of NH_4Cl was added and the mixture was extracted with EtOAc (3x). The combined organic layers were washed with brine, dried over Na_2SO_4, filtered and the solvent was evaporated. The residue was purified by flash column chromatography (silica gel, 98:2 *v/v* *n*-hexane/Et_3N; then, from 100:0 to 90:10 *v/v* *n*-hexane/EtOAc) to afford 0.16 g (83%) of a yellow oil, which was identified as (*E*)-2-(3-((*tert*-butyldimethylsilyl)oxy)-2-methylprop-1-en-1-yl)-4-vinylpyridine **33**. **^{1}H-NMR** (400.16 MHz, C_6D_6): δ 8.53 (d, *J* = 5.2 Hz, 1H, H_6), 7.03 (s, 1H, $H_{1'}$), 6.85 (d, *J* = 1.7 Hz, 1H, H_3), 6.65 (dd, *J* = 5.1, 1.7 Hz, 1H, H_5), 6.27 (dd, *J* = 17.6, 10.8 Hz, 1H, $\underline{CH}=CH_2$), 5.55 (d, *J* = 17.6 Hz, 1H, =CH), 5.05 (d, *J* = 10.8 Hz, 1H, =CH), 4.11 (d, *J* = 1.8 Hz, 2H, $2xH_{3'}$), 2.28 (s, 3H, CH_3), 1.02 (s, 9H, $SiC(\underline{CH_3})_3$), 0.10 (s, 6H, 2xSi-CH_3) ppm. **^{13}C{^{1}H}-NMR** (100.63 MHz, C_6D_6): δ 158.4, 149.9, 144.7, 142.9, 135.5, 123.1, 121.9, 117.7, 117.5, 68.7, 26.2 (3x), 18.6, 15.9, -5.2 (2x) ppm. **IR** (NaCl): υ 2954 (s, C-H), 2928 (s, C-H), 2856 (s, C-H), 1664 (m), 1471 (w, C=C), 1254 (m, C-O), 1112 (s, Si-O-C) cm^{-1}. **HRMS** (ESI$^+$): calcd. for $C_{17}H_{28}NOSi$ ([M+H]$^+$), 290.1940; found, 290.1938.

Compound 37.

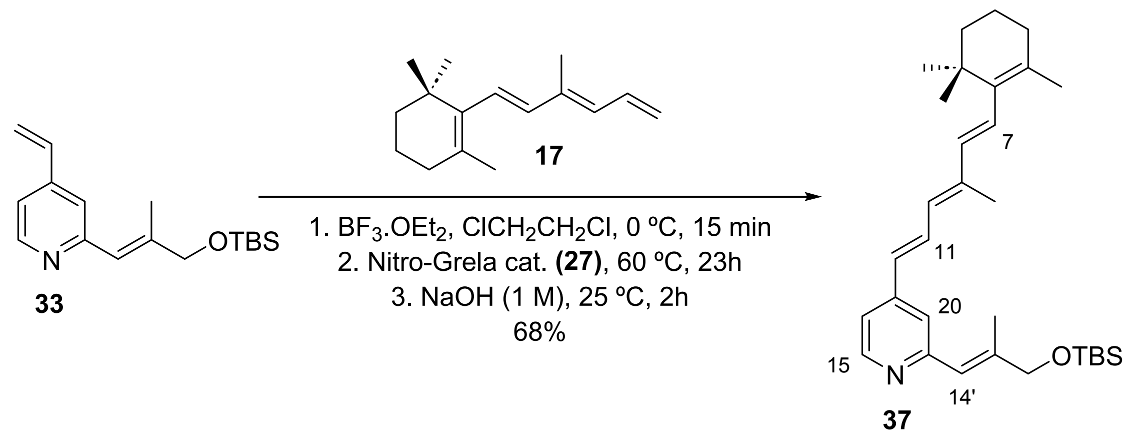

To a cooled (0 °C) stirred solution of (*E*)-2-(3-((*tert*-butyldimethylsilyl)oxy)-2-methylprop-1-en-1-yl)-4-vinylpyridine **33** (20 mg, 0.07 mmol) in 1,2-dichloroethane (1 mL), $BF_3 \cdot OEt_2$ (0.010 mL, 0.08 mmol) was added dropwise and the mixture was stirred at 0 °C for 15 min. Then, Nitro-Grela's catalyst **27** (4.9 mg, 0.007 mmol) and 1,3,3-trimethyl-2-((1*E*,3*E*)-3-methylhexa-1,3,5-trien-1-yl)cyclohex-1-ene **17** (44.8 mg, 0.21 mmol) were added and the resulting mixture was stirred at 60 °C for 23h. NaOH (0.14 mL, 1 M in water, 0.14 mmol) was then added and the solution was stirred for 2h at 25 °C. Finally, the mixture was filtered through Celite® and the solvent was evaporated. The residue was purified by flash-column chromatography (C18 silica gel, from 50:50 to 100:0 *v*/*v* CH_3CN/H_2O) to afford 22.3 mg (68% yield) of a dark yellow oil, which was identified as compound **37**. The spectroscopic data of the obtained compound matched those for the same product previously reported in the literature.[4] **^{1}H-NMR** (400.16 MHz, C_6D_6): δ 8.58 (d, *J* = 5.1 Hz, 1H, H_{15}), 7.22 (dd, *J* = 15.3, 11.3 Hz, 1H, H_{11}), 7.12 (s, 1H, H_{20}), 6.87 (s, 1H, $H_{14'}$), 6.75 (d, *J* = 5.1 Hz, 1H, H_{14}), 6.37 (d, *J* = 16.3 Hz, 1H, H_8), 6.31 (d, *J* = 16.3 Hz, 1H, H_7), 6.23 (d, *J* = 15.6 Hz, 1H, H_{12}), 6.17 (d, *J* = 12.3 Hz, 1H, H_{10}), 4.15 (s, 2H, $2H_{12'}$), 2.34 (s, 3H, CH_3), 1.97 (t, *J* = 6.1 Hz, 2H, CH_2), 1.84 (s, 3H, CH_3), 1.79 (s, 3H, CH_3), 1.65 - 1.55 (m, 2H, CH_2), 1.51 - 1.46 (m, 2H, CH_2), 1.14 (s, 6H, $2xCH_3$), 1.03 (s, 9H, $SiC(\underline{CH_3})_3$), 0.11 (s, 6H, 2xSi-CH_3) ppm. **^{13}C{^{1}H}-NMR** (100.63 MHz, C_6D_6): δ 158.4, 149.9, 145.1, 142.9, 138.7, 138.2, 138.1, 130.4, 130.3, 129.9, 129.1, 128.5, 123.3, 122.0, 117.5, 68.8, 39.9, 34.6, 33.3, 29.2 (2x), 26.2 (3x), 22.0, 19.7, 18.7, 15.9, 12.9, -5.1 (2x) ppm.

Compound 37.

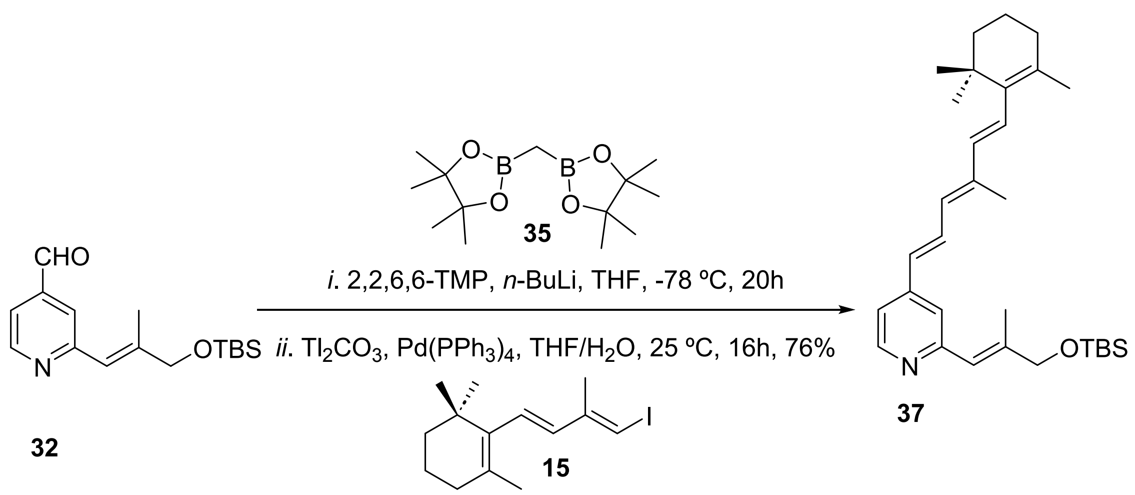

To a cooled (0 °C) solution of 2,2,6,6-tetramethylpiperidine (0.63 mL, 3.76 mmol) in THF (1 mL), *n*-BuLi (1.9 mL, 1.9 M in hexanes, 3.76 mmol) was added and the mixture was stirred for 30 min. Then, a solution of bis(pinacolatoboryl)methane **35** (1.0 g, 3.76 mmol) in THF (3 mL) was added and the reaction mixture was stirred for 10 min. The mixture was cooled down to -78 °C, a solution of **32** [4] (0.27 g, 0.94 mmol) in THF (3 mL) was added dropwise, and the reaction mixture was stirred at -78 °C for 20h. A saturated aqueous solution of NH_4Cl was added and the mixture was extracted with Et_2O (3x). The combined organic layers were washed with brine, dried over anhydrous Na_2SO_4, filtered and the solvent was evaporated.

To a cooled (0 °C) solution of 2-((1*E*,3*E*)-4-iodo-3-methylbuta-1,3-dien-1-yl)-1,3,3-trimethylcyclohex-1-ene **15** (68.3 mg, 0.22 mmol) and the crude obtained above (60 mg, 0.18 mmol) in a THF-H_2O mixture (6 mL, 4:1 *v*/*v*), Tl_2CO_3 (0.21 g, 0.45 mmol) and $Pd(PPh_3)_4$ (10.4 mg, 0.01 mmol) were added, and the resulting mixture was stirred at 25 °C for 16h. The mixture was filtered through Celite® and Na_2SO_4 washing with Et_2O, and the solvent was evaporated. The residue was purified by flash column chromatography (silica gel, 98:2 *v*/*v* *n*-hexane/Et_3N; then, from 100:0 to 90:10 *v*/*v* *n*-hexane/EtOAc) to afford 65.1 g (76% yield) of a dark yellow oil, which was identified as **37**. The spectroscopic data is listed above, following the alternative procedure.

Compound 38.

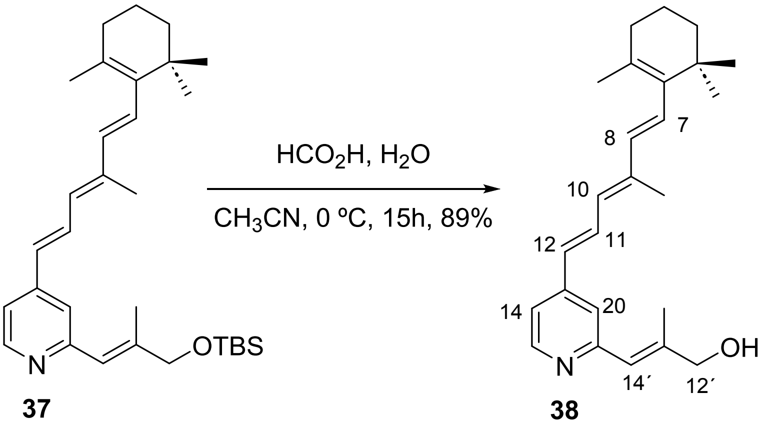

37 **38**

A cooled (0 °C) mixture of HCO_2H (1.5 mL, 38.68 mmol) and H_2O (0.48 mL, 26.50 mmol) was added to a cooled (0 °C) solution of **37** (53.6 mg, 0.11 mmol) in CH_3CN (3 mL). The mixture was stirred at 0 °C for 15h. After that, a saturated aqueous solution of $NaHCO_3$ was added and the mixture was extracted with EtOAc (3x). The combined organic layers were dried over Na_2SO_4, filtered and the solvent was evaporated. The residue was purified by flash column chromatography (silica gel, 98:2 (*v*/*v*) *n*-hexane/Et_3N; then, from 70:30 to 50:50 (*v*/*v*) *n*-hexane/EtOAc) to afford 36.2 mg (89% yield) of a dark orange oil, which was identified as **38**. The spectroscopic data of the obtained compound matched those for the same product previously reported in the literature.[4]
^{1}H-NMR (400.16 MHz, CD_2Cl_2): δ 8.47 (d, *J* = 5.3 Hz, 1H, H_{15}), 7.41 (dd, *J* = 15.4, 11.4 Hz, 1H, H_{11}), 7.24 (s, 1H, H_{20}), 7.14 (d, *J* = 5.2 Hz, 1H, H_{14}), 6.60 (s, 1H, $H_{14'}$), 6.49 (d, *J* = 15.4 Hz, 1H, H_{12}), 6.33 (d, *J* = 16.2 Hz, 1H, H_8), 6.25 (d, *J* = 11.4 Hz, 1H, H_{10}), 6.19 (d, *J* = 16.2 Hz, 1H, H_7), 4.18 (s, 2H, $H_{12'}$), 2.09 – 1.98 (m, 8H, CH_2 + 2xCH_3), 1.74 (s, 3H, CH_3), 1.68 – 1.61 (m, 2H, CH_2), 1.55 – 1.46 (m, 2H, CH_2), 1.06 (s, 6H, 2xCH_3) ppm. **^{13}C{^{1}H}-NMR** (100.63 MHz, CD_2Cl_2): δ 157.8, 149.6, 145.9, 143.7, 140.2, 138.3, 137.8, 130.6, 130.2, 129.7, 129.6, 129.4, 123.5, 121.8, 118.3, 68.5, 40.2, 34.8, 33.6, 29.3 (2x), 22.0, 19.8, 15.9, 13.2 ppm.

Compound 39.

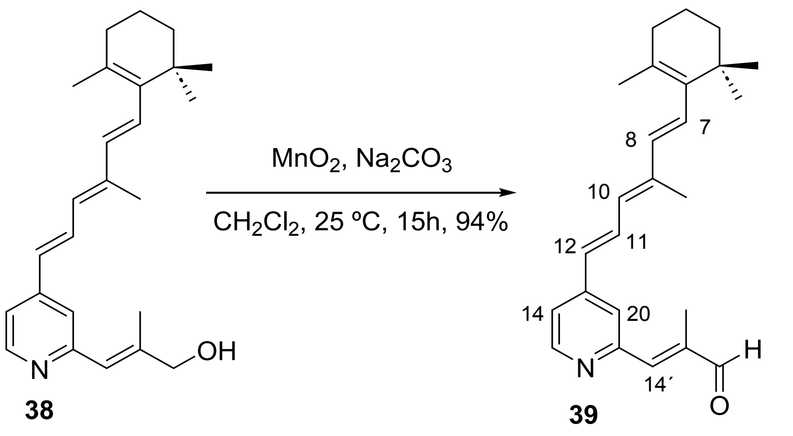

To a solution of **38** (78.0 mg, 0.22 mmol) in CH_2Cl_2 (6 mL) at room temperature, Na_2CO_3 (0.409 g, 3.86 mmol) and MnO_2 (0.336 g, 3.86 mmol) were added. The resulting reaction mixture was stirred at room temperature for 15h. The mixture was filtered through Celite® washing with CH_2Cl_2 to afford 0.073 g (94% yield) of a dark red oil, which was identified as **39**. The spectroscopic data of the obtained compound matched those for the same product previously reported in the literature.[4] **^{1}H-NMR** (400.16 MHz, C_6D_6): δ 9.49 (s, 1H, CHO), 8.46 (d, *J* = 5.1 Hz, 1H, H_{15}), 7.23 (dd, *J* = 15.4, 11.4 Hz, 1H, H_{11}), 6.94 (s, 1H, H_{20}), 6.75 (s, 1H, $H_{14'}$), 6.69 (d, *J* = 5.1, 1H, H_{14}), 6.42 (d, *J* = 16.1 Hz, 1H, H_8), 6.35 (d, *J* = 16.1 Hz, 1H, H_7), 6.22 (d, *J* = 11.4 Hz, 1H, H_{10}), 6.19 (d, *J* = 15.3 Hz, 1H, H_{12}), 2.50 (s, 3H, CH_3), 1.98 (t, *J* = 6.4 Hz, 2H, CH_2), 1.91 (s, 3H, CH_3), 1.81 (s, 3H, CH_3), 1.63 – 1.57 (m, 2H, CH_2), 1.52 – 1.47 (m, 2H, CH_2), 1.15 (s, 6H, 2xCH_3) ppm. **^{13}C{^{1}H}-NMR** (100.63 MHz, C_6D_6): δ 194.9, 155.7, 150.4, 146.6, 145.4, 142.2, 139.7, 138.1, 137.9, 130.2, 129.9 (2x), 129.3, 129.2, 123.7, 119.7, 39.9, 34.6, 33.4, 29.2 (2x), 22.0, 19.7, 12.9, 11.4 ppm.

Compound 11.

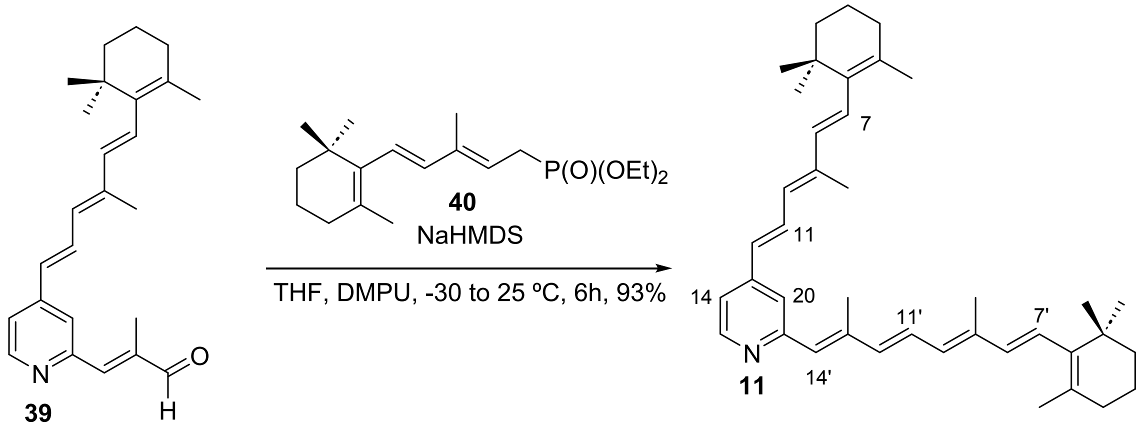

To a cooled (-30 °C) stirred solution of phosphonate **40** (47.2 mg, 0.14 mmol) in THF (0.4 mL), DMPU (0.2 mL) and NaHMDS (0.13 mL, 1M in THF, 0.13 mmol) were added. Subsequently, **39** (29.5 mg, 0.08 mmol) in THF (0.4 mL) was added and the mixture was allowed to warm up to room temperature for 6h. A saturated aqueous solution of NH_4Cl was added and the mixture was extracted with Et_2O (3x). The combined organic layers were washed with brine, dried over Na_2SO_4, filtered and the solvent was evaporated. The residue was purified by flash column chromatography (silica gel, 98:2 *v*/*v* *n*-hexane/Et_3N; then, from 100:0 to 90:10 *v*/*v* *n*-hexane/EtOAc) to afford 41.7 mg (93% yield) of a dark red foam, which was identified as **11**. The spectroscopic data of the obtained compound matched those for the same product previously reported in the literature.[1] **^{1}H-NMR** (400.16 MHz, C_6D_6): δ 8.69 (d, *J* = 5.2 Hz, 1H, H_{15}), 7.36 (dd, *J* = 15.3, 11.4 Hz, 1H, H_{11}), 7.13 (s, 1H, H_{20}), 7.08 (dd, *J* = 15.1, 11.4 Hz, 1H, $H_{11'}$), 6.82 (dd, *J* = 5.1, 1.6 Hz, 1H, H_{14}), 6.74 – 6.64 (m, 2H, H_{12} + $H_{14'}$), 6.55 – 6.39 (m, 6H, $H_{12'}$ + H_{10} + $H_{10'}$ + H_7 + $H_{7'}$ + $H_{8'}$), 6.36 (d, *J* = 16.0 Hz, 1H, H_8), 2.83 (d, *J* = 1.1 Hz, 3H, CH_3), 2.09 (t, *J* = 6.3 Hz, 4H, $2xCH_2$), 2.02 (d, *J* = 1.1 Hz, 3H, CH_3), 1.99 (d, *J* = 1.1 Hz, 3H, CH_3), 1.95 – 1.85 (m, 6H, $2xCH_3$), 1.74 – 1.69 (m, 4H, $2xCH_2$), 1.64 – 1.57 (m, 4H, $2xCH_2$), 1.25 (s, 12H, $4xCH_3$) ppm.

A2E (6).

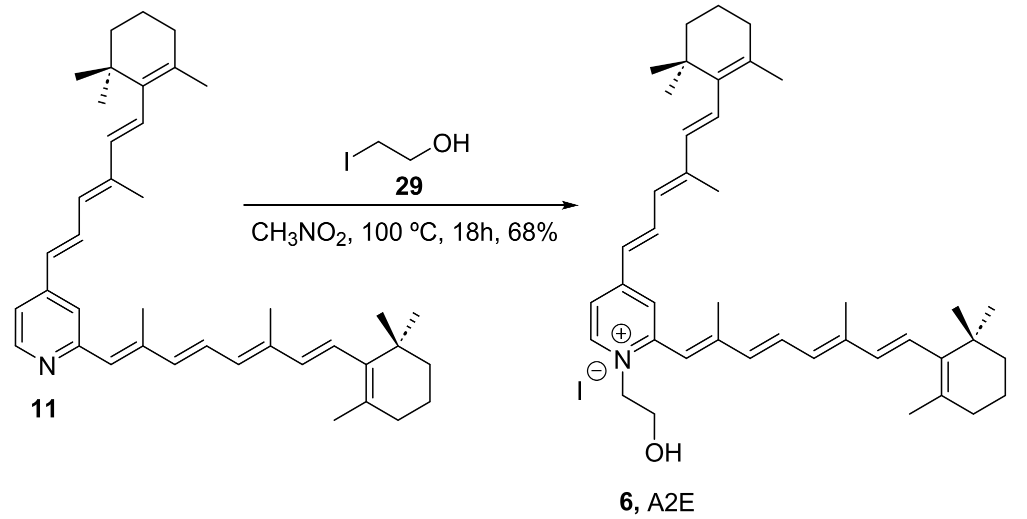

6, A2E

To a solution of **11** (33.0 mg, 0.06 mmol) in CH_3NO_2 (1 mL), 2-iodoethanol **29** (0.05 mL, 0.60 mmol) was added, and the resulting solution was heated at 100 °C for 18h. The solvent was evaporated under vacuum, the residue was triturated with *n*-hexane and Et_2O mixtures and the solvents were removed to afford 29.2 mg (68% yield) of a dark

red syrup, which was identified as A2E (**6**). The spectroscopic data of the obtained compound matched those for the same product previously reported in the literature.[1]

2-((1*E*,3*E*,5*E*,7*E*)-8-Iodo-3,7-dimethylocta-1,3,5,7-tetraen-1-yl)-1,3,3-trimethylcyclohex-1-ene (43).

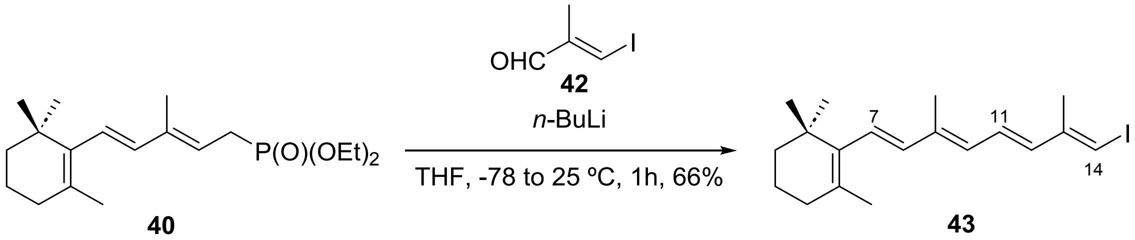

To a cooled (-78 °C) stirred solution of phosphonate **40** (0.46 g, 1.35 mmol) in THF (6 mL), DMPU (2.9 mL) and *n*-BuLi (0.71 mL, 1.90 M in hexanes, 1.35 mmol) were added. Subsequently, **42** [5] (0.23 g, 1.17 mmol) in THF (6 mL) was added and the mixture was allowed to warm up to room temperature for 1h. A saturated aqueous solution of NH_4Cl was added and the mixture was extracted with Et_2O (3x). The combined organic layers were washed with brine, dried over Na_2SO_4, filtered and the solvent was evaporated. The residue was purified by flash column chromatography (C18 silica gel, from 50:50 to 100:0 *v*/*v* CH_3CN/H_2O) to afford 0.29 g (66% yield) of yellow oil, which was identified as 2-((1*E*,3*E*,5*E*,7*E*)-8-iodo-3,7-dimethylocta-1,3,5,7-tetraen-1-yl)-1,3,3-trimethylcyclohex-1-ene **43**. **^{1}H-NMR** (400.16 MHz, C_6D_6): δ 6.57 (dd, *J* = 15.2, 11.2 Hz, 1H, H_{11}), 6.31 (d, *J* = 16.2 Hz, 1H, H_7), 6.26 (d, *J* = 16.2 Hz, 1H, H_8), 6.10 (s, 1H, H_{14}), 6.07 (d, *J* = 15.2 Hz, 1H, H_{12}), 6.03 (d, *J* = 11.2 Hz, 1H, H_{10}), 1.96 (t, *J* = 6.3 Hz, 2H, CH_2), 1.86 (d, *J* = 1.0 Hz, 3H, CH_3), 1.79 (d, *J* = 1.2 Hz, 3H, CH_3), 1.77 (d, *J* = 0.9 Hz, 3H, CH_3), 1.63 – 1.55 (m, 2H, CH_2), 1.52 – 1.45 (m, 2H, CH_2), 1.12 (s, 6H, 2xCH_3) ppm. **^{13}C{^{1}H}-NMR** (100.63 MHz, C_6D_6): δ 145.8, 138.4, 138.3, 137.4, 133.4, 130.5, 129.5, 127.5, 126.1, 84.0, 40.0, 34.6, 33.4, 29.2 (2x), 22.0, 20.1, 19.7, 12.9 ppm. **IR** (NaCl): υ 2956 (s, C-H), 2925 (s, C-H), 2861 (m, C-H), 1456 (w, C=C), 964 (s, C-H) cm^{-1}. **HRMS** (ESI^+): calcd. for $C_{19}H_{28}I$ ($[M+H]^+$), 383.1236; found, 383.1230.

Compound 44.

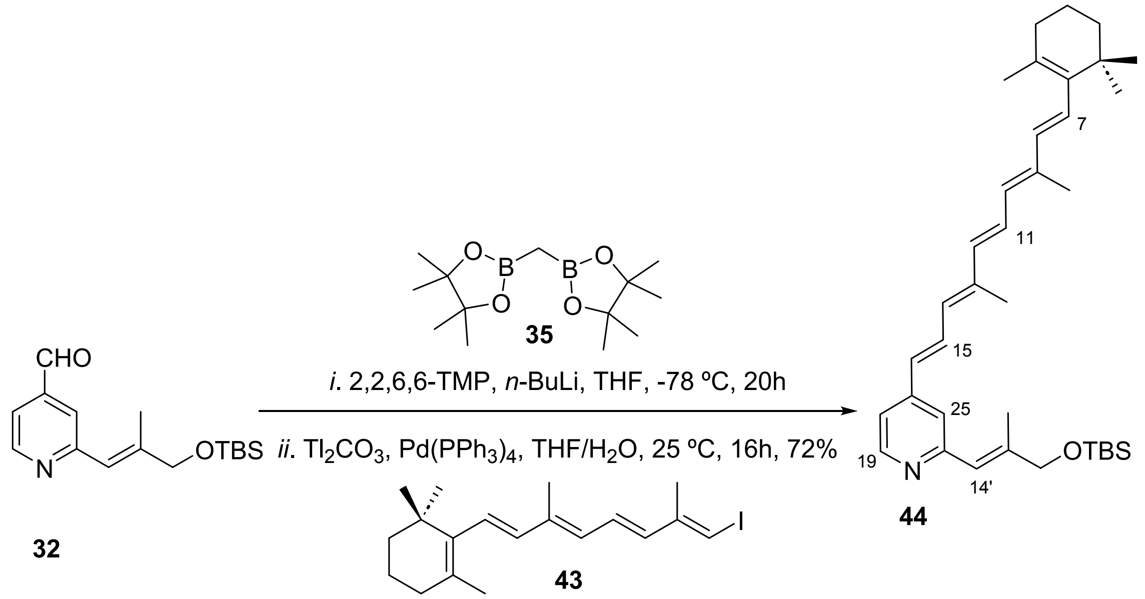

To a cooled (0 °C) solution of 2,2,6,6-tetramethylpiperidine (0.63 mL, 3.76 mmol) in THF (1 mL), *n*-BuLi (1.9 mL, 1.9 M in hexanes, 3.76 mmol) was added and the mixture was stirred for 30 min. Then, a solution of bis(pinacolatoboryl)methane **35** (1.0 g, 3.76 mmol) in THF (3 mL) was added and the reaction mixture was stirred for 10 min. The mixture was cooled down to -78 °C, a solution of **32** [4] (0.27 g, 0.94 mmol) in THF (3 mL) was added dropwise, and the reaction mixture was stirred at -78 °C for 20h. A saturated aqueous solution of NH_4Cl was added and the mixture was extracted with Et_2O (3x). The combined organic layers were washed with brine, dried over anhydrous Na_2SO_4, filtered and the solvent was evaporated.

To a cooled (0 °C) solution of 2-((1*E*,3*E*,5*E*,7*E*)-8-iodo-3,7-dimethylocta-1,3,5,7-tetraen-1-yl)-1,3,3-trimethylcyclohex-1-ene **43** (0.26 g, 0.68 mmol) and the crude obtained above (0.19 g, 0.57 mmol) in a THF-water mixture (19 mL, 4:1 *v*/*v*), Tl_2CO_3 (0.67 g, 1.42 mmol) and $Pd(PPh_3)_4$ (32.9 mg, 0.03 mmol) were added, and the reaction mixture was stirred at 25 °C for 16h. The mixture was filtered through Celite® and Na_2SO_4 washing with Et_2O, and the solvent was evaporated. The residue was purified by flash column chromatography (silica gel, 98:2 *v*/*v* *n*-hexane/Et_3N; then, from 100:0 to 90:10 *v*/*v* *n*-hexane/EtOAc) to afford 0.22 g (72% yield) of a dark yellow oil, which was identified as **44**. **^{1}H-NMR** (400.16 MHz, C_6D_6): δ 8.57 (d, *J* = 5.1 Hz, 1H, H_{19}), 7.19 (dd, *J* = 15.1, 11.4 Hz, 1H, H_{15}), 7.11 (s, 1H, H_{25}), 6.85 (d, *J* = 1.6 Hz, 1H, $H_{14'}$), 6.82 – 6.73 (m, 2H, $H_{11} + H_{18}$), 6.40 (d, *J* = 15.1 Hz, 1H, H_{12}), 6.34 (m, 2H, $H_7 + H_8$), 6.29 (d, *J* = 11.4 Hz, 1H, H_{10}), 6.24 (d,

J = 15.4 Hz, 1H, H_{16}), 6.20 (d, J = 11.7 Hz, 1H, H_{14}), 4.14 (s, 2H, 2x$CH_{12'}$), 2.31 (s, 3H, CH_3), 1.98 (t, J = 6.3 Hz, 2H, CH_2), 1.92 (d, J = 1.1 Hz, 3H, CH_3), 1.83 (s, 3H, CH_3), 1.80 (s, 3H, CH_3), 1.64 – 1.56 (m, 2H, CH_2), 1.53 – 1.47 (m, 2H, CH_2), 1.14 (s, 6H, 2xCH_3), 1.02 (s, 9H, $SiC(\underline{CH_3})_3$), 0.11 (s, 6H, 2xSi-CH_3) ppm. **$^{13}C\{^1H\}$-NMR** (100.63 MHz, C_6D_6): δ 158.4, 149.9, 145.0, 142.9, 139.1, 138.5, 138.3, 137.5, 136.9, 131.6, 131.5, 130.7, 129.6, 129.1, 127.5, 126.7, 123.3, 122.0, 117.5, 68.8, 40.0, 34.6, 33.4, 29.3 (2x), 26.2 (3x), 22.1, 19.8, 18.7, 15.9, 13.0, 12.9, -5.1 (2x) ppm. **IR** (NaCl): υ 2954 (s, C-H), 2929 (s, C-H), 2856 (m, C-H), 1595 (m, C=C), 1253 (m, C-O), 1113 (m, Si-O-C), 837 (s, C-H) cm^{-1}. **HRMS** (ESI^+): calcd. for $C_{36}H_{54}NOSi$ ($[M+H]^+$), 544.3975; found, 544.3969.

Compound 45.

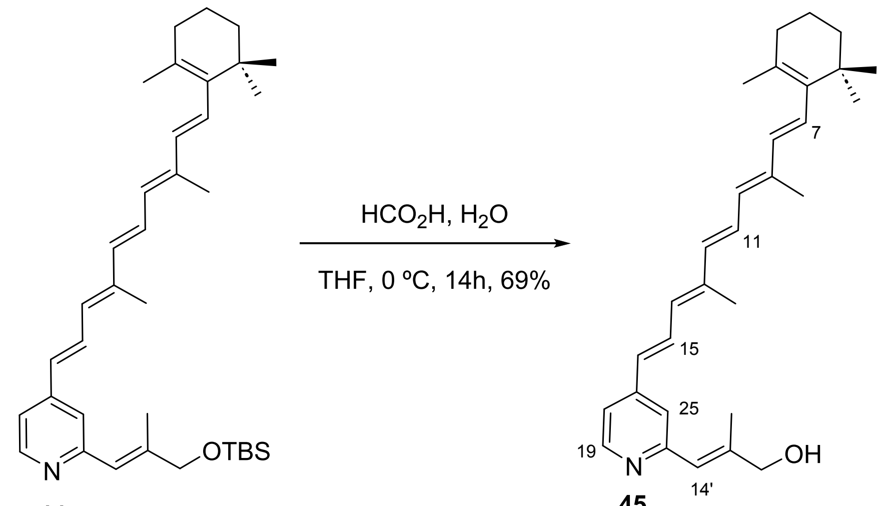

44 **45**

A cooled (0 °C) mixture of HCO_2H (2.5 mL, 66.56 mmol) and H_2O (0.8 mL, 85.60 mmol) was added to a cooled (0 °C) solution of **44** (100 mg, 0.19 mmol) in THF (5 mL). The mixture was stirred at 0 °C for 14h. A saturated aqueous solution of $NaHCO_3$ was then added and the mixture was extracted with EtOAc (3x). The combined organic layers were dried over Na_2SO_4, filtered and the solvent was evaporated. The residue was purified by flash column chromatography (silica gel, 98:2 *v/v* *n*-hexane/Et_3N; then, from 70:30 to 50:50 *v/v* *n*-hexane/EtOAc) to afford 57 mg (69% yield) of a dark red oil, which was identified as **45**. **^1H-NMR** (400.16 MHz, C_6D_6): δ 8.54 (br s, 1H, H_{19}), 7.21 (dd, J = 15.3, 11.5 Hz, 1H, H_{15}), 7.09 (s, 1H, H_{25}), 6.89 (s, 1H, $H_{14'}$), 6.81 (dd, J = 15.0, 11.4 Hz, 1H, H_{11}), 6.75 (d, J = 5.1 Hz, 1H, H_{18}), 6.41 (d, J = 15.0 Hz, 1H, H_{12}), 6.38 (d, J = 16.0 Hz, 1H, H_7), 6.34 (d, J = 16.0 Hz, 1H, H_8), 6.31 (d, J = 11.4 Hz, 1H, H_{10}), 6.26 (d, J = 15.3 Hz, 1H, H_{16}),

6.20 (d, J = 11.5 Hz, 1H, H_{14}), 4.12 (s, 2H, <u>CH</u>$_2$OH), 2.19 (s, 3H, CH_3), 1.98 (t, J = 6.3 Hz, 2H, CH_2), 1.94 (d, J = 1.0 Hz, 3H, CH_3), 1.85 (s, 3H, CH_3), 1.81 (s, 3H, CH_3), 1.64 – 1.56 (m, 2H, CH_2), 1.54 – 1.46 (m, 2H, CH_2), 1.15 (s, 6H, 2xCH_3) ppm. **^{13}C{^{1}H}-NMR** (100.63 MHz, C_6D_6): δ 158.4, 149.7, 145.3, 144.2, 139.3, 138.5, 138.3, 137.4, 137.0, 131.6, 131.5, 130.5, 129.7, 129.4, 127.5, 126.8, 123.1, 121.9, 117.8, 68.1, 40.0, 34.6, 33.4, 29.2 (2x), 22.0, 19.7, 15.9, 13.0, 12.9 ppm. **IR** (NaCl): υ 3500 – 3200 (br, O-H), 2955 (m, C-H), 2926 (m, C-H), 2855 (m, C-H), 1595 (m, C=C), 1253 (m, C-O), 1113 (m, Si-O-C), 837 (s, C-H) cm^{-1}. **HRMS** (ESI$^+$): calcd. for $C_{30}H_{40}NO$ ([M+H]$^+$), 430.3110; found, 430.3104.

Compound 41.

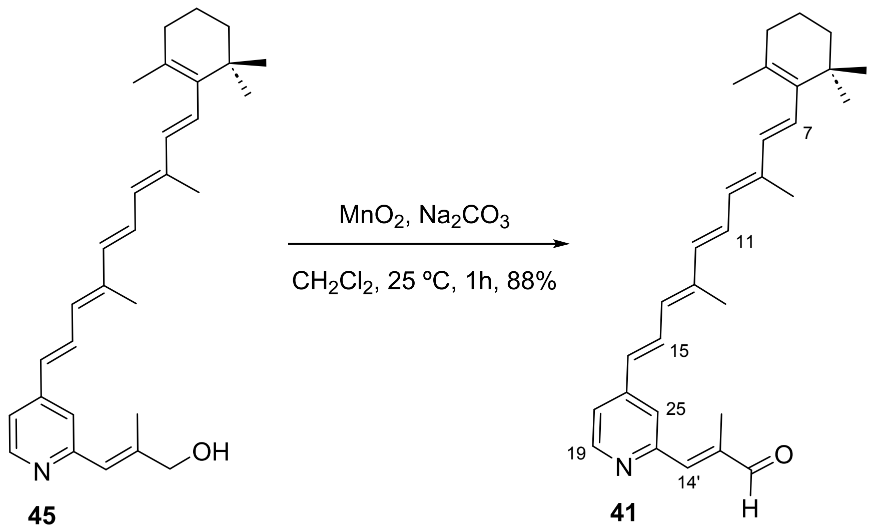

To a stirred solution of **45** (55 mg, 0.13 mmol) in CH_2Cl_2 (3 mL) at room temperature, Na_2CO_3 (244 mg, 2.30 mmol) and MnO_2 (200 mg, 2.30 mmol) were added. The resulting reaction mixture was stirred at room temperature for 1h. The mixture was filtered through Celite® washing with CH_2Cl_2 to afford 48 mg (88% yield) of a dark red oil, which was identified as **41**. **^{1}H-NMR** (400.16 MHz, C_6D_6): δ 9.50 (s, 1H, CHO), 8.48 (d, J = 5.1 Hz, 1H, H_{19}), 7.21 (dd, J = 15.4, 11.5 Hz, 1H, H_{15}), 6.97 (s, 1H, H_{25}), 6.85 (dd, J = 15.1, 11.4 Hz, 1H, H_{11}), 6.77 (d, J = 1.5 Hz, 1H, $H_{14'}$), 6.71 (dd, J = 5.2, 1.7 Hz, 1H, H_{18}), 6.43 (d, J = 15.1 Hz, 1H, H_{12}), 6.38 (app. s, 2H, H_7 + H_8), 6.32 (d, J = 11.4 Hz, 1H, H_{10}), 6.24 (d, J = 11.5 Hz, 1H, H_{14}), 6.21 (d, J = 15.4 Hz, 1H, H_{16}), 2.50 (d, J = 1.4 Hz, 3H, CH_3), 1.99 (t, J = 6.3 Hz, 2H, CH_2), 1.95 (s, 3H, CH_3), 1.89 (s, 3H, CH_3), 1.81 (s, 3H, CH_3), 1.67 – 1.58 (m, 2H, CH_2), 1.53 – 1.46 (m, 2H, CH_2), 1.15 (s, 6H, 2xCH_3) ppm. **^{13}C{^{1}H}-NMR** (100.63 MHz, C_6D_6): δ 194.9,

155.7, 150.5, 146.6, 145.4, 142.2, 140.0, 138.4, 138.3, 137.5, 137.1, 131.3, 131.1, 130.0, 129.8, 129.5, 127.9, 127.3, 123.7, 119.7, 40.0, 34.6, 33.4, 29.2 (2x), 22.0, 19.7, 13.1, 12.9, 11.5 ppm. **IR** (NaCl): υ 2924 (s, C-H), 2860 (m, C-H), 1685 (s, C=O), 1591 (m, C=C) cm^{-1}. **HRMS** (ESI^+): calcd. for $C_{30}H_{38}NO$ ($[M+H]^+$), 428.2953; found, 428.2948.

Compound 16.

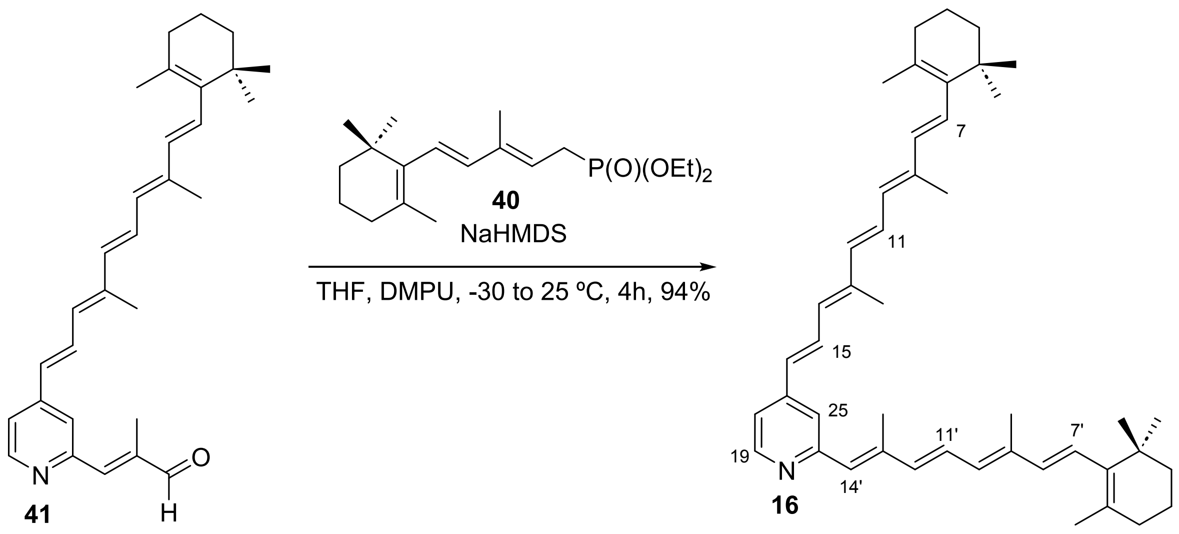

To a cooled (-30 °C) stirred solution of phosphonate **40** (63.6 mg, 0.19 mmol) in THF (0.5 mL), DMPU (0.3 mL) and NaHMDS (0.18 mL, 1M in THF, 0.18 mmol) were added. Subsequently, **41** (47.0 mg, 0.11 mmol) in THF (0.5 mL) was added and the mixture was allowed to warm up to room temperature for 4h. A saturated aqueous solution of NH_4Cl was added and the mixture was extracted with Et_2O (3x). The combined organic layers were washed with brine, dried over Na_2SO_4, filtered and the solvent was evaporated. The residue was purified by flash column chromatography (silica gel, 98:2 *v*/*v* *n*-hexane/Et_3N; then, from 100:0 to 90:10 *v*/*v* *n*-hexane/EtOAc) to afford 63.5 mg (94% yield) of a dark red foam, which was identified as **16**. **^{1}H-NMR** (400.16 MHz, C_6D_6): δ 8.59 (d, J = 5.1 Hz, 1H, H_{19}), 7.23 (dd, J = 15.3, 11.5 Hz, 1H, H_{15}), 7.04 (s, 1H, H_{25}), 6.98 (dd, J = 15.1, 11.3 Hz, 1H, $H_{11'}$), 6.83 (dd, J = 15.0, 11.4 Hz, 1H, H_{11}), 6.73 (dd, J = 5.2, 1.6 Hz, 1H, H_{18}), 6.60 (s, 1H, $H_{14'}$), 6.59 (d, J = 15.1 Hz, 1H, $H_{12'}$), 6.44 (d, J = 15.0 Hz, 1H, H_{12}), 6.39 (d, J = 16.0 Hz, 1H, $H_{7'}$), 6.37 – 6.34 (m, 3H, $H_{8'}$ + $H_{10'}$ + H_7), 6.34 – 6.31 (m, 2H, H_{10} + H_8), 6.27 (d, J = 15.3 Hz, 1H, H_{16}), 6.22 (d, J = 11.5 Hz, 1H, H_{14}), 2.73 (d, J = 1.1 Hz, 3H, CH_3), 1.99 (t, J = 6.3 Hz, 4H, 2xCH_2), 1.95 (d, J = 1.1 Hz, 3H, CH_3), 1.91 (d, J = 1.1 Hz, 3H, CH_3), 1.87

(d, J = 1.1 Hz, 3H, CH_3), 1.82 (s, 6H, 2xCH_3), 1.64 – 1.58 (m, 4H, 2xCH_2), 1.54 – 1.48 (m, 4H, 2xCH_2), 1.16 (s, 6H, 2xCH_3), 1.15 (s, 6H, 2xCH_3) ppm. **^{13}C{^{1}H}-NMR** (100.63 MHz, C_6D_6): δ 158.4, 150.0, 145.0, 141.5, 139.2, 139.1, 138.7, 138.5, 138.4, 138.3, 137.4, 137.0, 136.7, 131.6, 131.5, 130.9, 130.6, 129.7, 129.4, 129.2, 127.9, 127.6, 127.2, 127.1, 126.8, 122.9, 117.6, 40.0 (2x), 34.6 (2x), 33.4, 33.3, 29.2 (4x), 22.0 (2x), 19.8, 19.7, 14.5, 13.0, 12.9 (2x) ppm. **IR** (NaCl): υ 2953 (m, C-H), 2924 (s, C-H), 2861 (m, C-H), 1455 (m, C=C), 965 (s, C-H) cm^{-1}. **HRMS** (ESI$^+$): calcd. for $C_{45}H_{60}N$ ([M+H]$^+$), 614.4726; found, 614.4720. **UV** (CH_3OH): λ_{max} 411 nm (ε = 30,000).

pd-A2E (9).

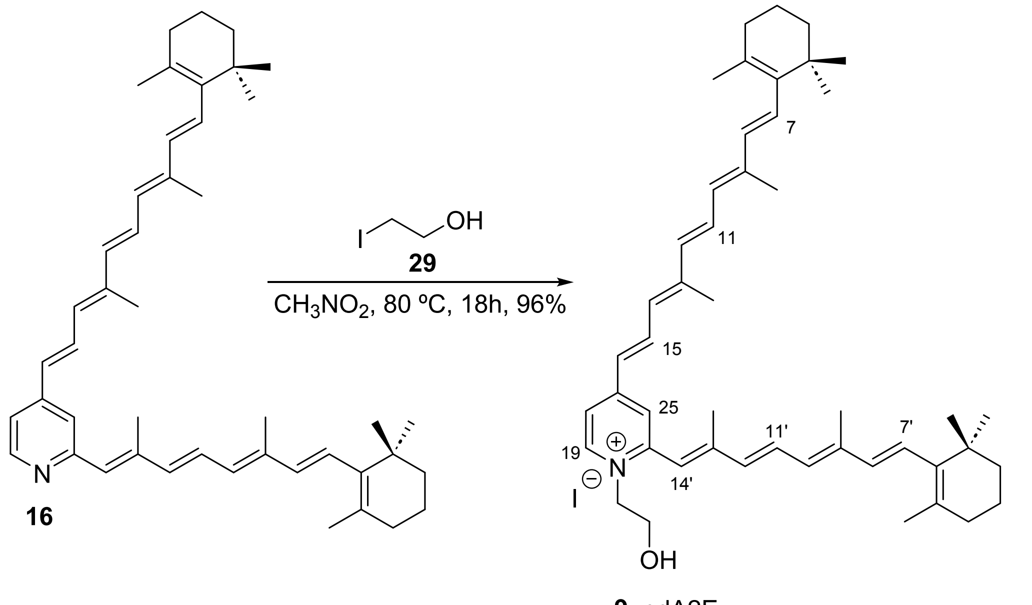

9, pdA2E

To a stirred solution of **16** (10.0 mg, 0.02 mmol) in CH_3NO_2 (0.3 mL), 2-iodoethanol **29** (0.01 mL, 0.16 mmol) was added, and the resulting solution was heated at 80 °C for 18h. The solvent was evaporated under vacuum, the residue was triturated with *n*-hexane and Et_2O mixtures and the solvents were removed to afford 10.3 mg (96% yield) of a dark red syrup, which was identified as pd-A2E (**9**). **^{1}H-NMR** (400.16 MHz, C_6D_6): δ 8.53 (d, J = 6.8 Hz, 1H, H_{19}), 7.99 (dd, J = 15.1, 11.7 Hz, 1H, H_{15}), 7.93 (d, J = 6.8 Hz, 1H, H_{18}), 7.85 (d, J = 2.1 Hz, 1H, H_{25}), 7.12 (dd, J = 15.0, 11.3 Hz, 1H, $H_{11'}$), 7.01 (dd, J = 15.0, 11.5 Hz, 1H, H_{11}), 6.77 (d, J = 15.1 Hz, 1H, H_{16}), 6.70 (s, 1H, $H_{14'}$), 6.63 (d, J = 15.0 Hz, 1H, $H_{12'}$), 6.52 (d, d, J = 15.0 Hz, 1H, H_{12}), 6.47 (d, J = 11.7 Hz, 1H, H_{14}), 6.36 (d, J = 16.0 Hz, 1H, $H_{7'}$), 6.31 (d, J = 11.3 Hz, 1H, $H_{10'}$), 6.25 (d, J = 16.0 Hz, 1H, H_7), 6.22 (d, J = 11.5 Hz, 1H, H_{10}), 6.21 – 6.14 (m, 2H, H_8 + $H_{8'}$), 4.54 (t, J = 5.0 Hz, 2H, CH_2), 3.92 (t, J = 5.0 Hz, 2H, CH_2), 2.20

(s, 3H, CH_3), 2.16 (s, 3H, CH_3), 2.11 – 2.00 (m, 10H, $2xCH_2 + 2xCH_3$), 1.73 (s, 6H, $2xCH_3$), 1.69 – 1.59 (m, 4H, $2xCH_2$), 1.54 – 1.48 (m, 4H, $2xCH_2$), 1.05 (s, 6H, $2xCH_3$), 1.04 (s, 6H, $2xCH_3$) ppm. **$^{13}C\{^1H\}$-NMR** (100.63 MHz, C_6D_6): δ 154.5, 153.3, 148.9, 147.4, 146.0, 140.9, 140.0, 139.2, 139.1, 139.1, 139.0, 138.9, 137.5, 135.6, 132.1, 131.8, 131.7, 130.9, 130.8, 130.8, 130.7, 129.8, 129.4, 127.2, 127.1, 121.1, 120.5, 61.1, 60.0, 40.8, 40.7, 35.3 (2x), 34.0, 33.9, 29.4 (4x), 22.0, 21.9, 20.3 (2x), 15.0, 13.4, 13.0, 12.9 ppm. **HRMS** (ESI^+): calcd. for $C_{47}H_{64}NO$ ($[M]^+$), 658.4982; found, 658.4982. **UV** (CH_3OH): λ_{max} 338 nm (ε = 35,700), 490 nm (ε = 43,800). The spectroscopic data of the obtained compound matched those for the same product previously reported in the literature.[6]

3. **Comparison of ^{1}H-NMR spectra of pdA2E (9) (natural) and pdA2E (9) (synthetic).**

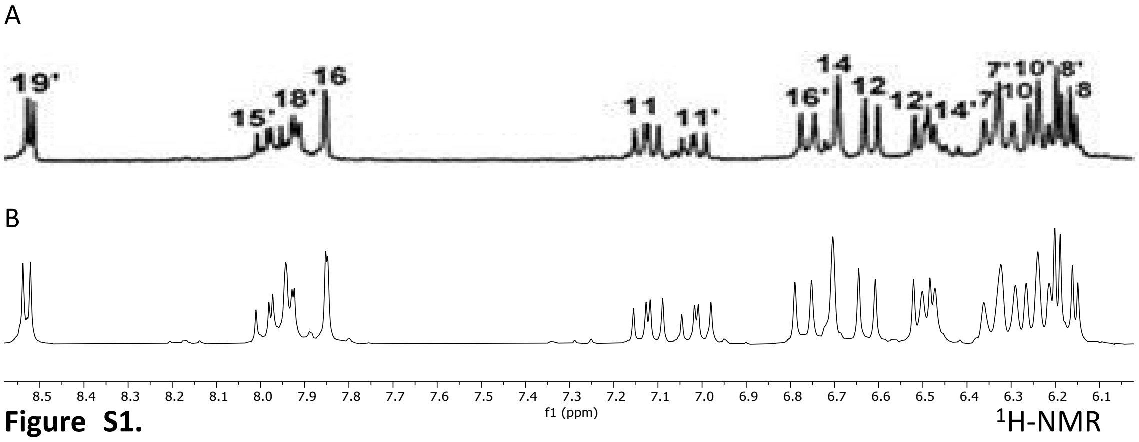

Figure S1. ^{1}H-NMR spectral regions of (**A**) natural pdA2E (**9**) (500 MHz, CD_3OD),[6] and (**B**) synthetic pdA2E (**9**) (400 MHz, CD_3OD) corresponding to the heterocyclic and olefin protons.

4. References

(1) Ren, R. X. F.; Sakai, N.; Nakanishi, K. *J. Am. Chem. Soc.* **1997**, *119*, 3619–3620.

(2) Fontán, N.; Domínguez, M.; Álvarez, R.; de Lera, A. R. *Eur. J. Org. Chem.* **2011**, No. 33, 6704–6712.

(3) Domínguez, M.; Pequerul, R.; Alvarez, R.; Giménez-Dejoz, J.; Birta, E.; Porté, S.; Rühl, R.; Parés, X.; Farrés, J.; de Lera, A. R. *Tetrahedron* **2018**, *74*, 2567–2574.

(4) Vidal, B.; Rodríguez, R.; Peña-Gallego, Á.; Álvarez, R.; Martínez, C.; de Lera, A. R. *J. Org. Chem.* **2026**, *91*, 2141–2151.

(5) White, J. D.; Blakemore, P. R.; Green, N. J.; Hauser, E. B.; Holoboski, M. A.; Keown, L. E.; Nylund Kolz, C. S.; Phillips, B. W. *J. Org. Chem.* **2002**, *67*, 7750–7760.

(6) Wu, Y.; Jin, Q.; Yao, K.; Zhao, J.; Chen, J.; Wu, X.; Gan, L.; Li, J.; Song, X.; Liu, X.; Cai, X. *Biochem. J.* **2014**, *460*, 343–352.

5. Copies of NMR spectra

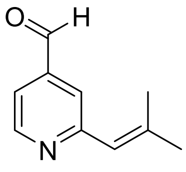

22

^{1}H-NMR (400.16 MHz, $CDCl_3$)

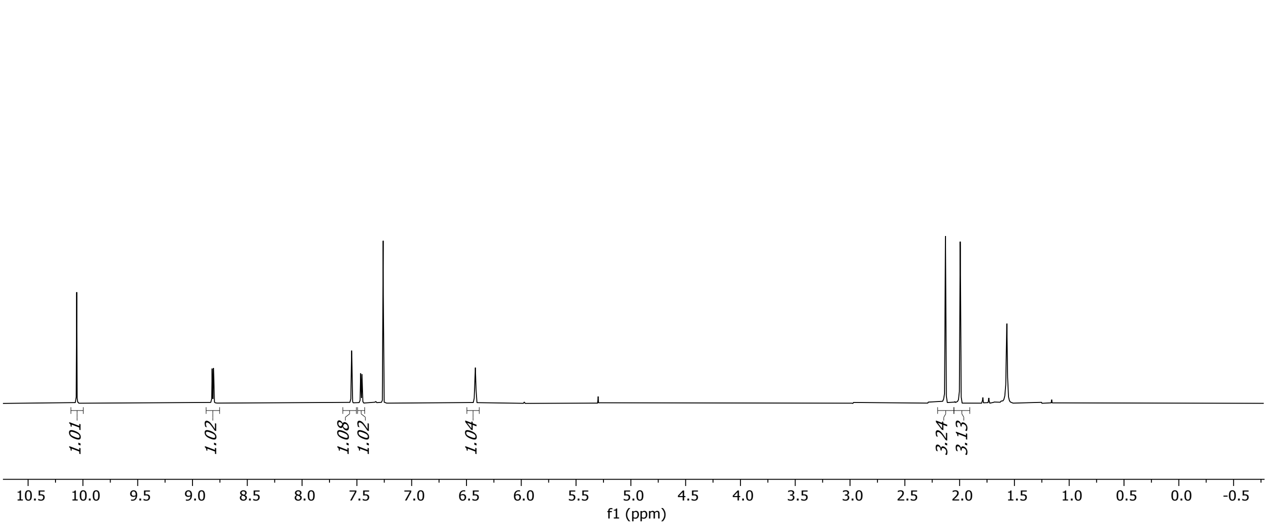

^{13}C{^{1}H}-NMR (100.63 MHz, $CDCl_3$)

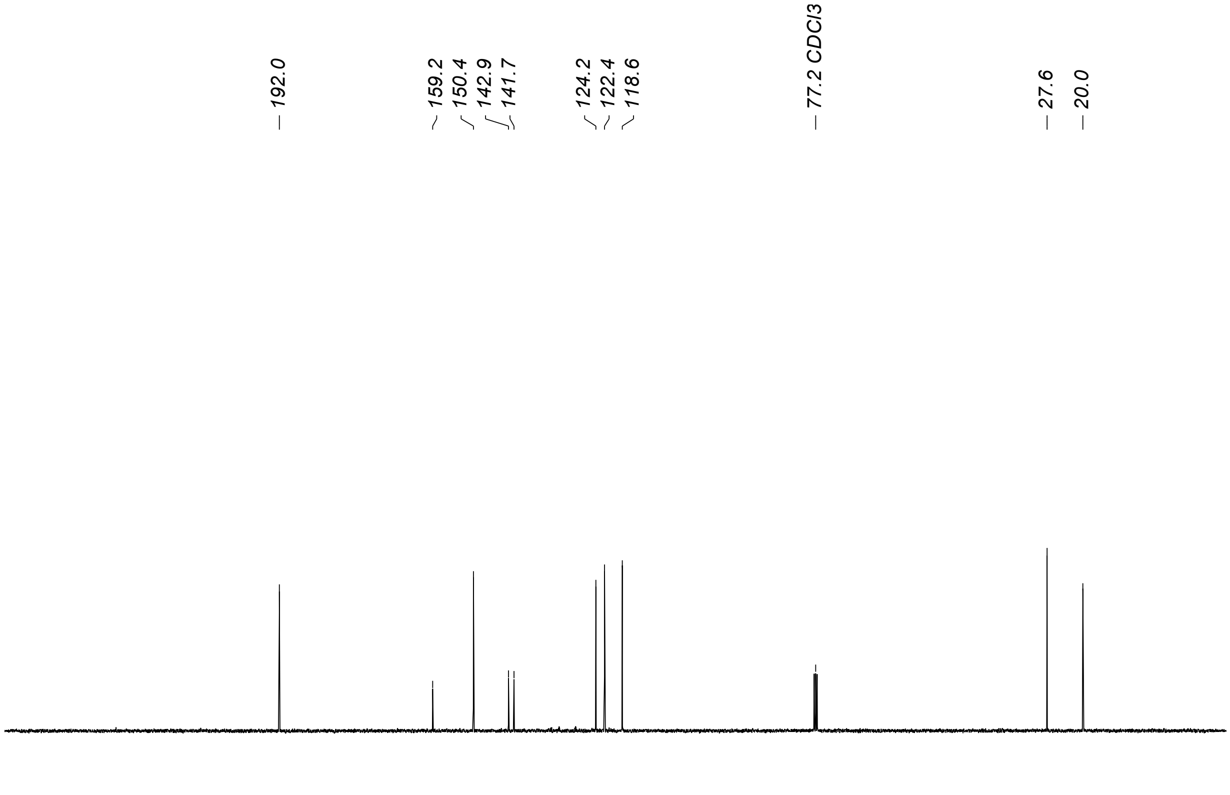

^{1}H-NMR (400.16 MHz, $CDCl_3$)

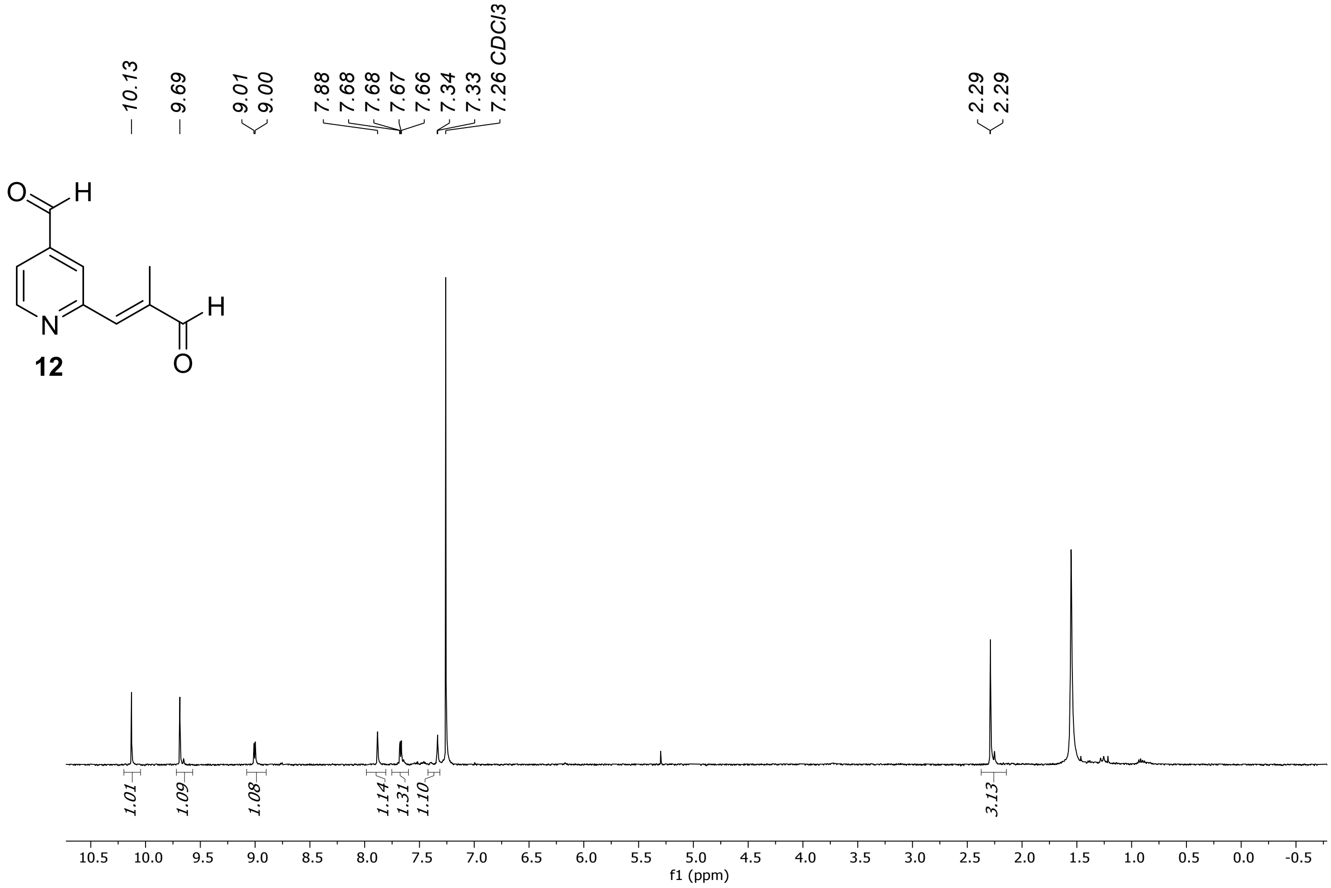

^{13}C{^{1}H}-NMR (100.63 MHz, $CDCl_3$)

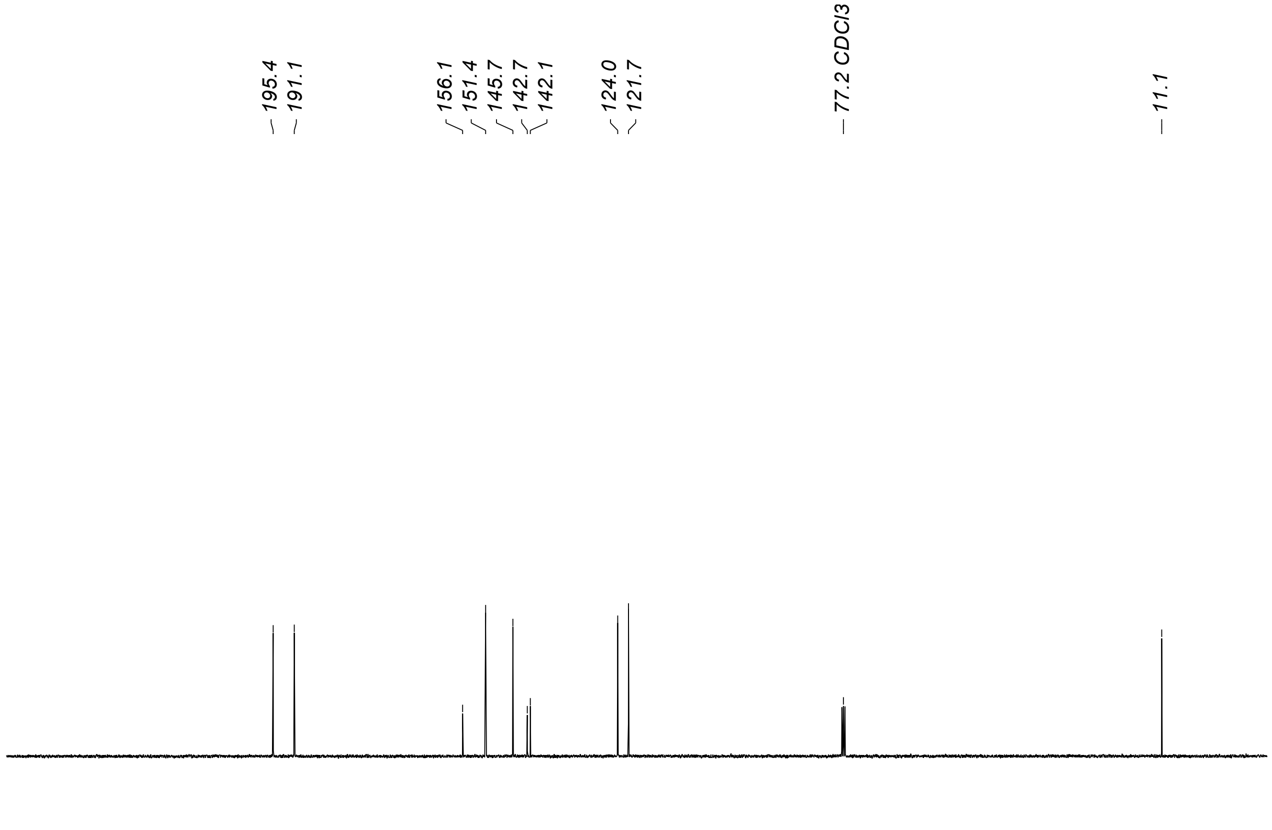

^{1}H-NMR (400.16 MHz, $CDCl_3$)

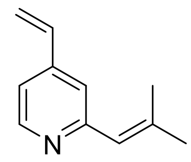

23

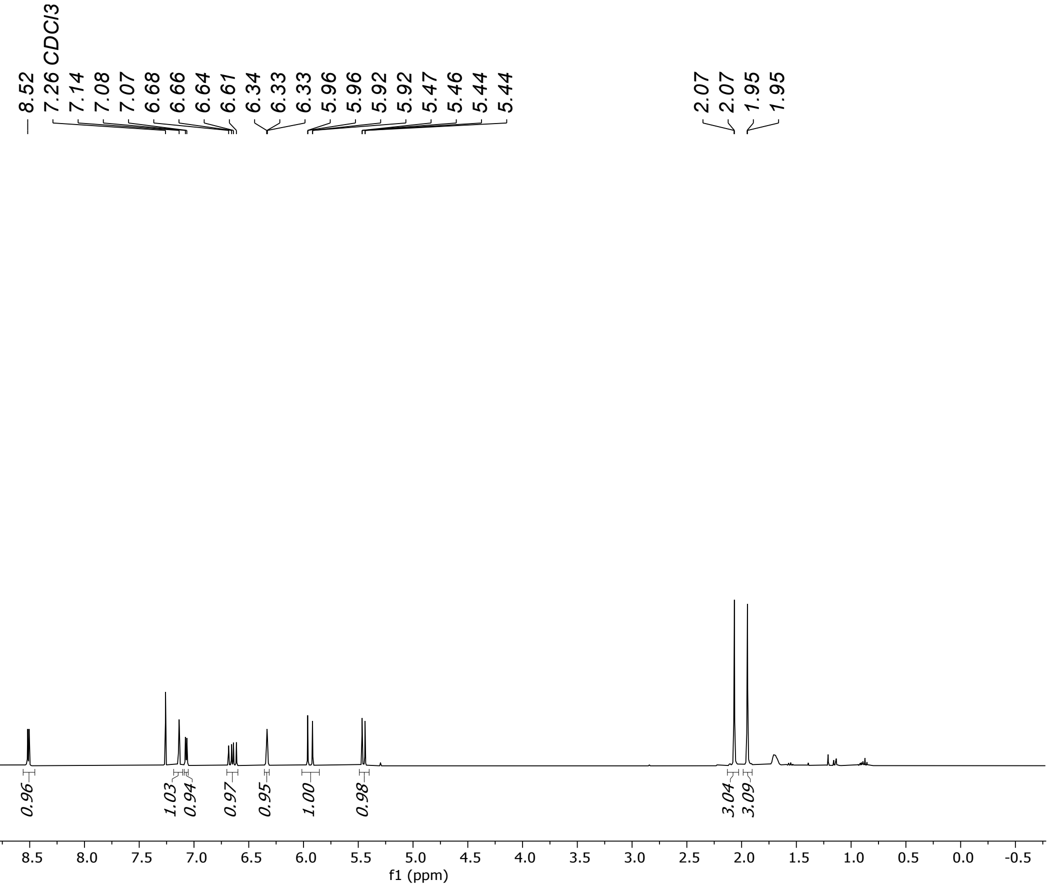

$^{13}C\{^{1}H\}$-NMR (100.63 MHz, $CDCl_3$)

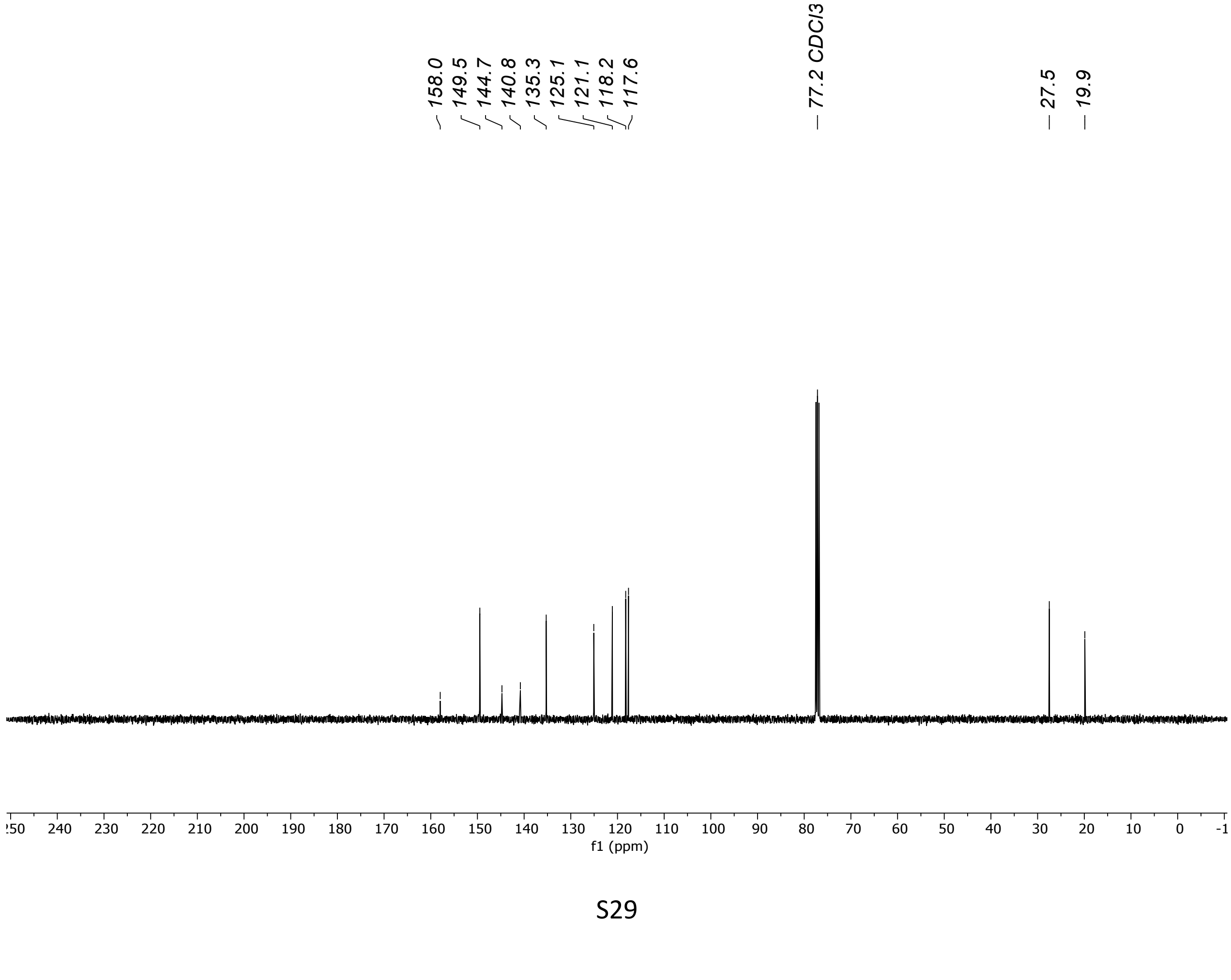

^{1}H-NMR (400.16 MHz, $CDCl_3$)

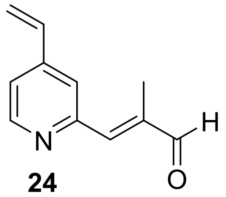

24

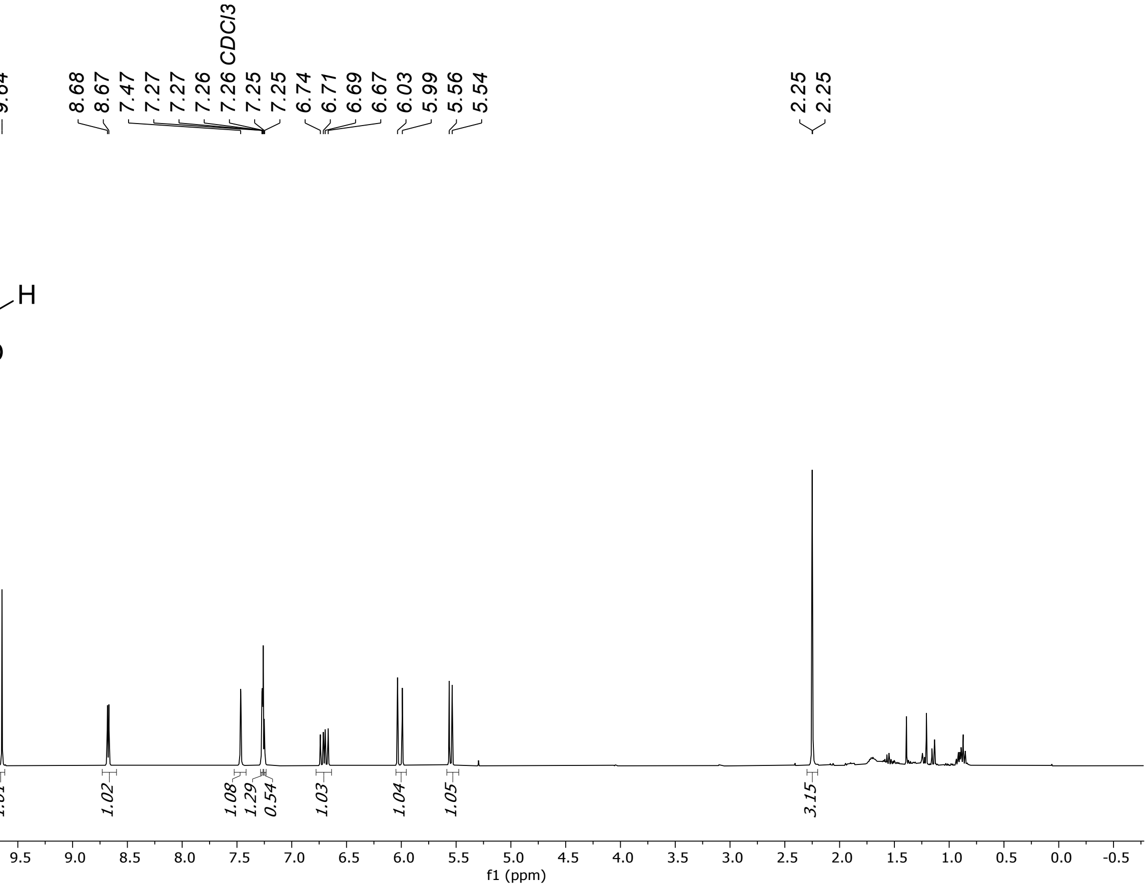

^{13}C{^{1}H}-NMR (100.63 MHz, $CDCl_3$)

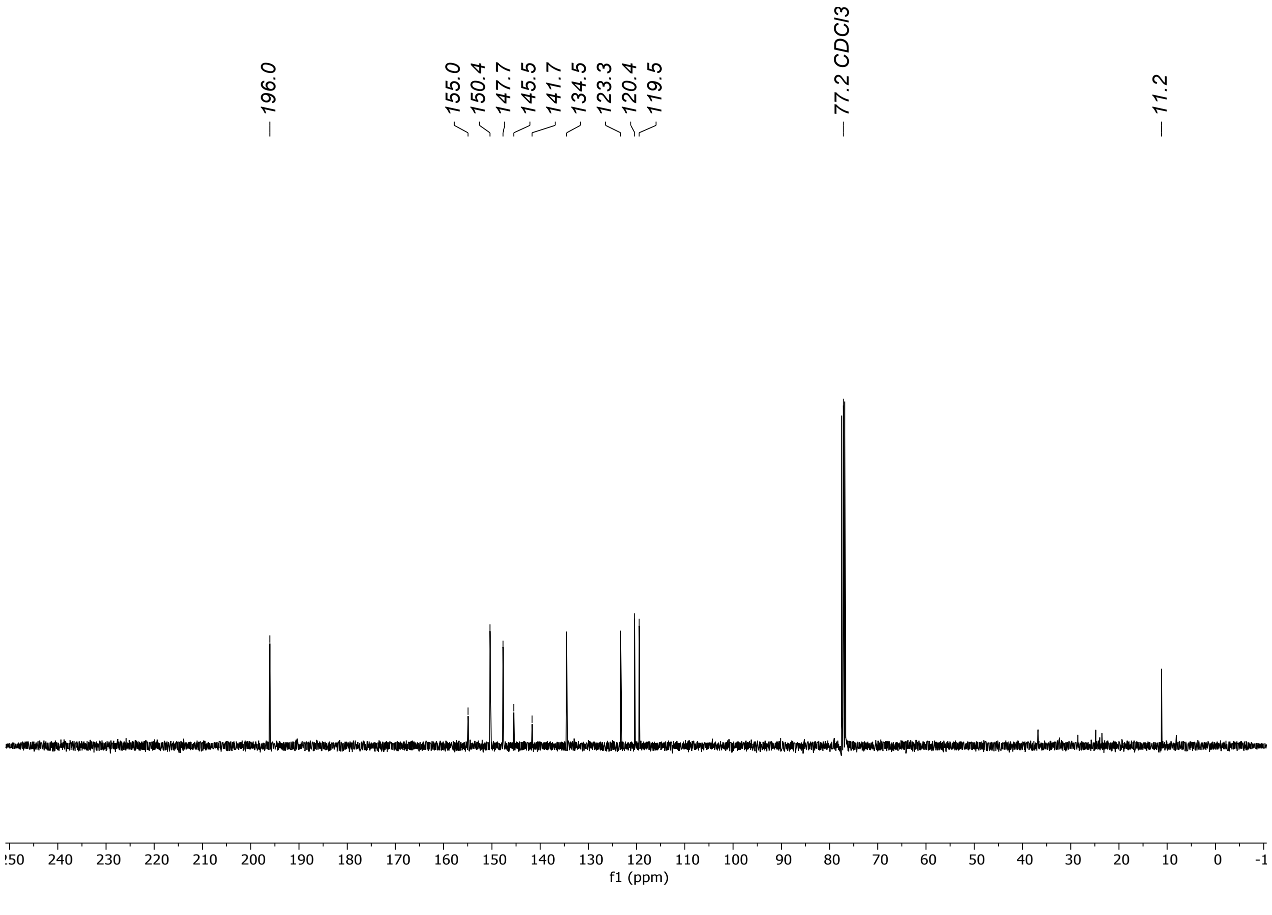

¹H-NMR (400.16 MHz, C_6D_6)

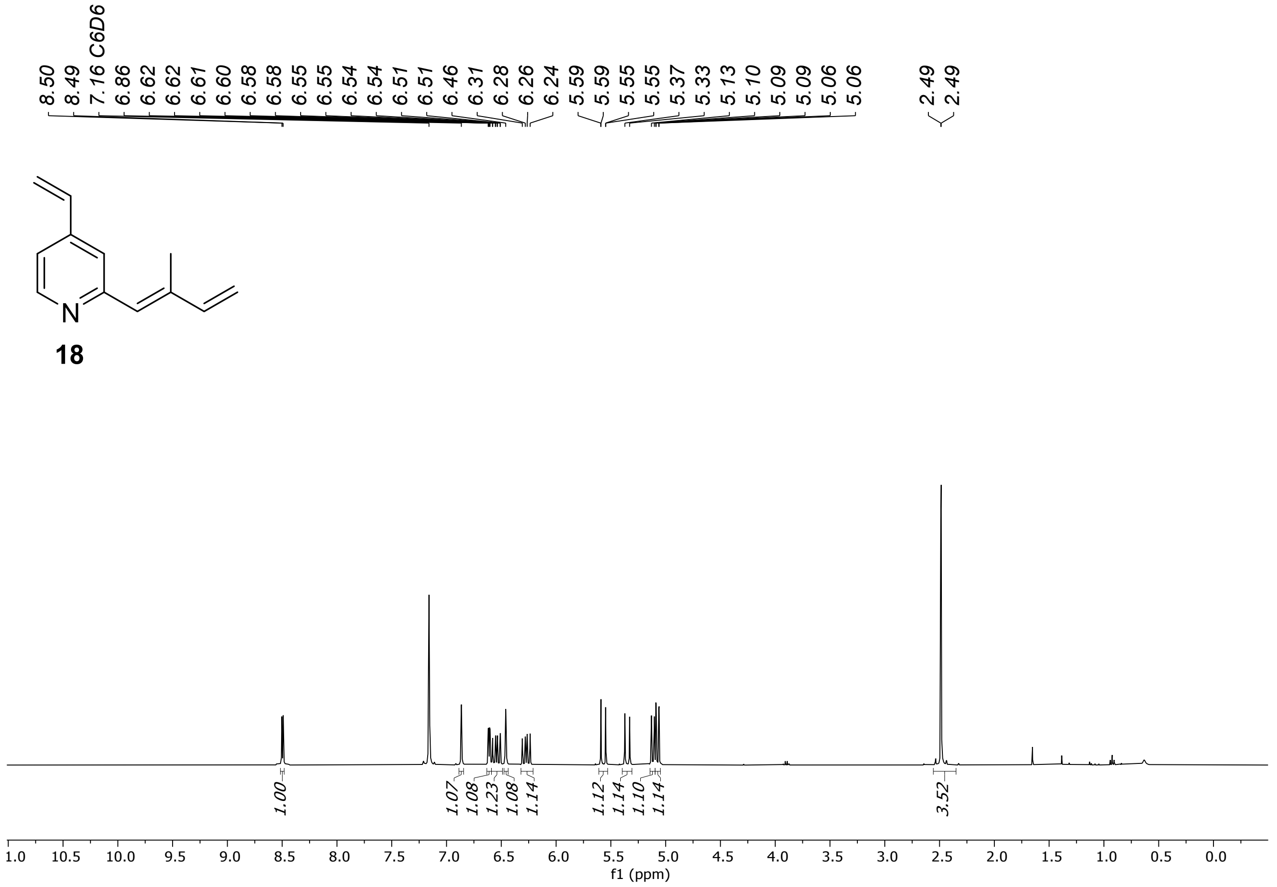

18

$^{13}C\{^1H\}$-NMR (100.63 MHz, C_6D_6)

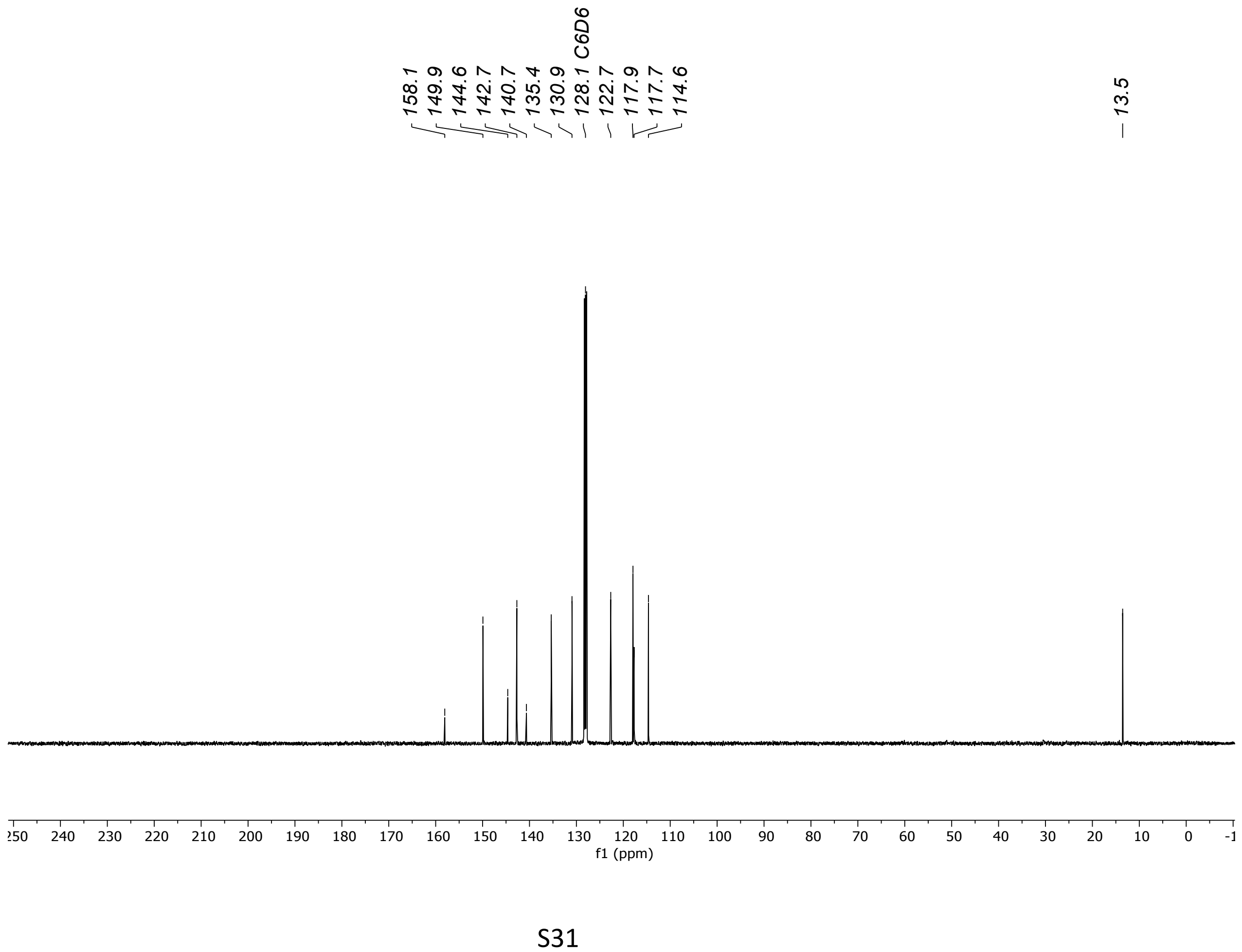

^{1}H-NMR (400.16 MHz, CD_2Cl_2)

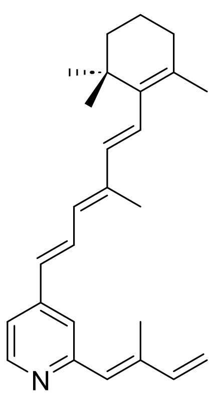

28

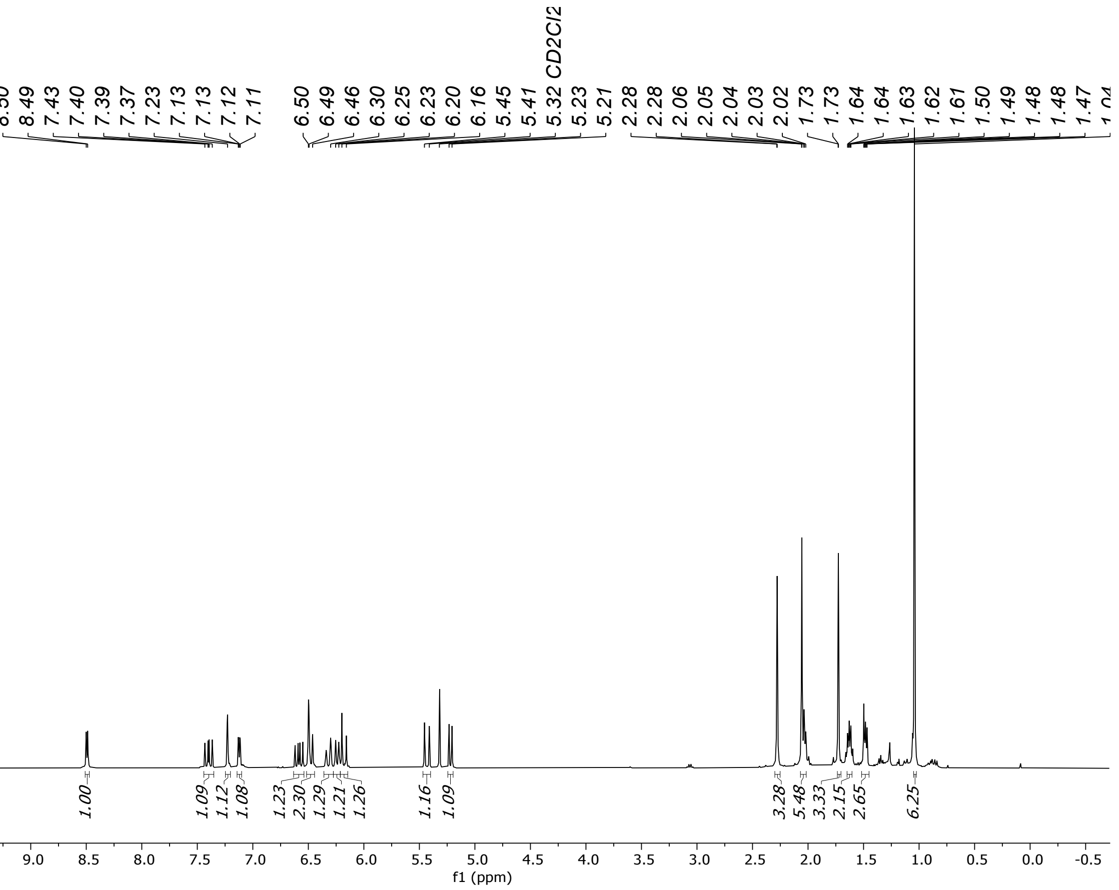

^{13}C{^{1}H}-NMR (100.63 MHz, CD_2Cl_2)

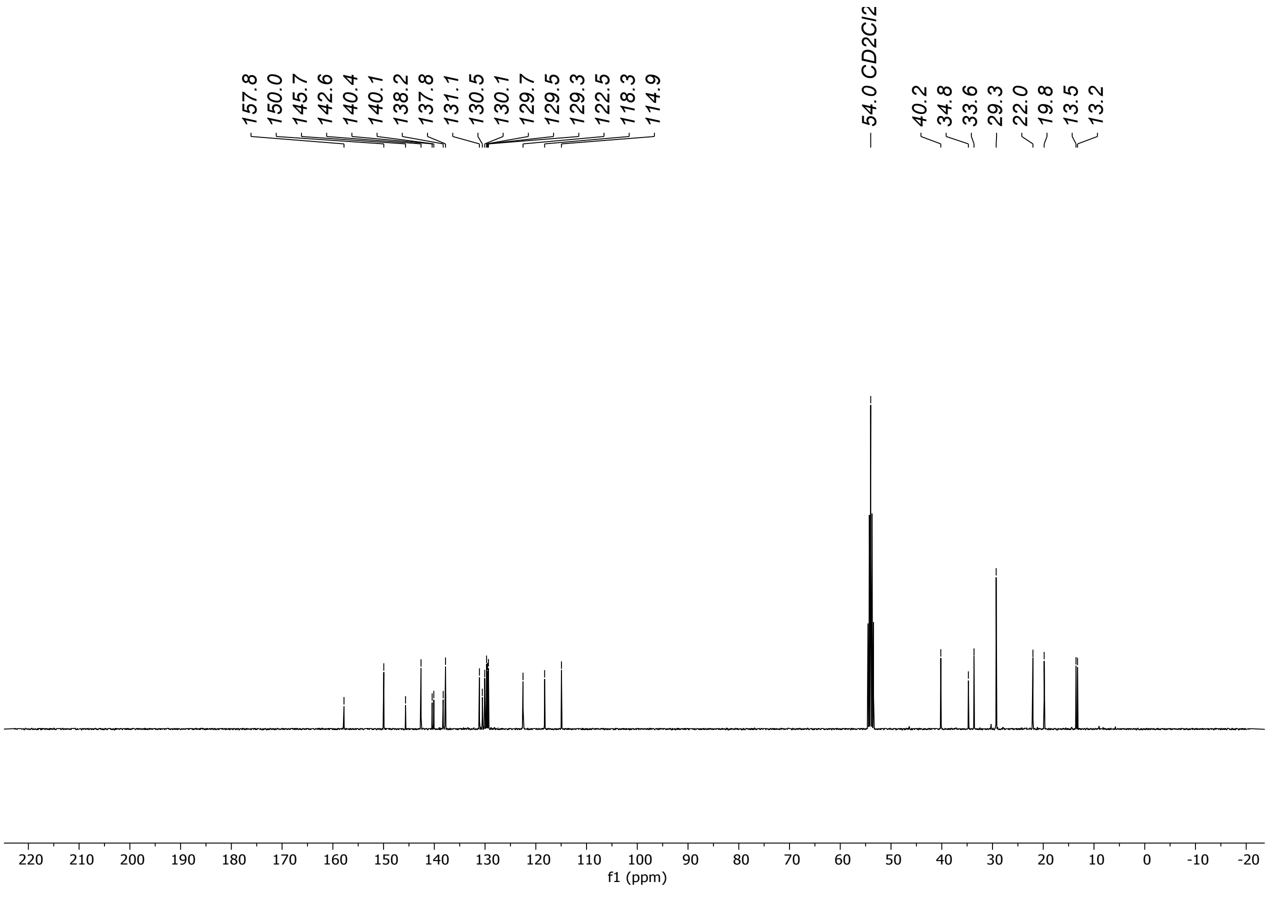

COSY (CD_2Cl_2)

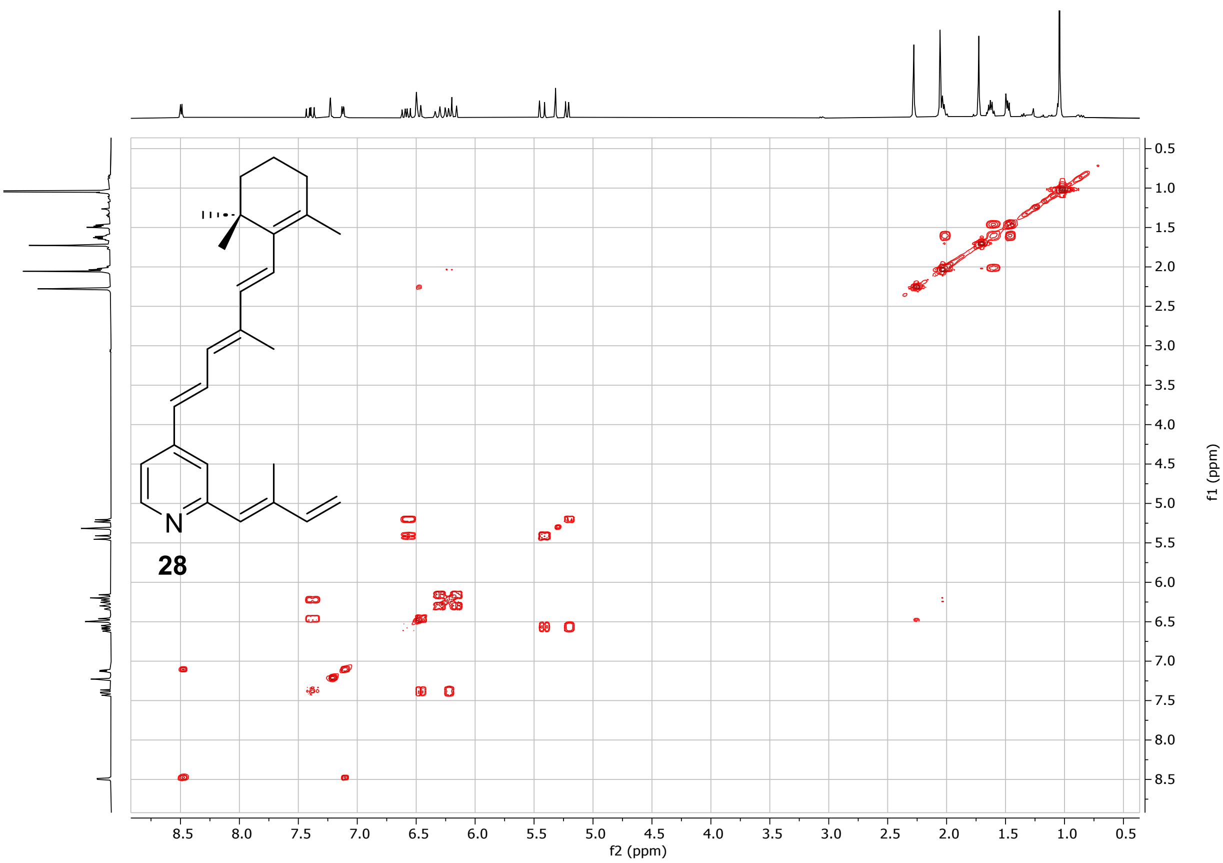

HSQC (CD_2Cl_2)

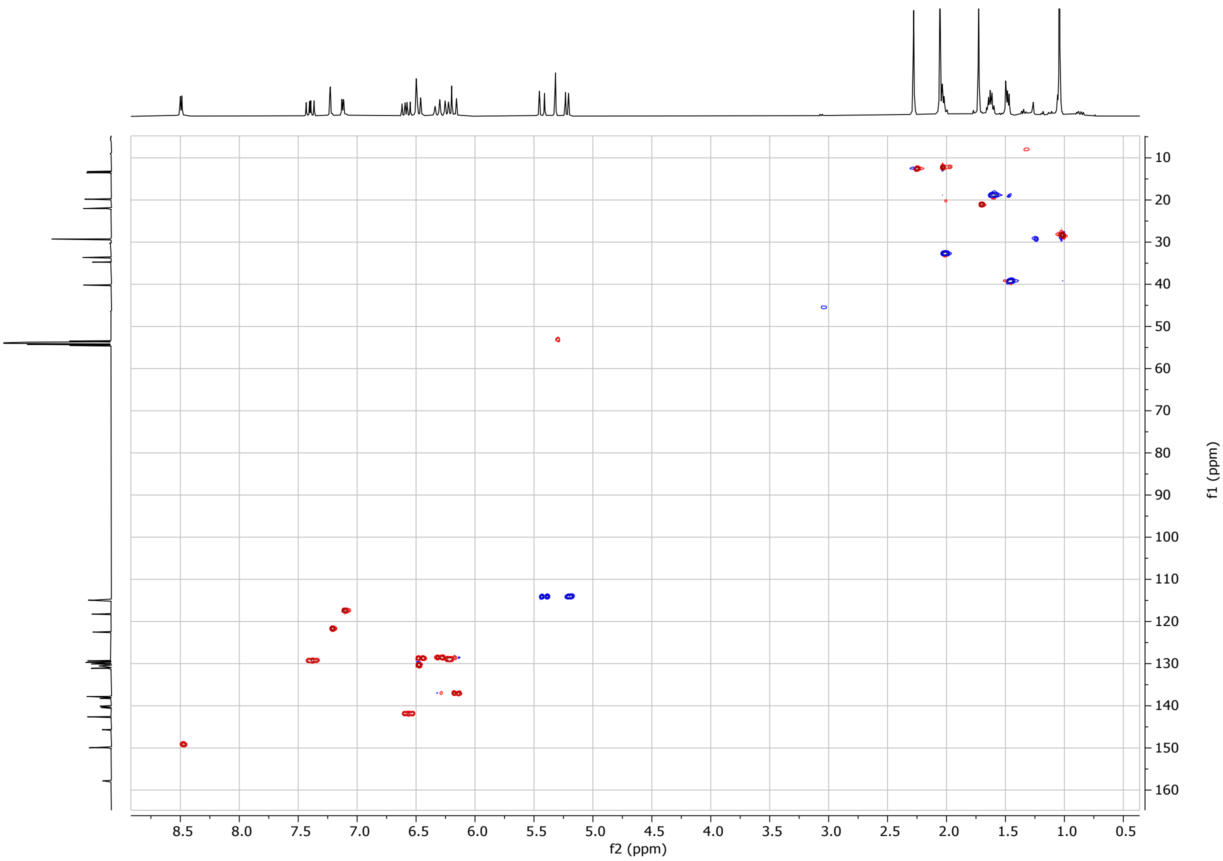

HMBC (CD_2Cl_2)

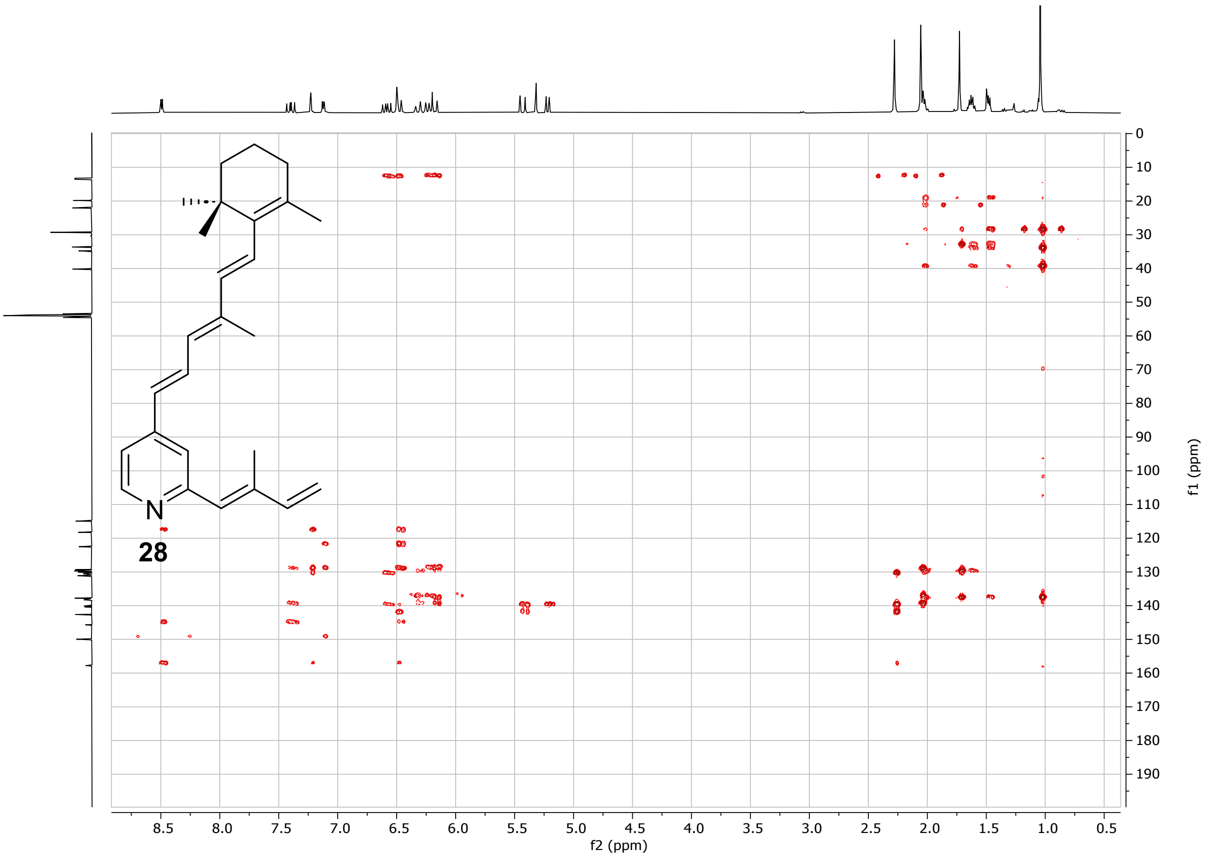

^{1}H-NMR (400.16 MHz, CD_3OD)

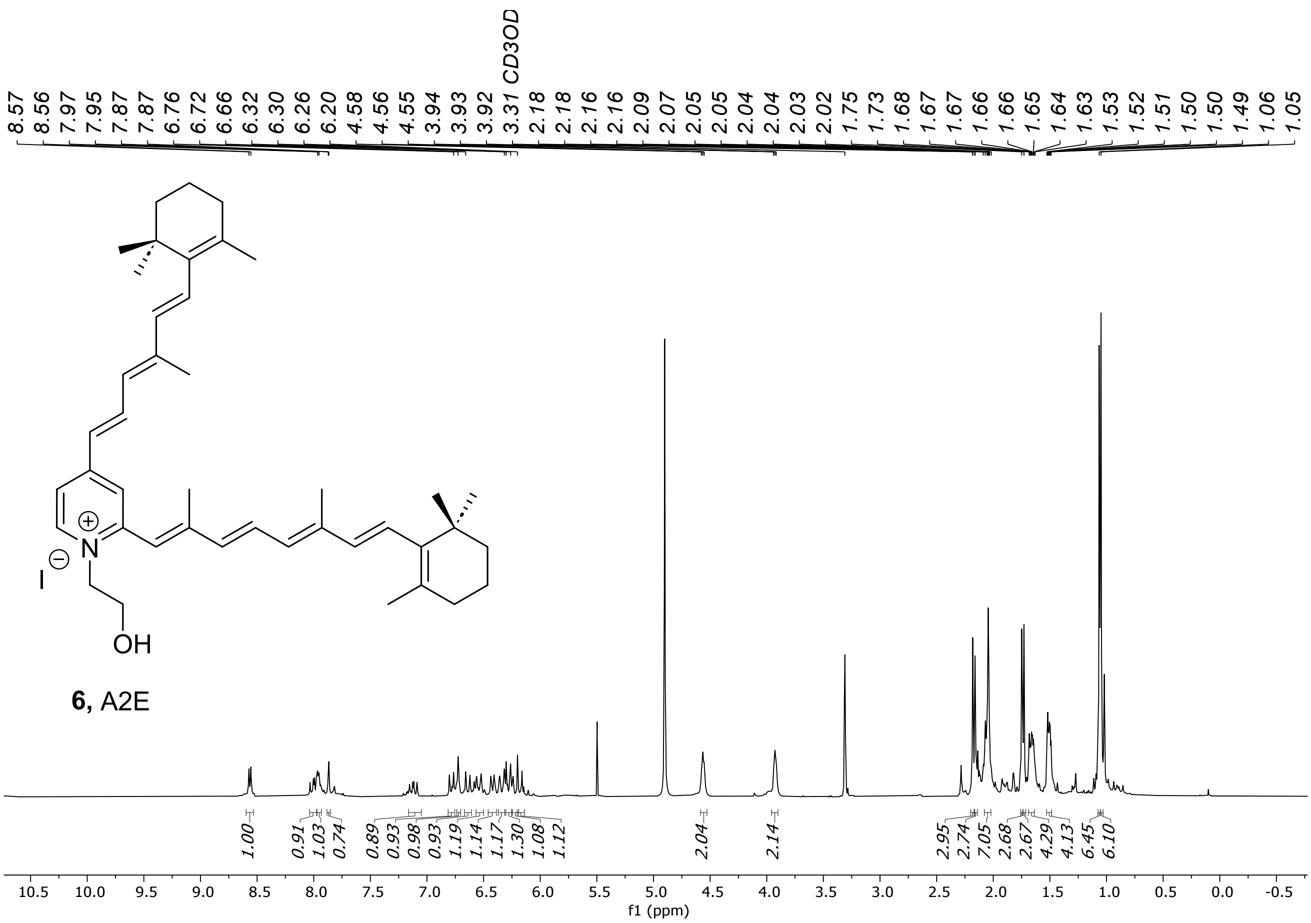

^{1}H-NMR (400.16 MHz, C_6D_6)

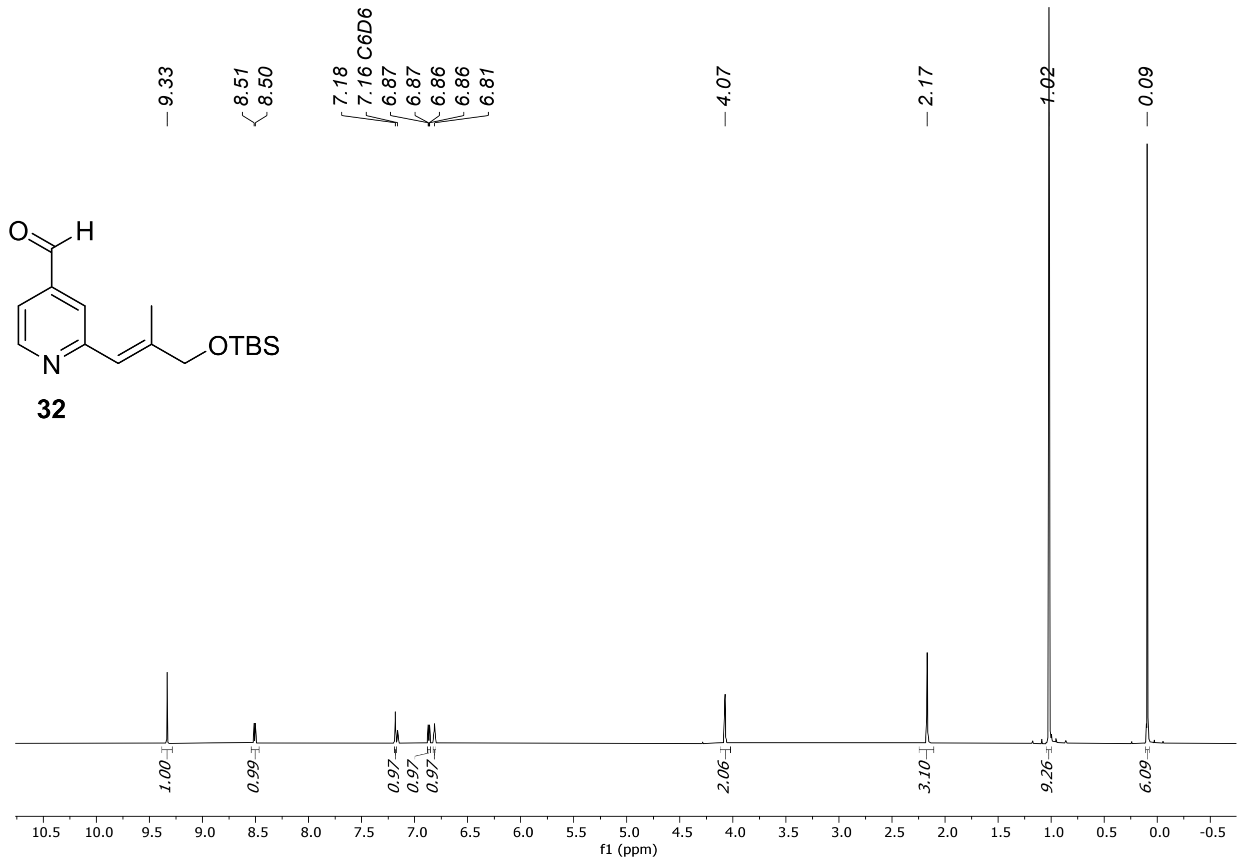

^{13}C{^{1}H}-NMR (100.63 MHz, C_6D_6)

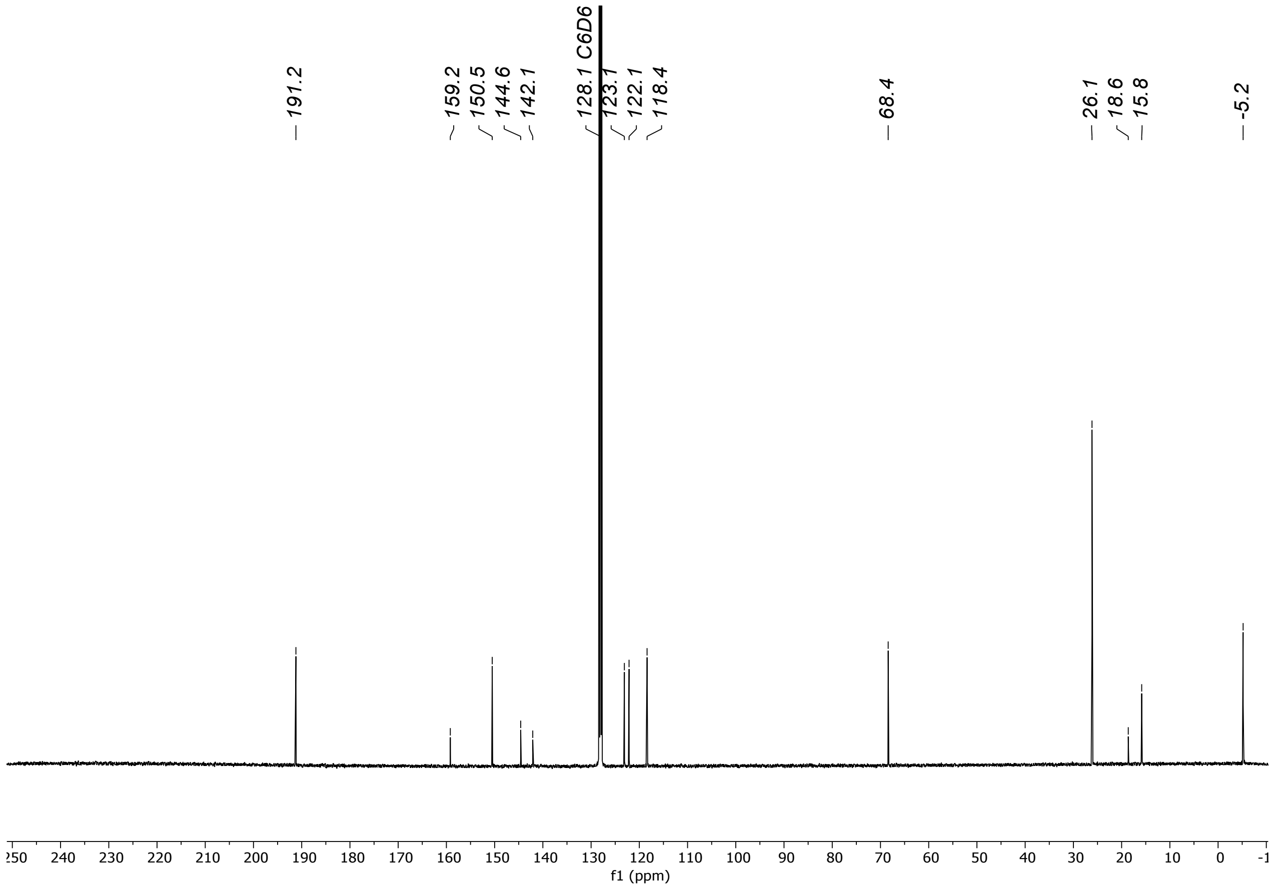

COSY (C_6D_6)

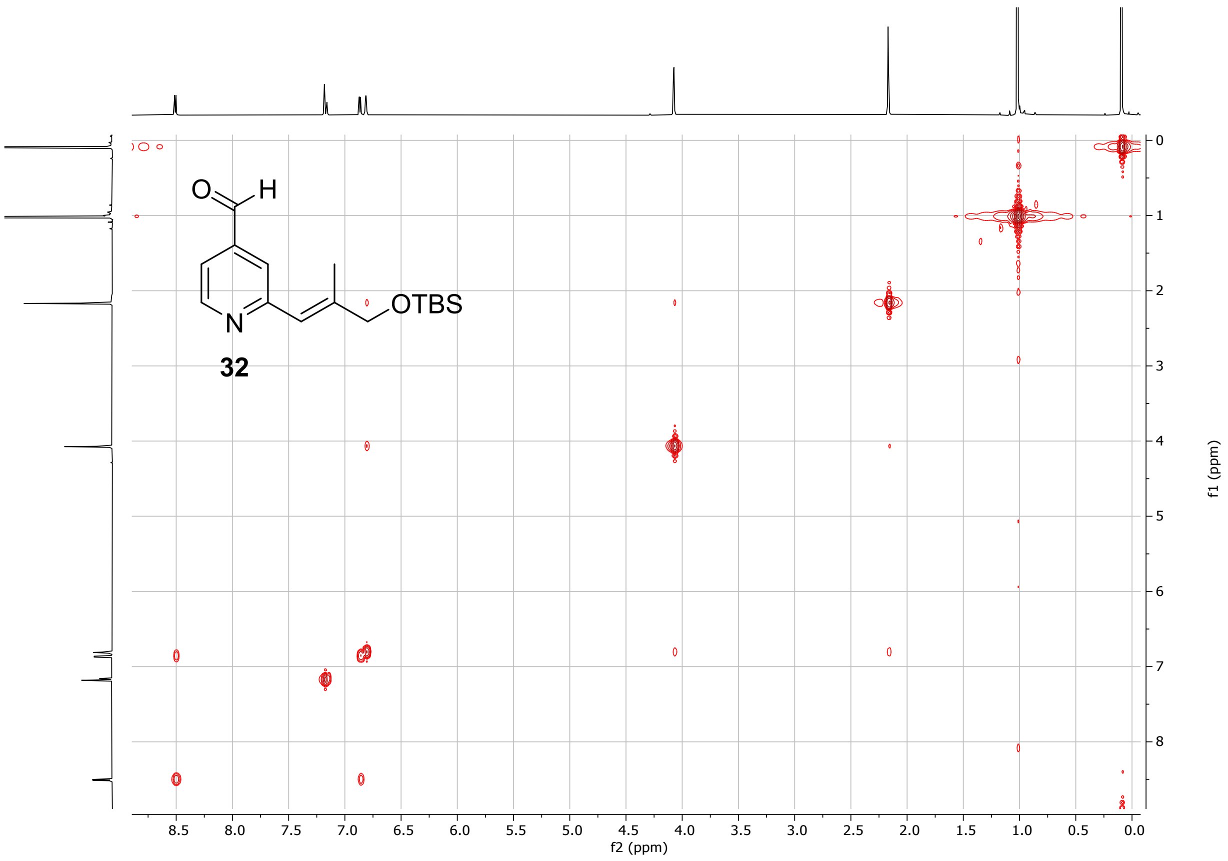

^{1}H-NMR (400.16 MHz, C_6D_6)

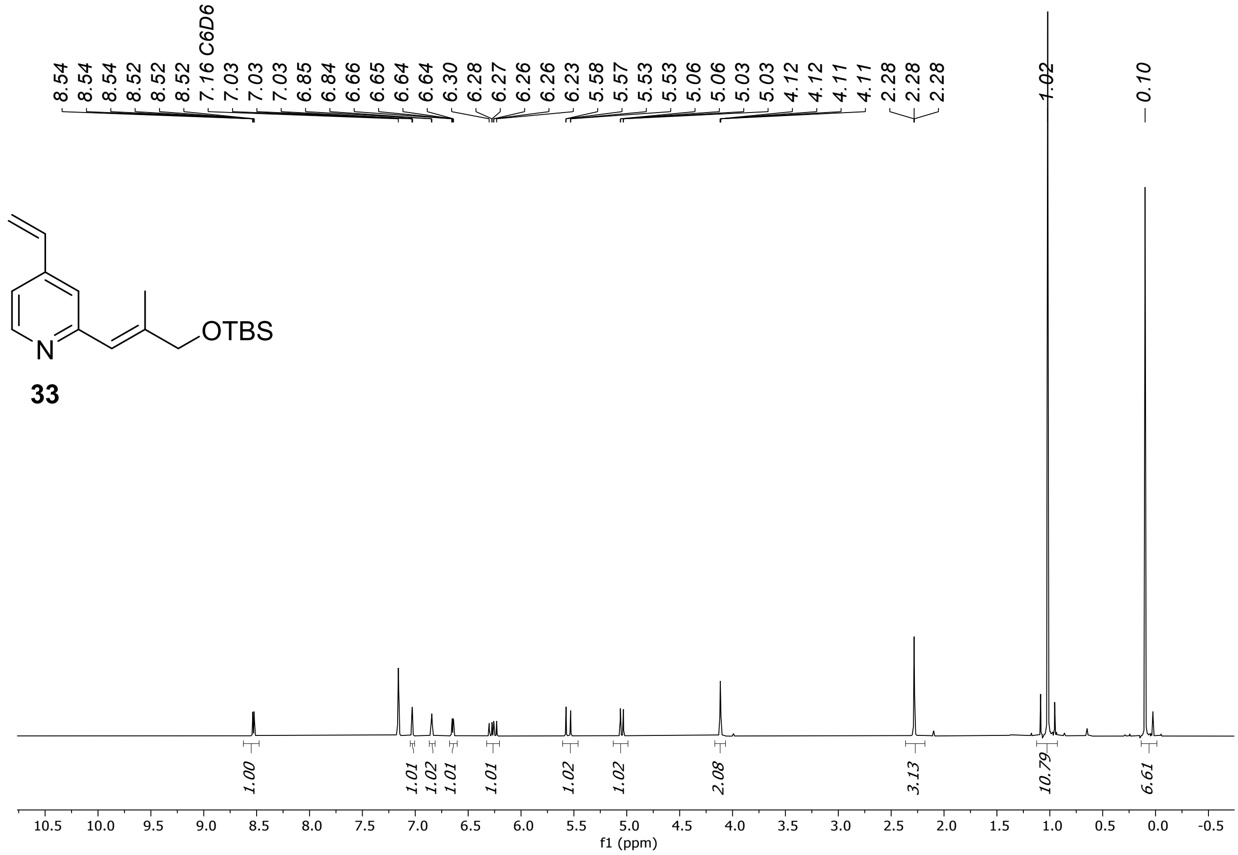

33

^{13}C{^{1}H}-NMR (100.63 MHz, C_6D_6)

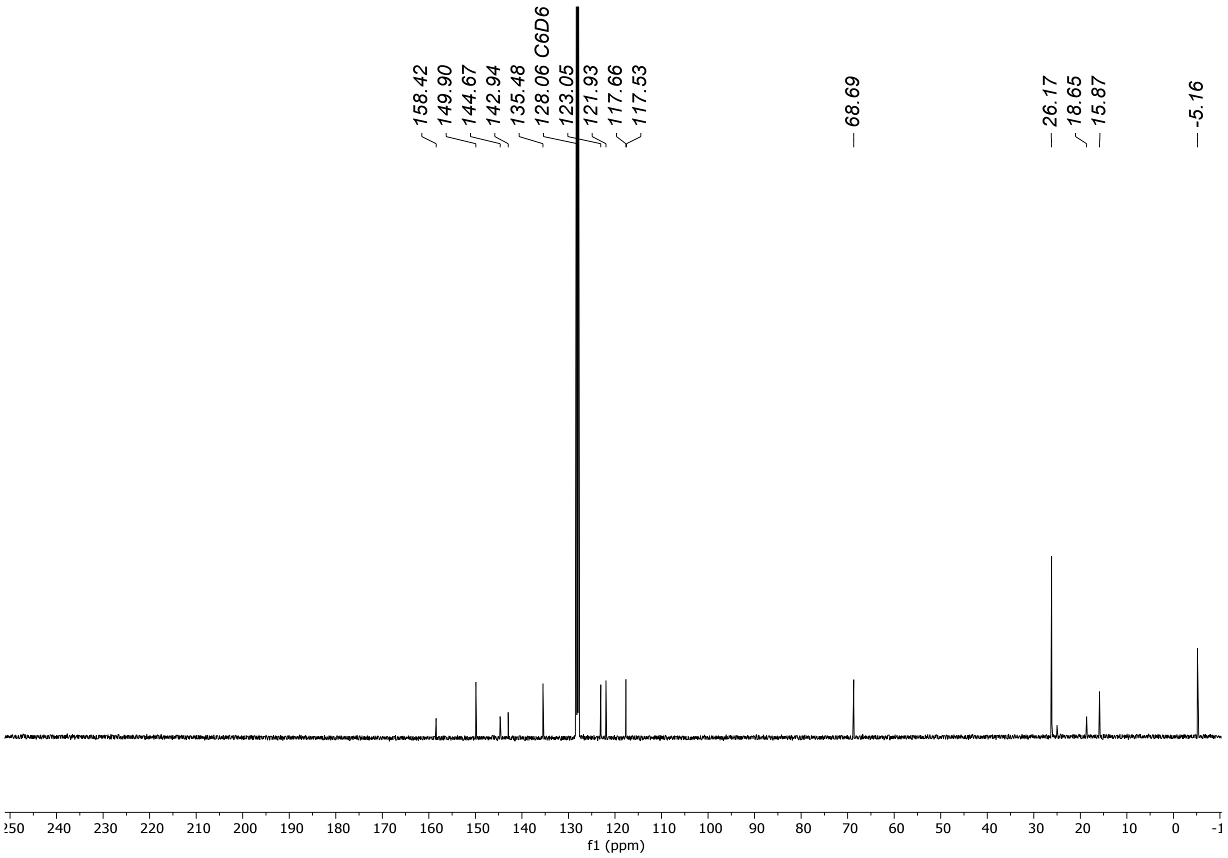

COSY (C_6D_6)

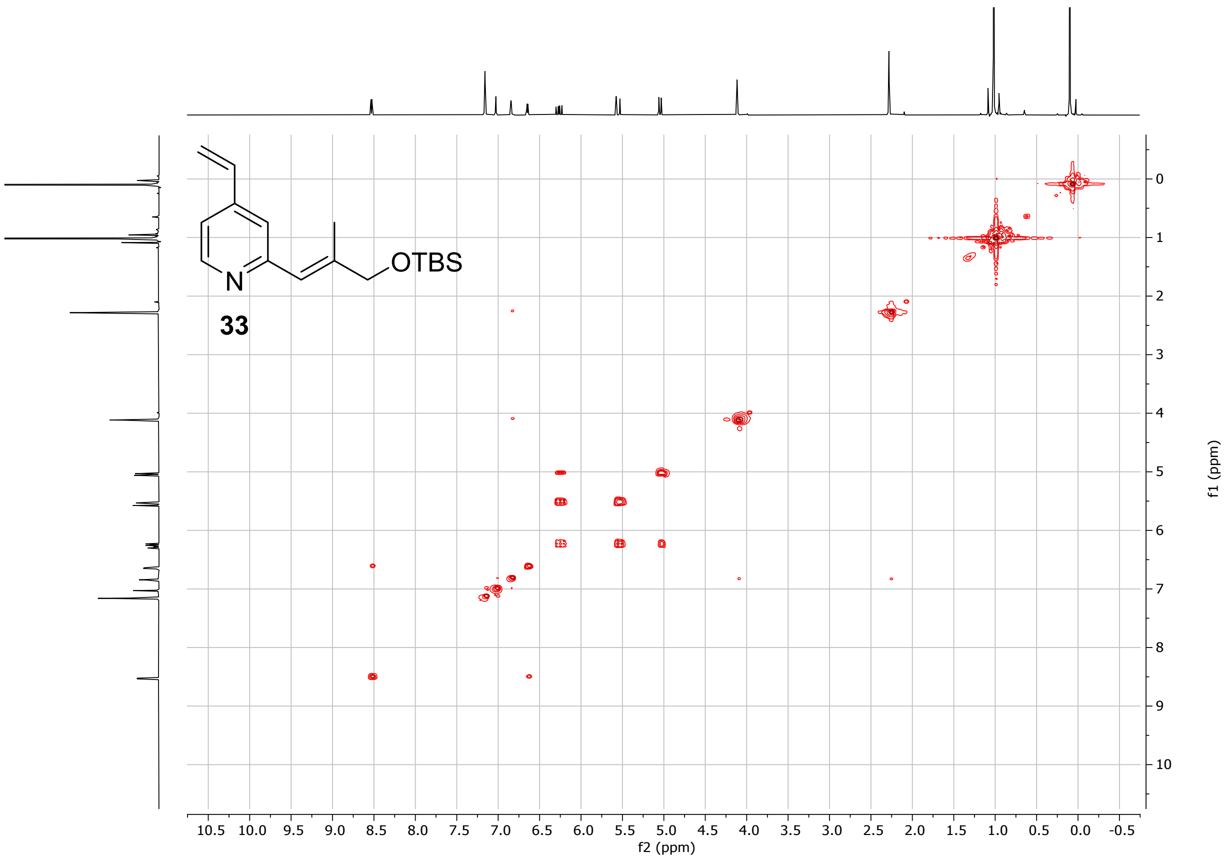

HSQC (C_6D_6)

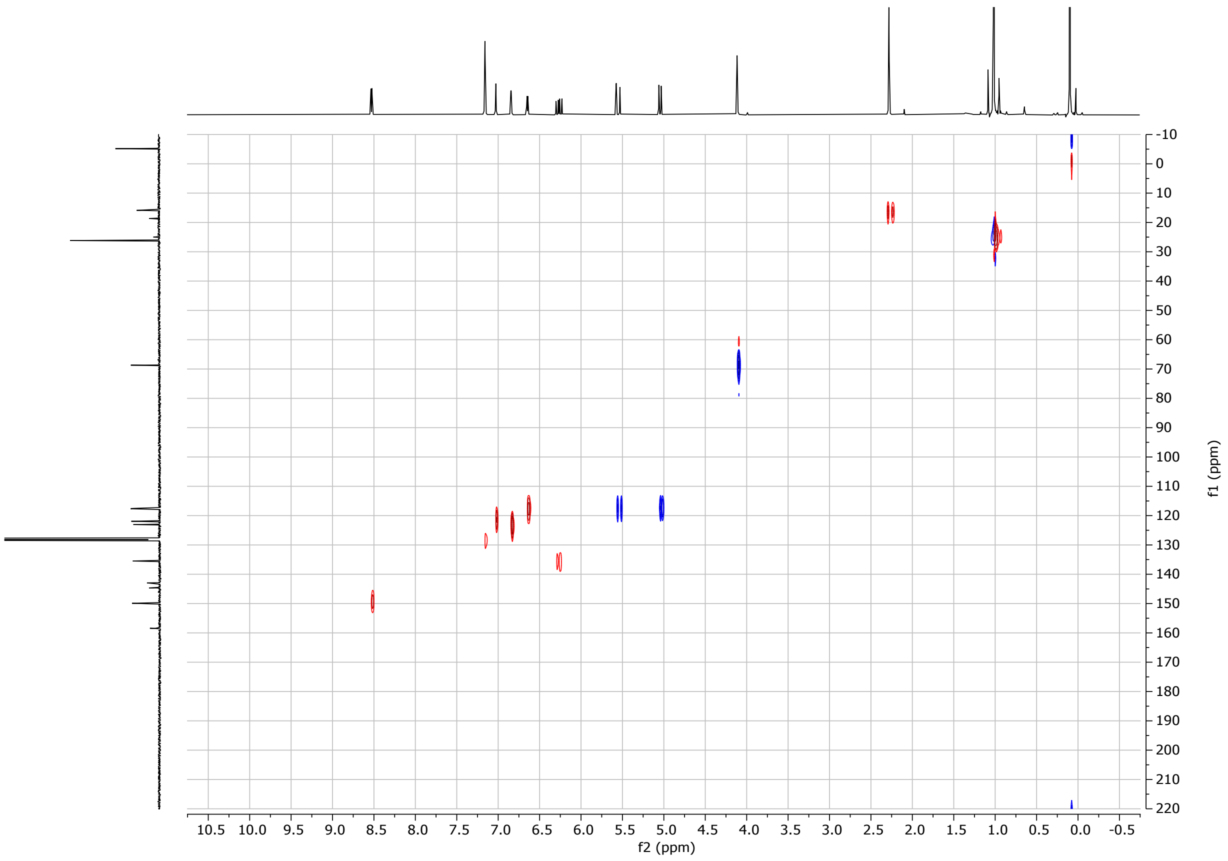

HMBC (C_6D_6)

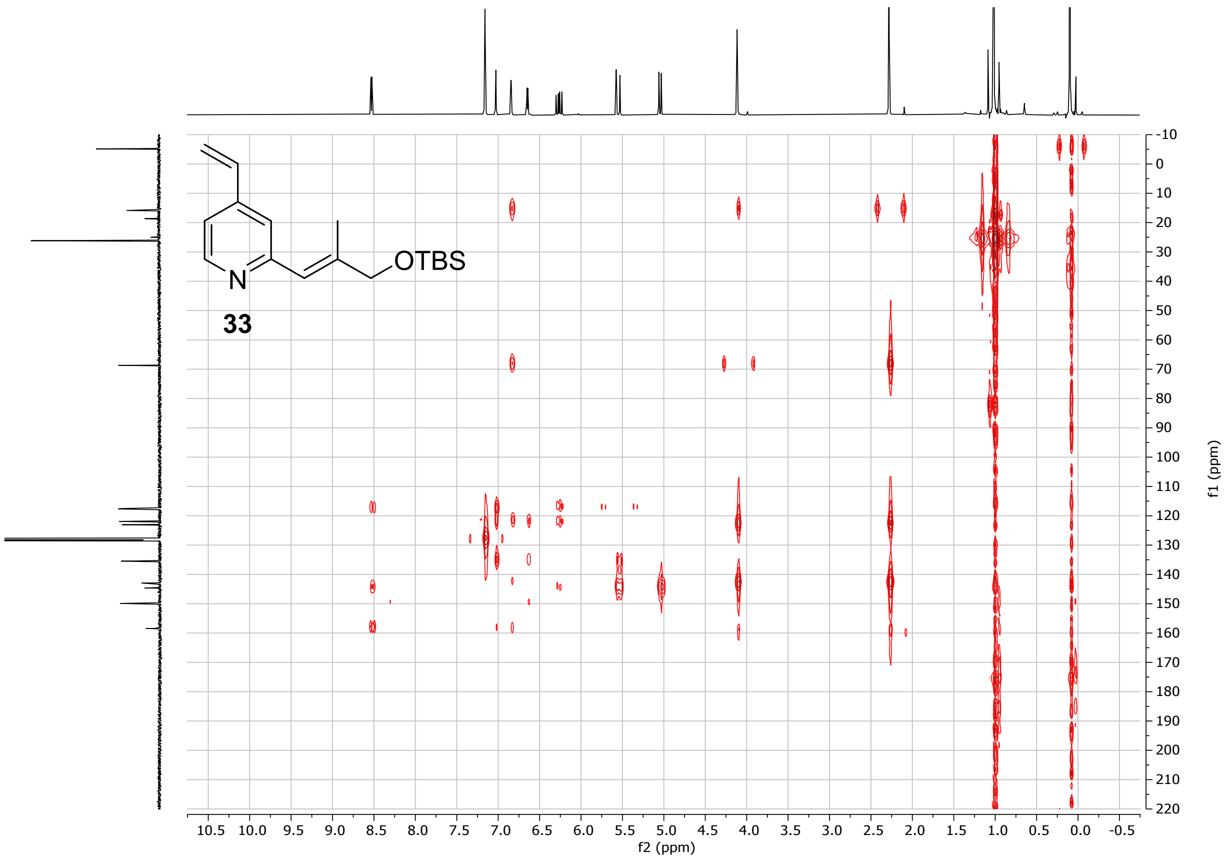

^{1}H-NMR (400.16 MHz, C_6D_6)

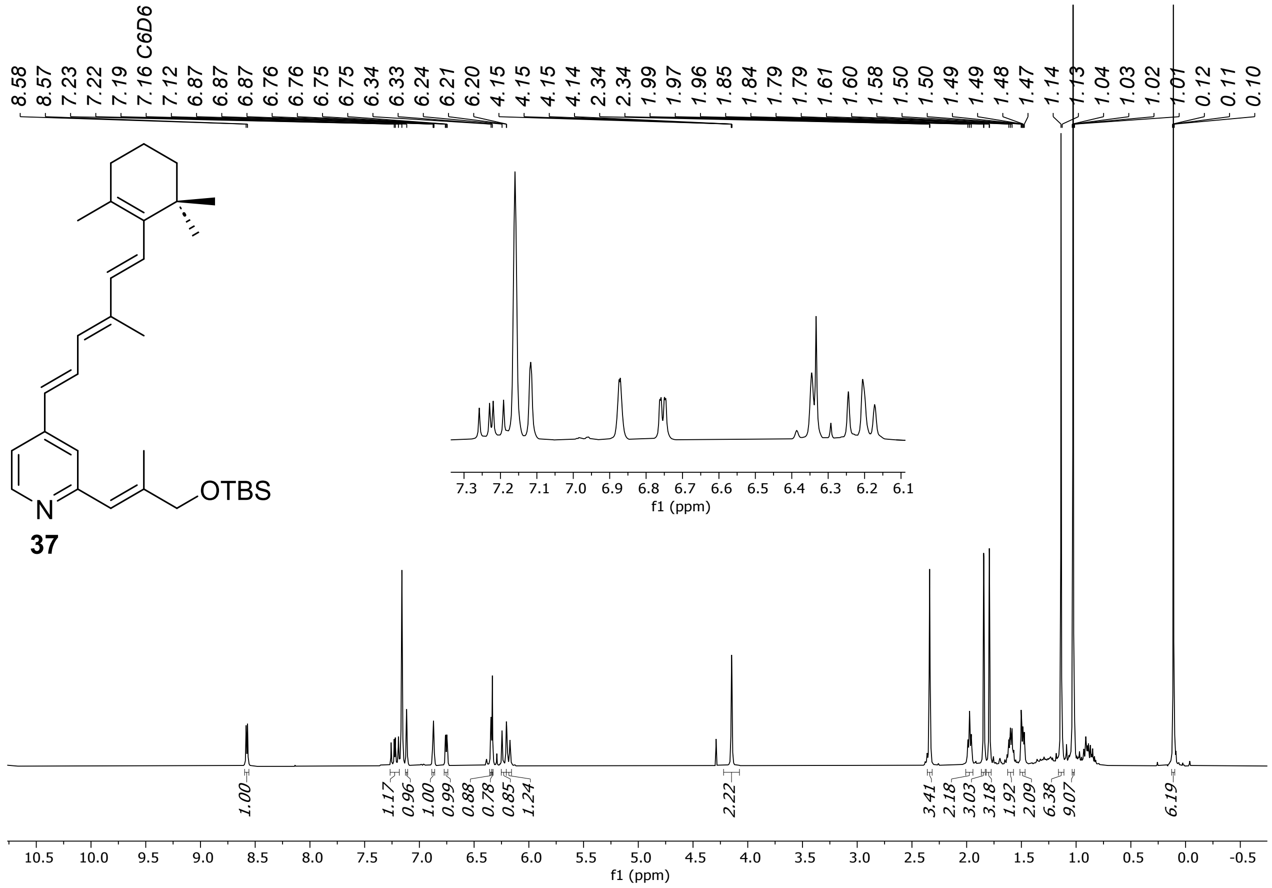

^{13}C{^{1}H}-NMR (100.63 MHz, C_6D_6)

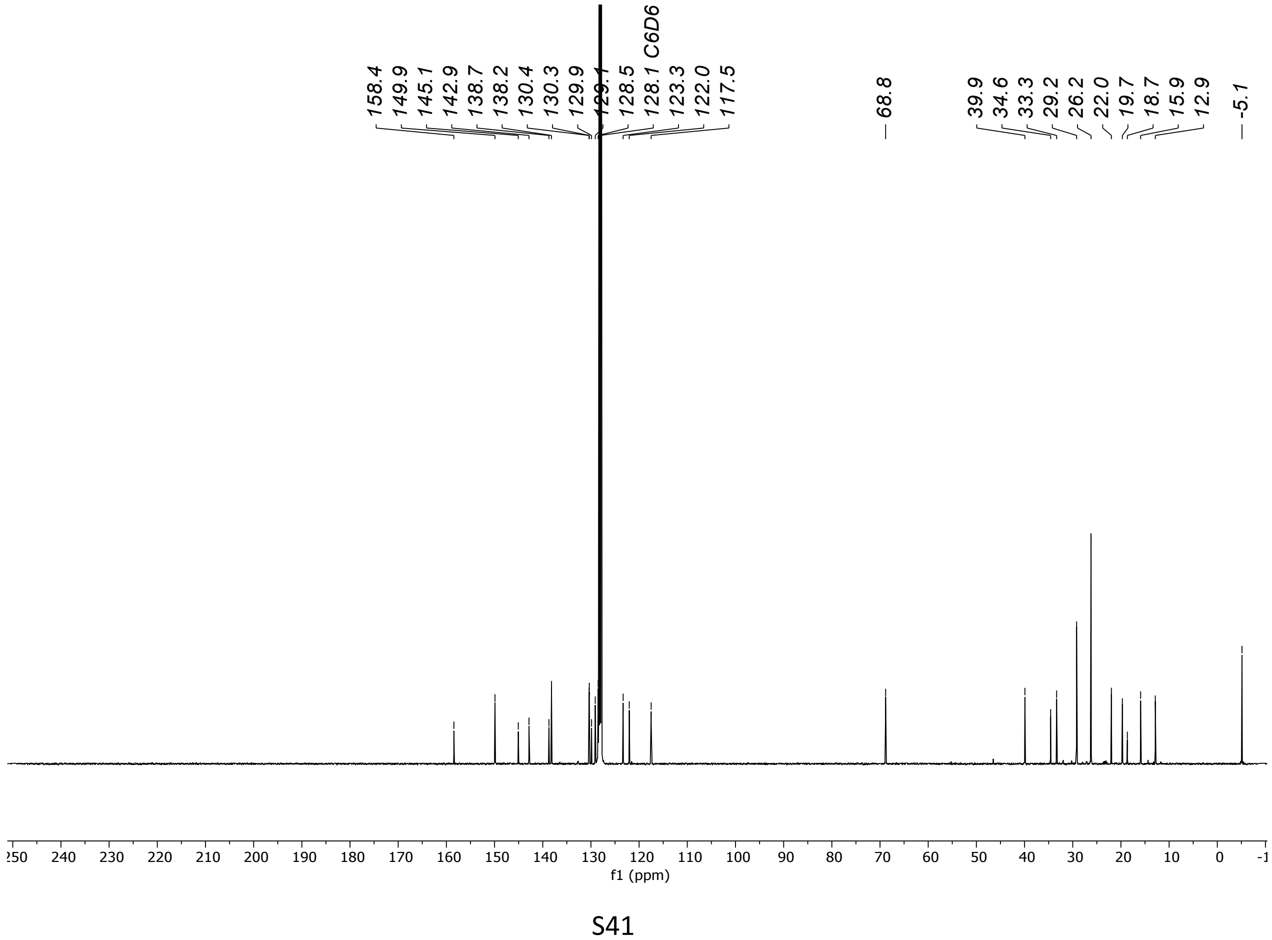

COSY (C_6D_6)

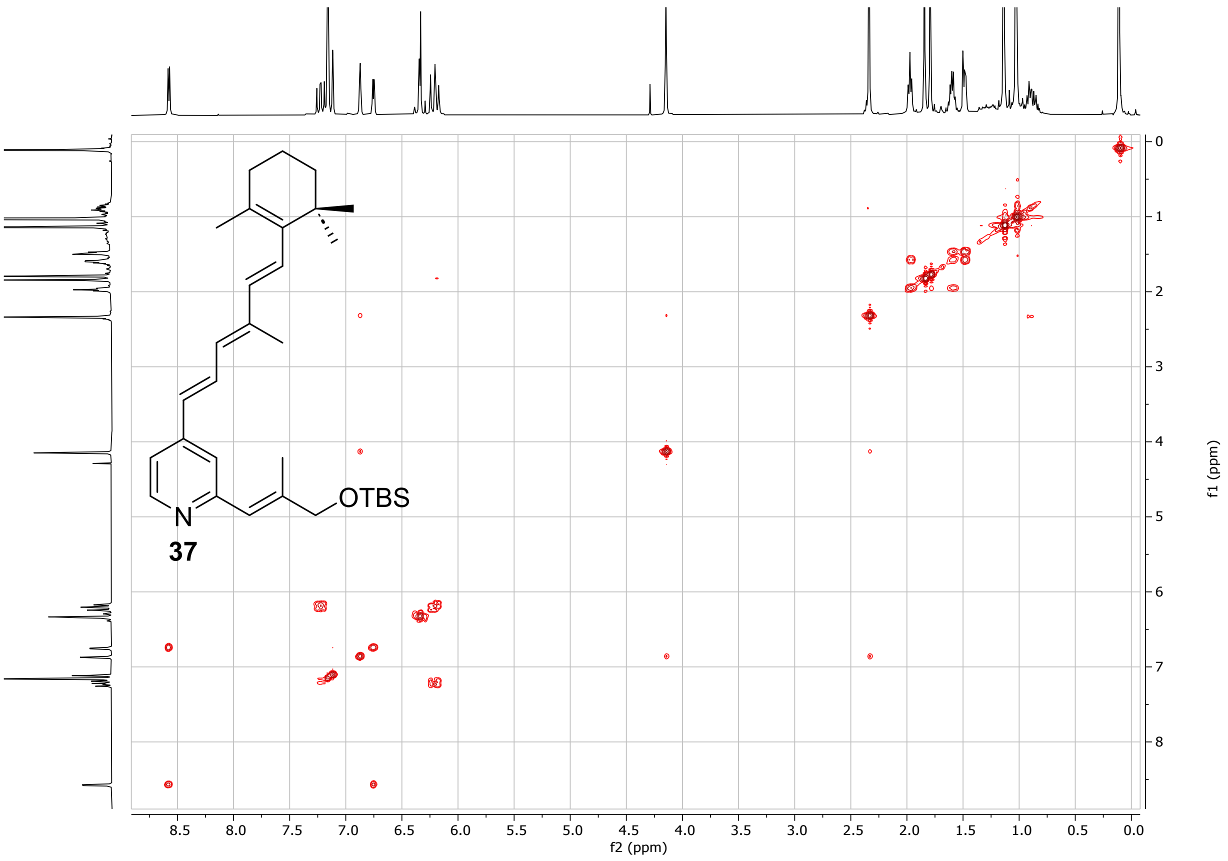

HSQC (C_6D_6)

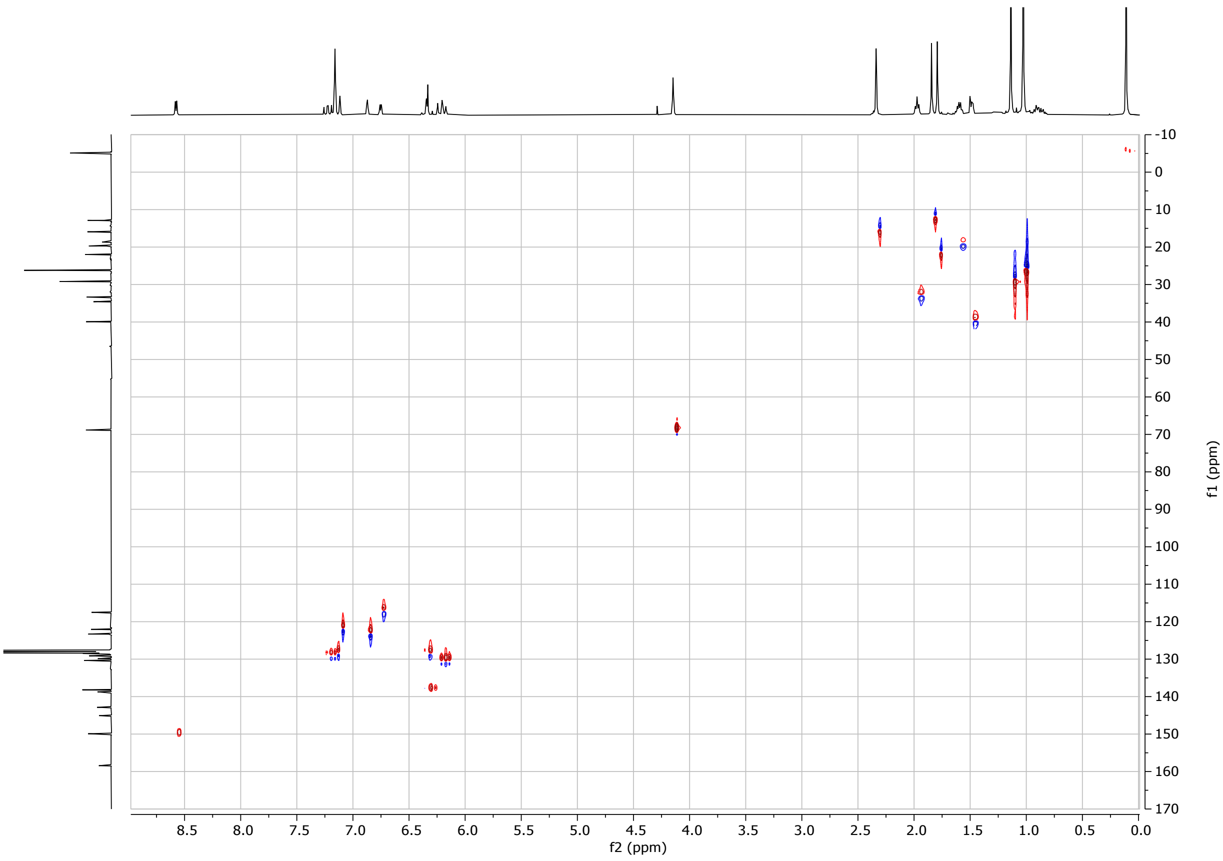

HMBC (C_6D_6)

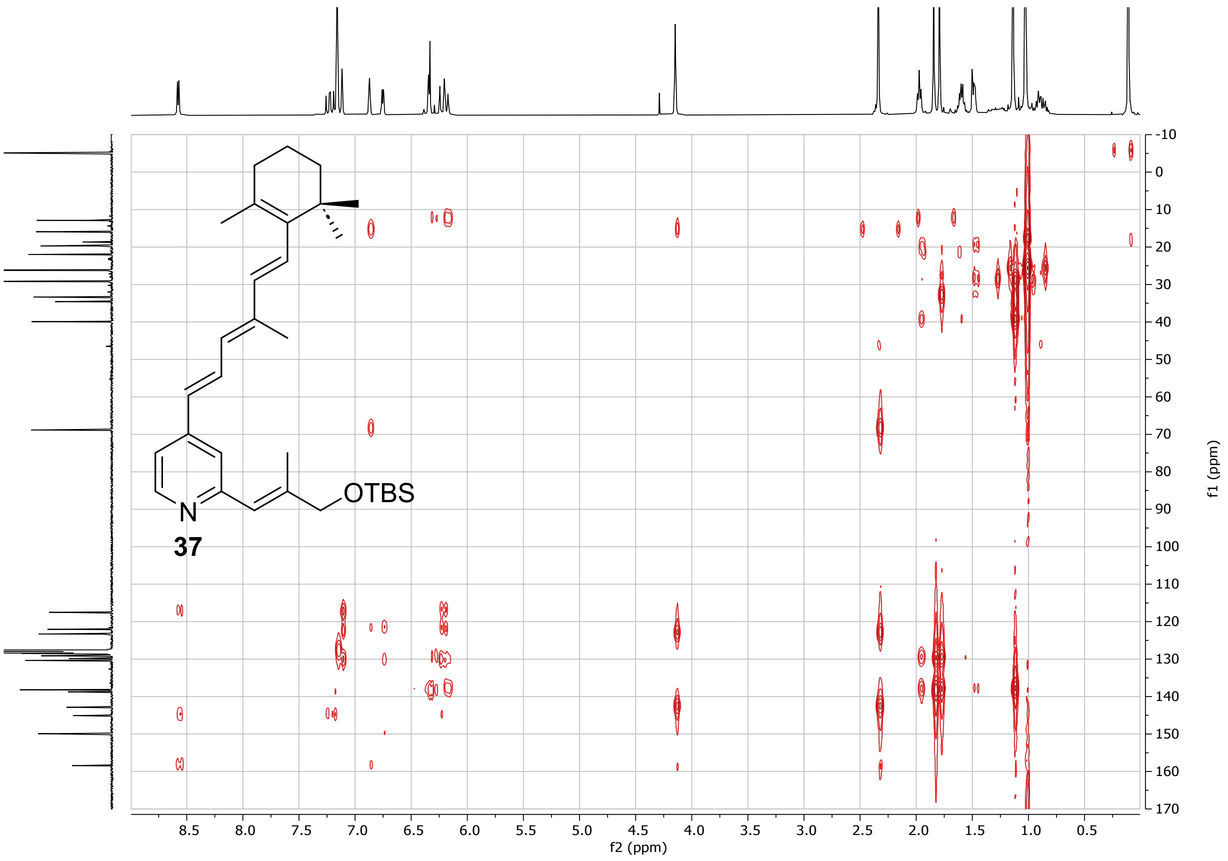

^{1}H-NMR (400.16 MHz, CD_2Cl_2)

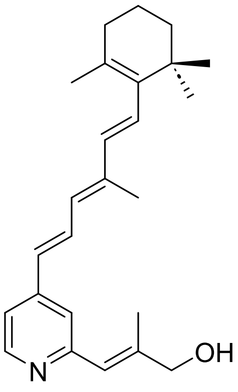

38

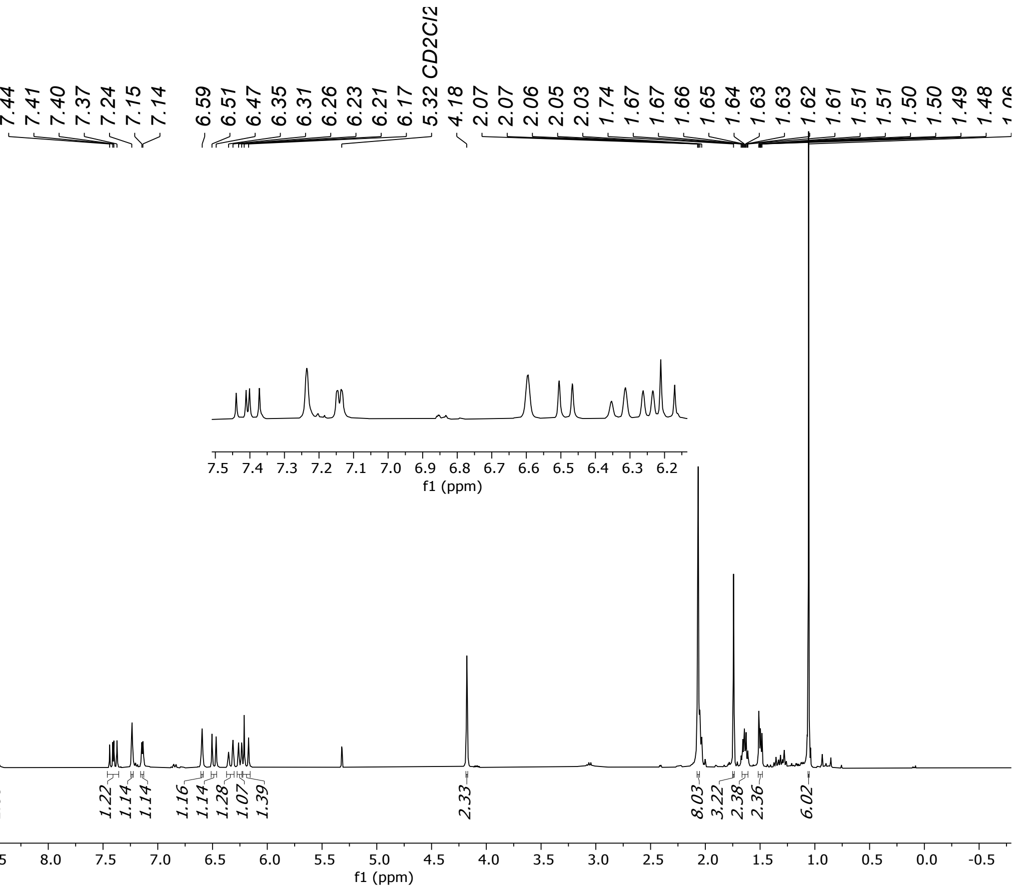

$^{13}C\{^{1}H\}$-NMR (100.63 MHz, CD_2Cl_2)

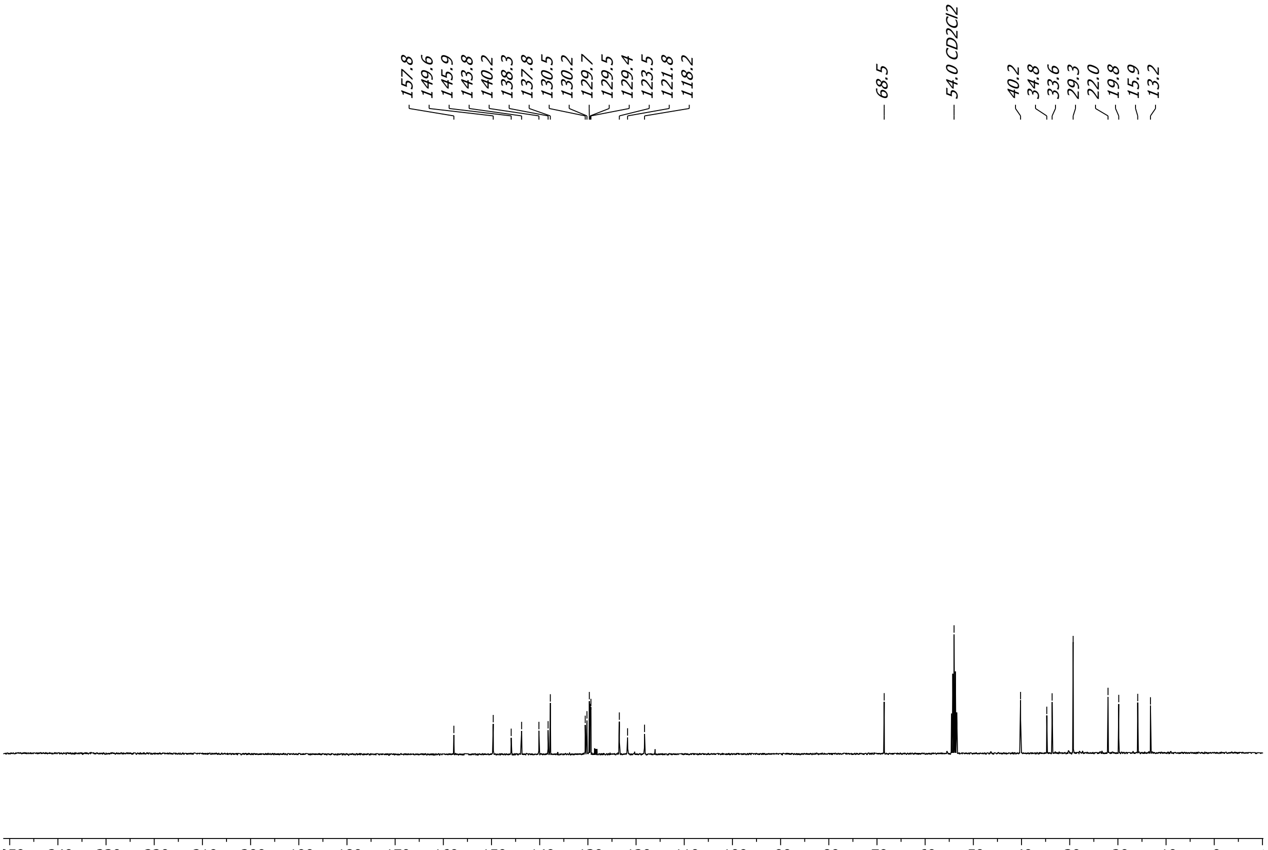

COSY (CD_2Cl_2)

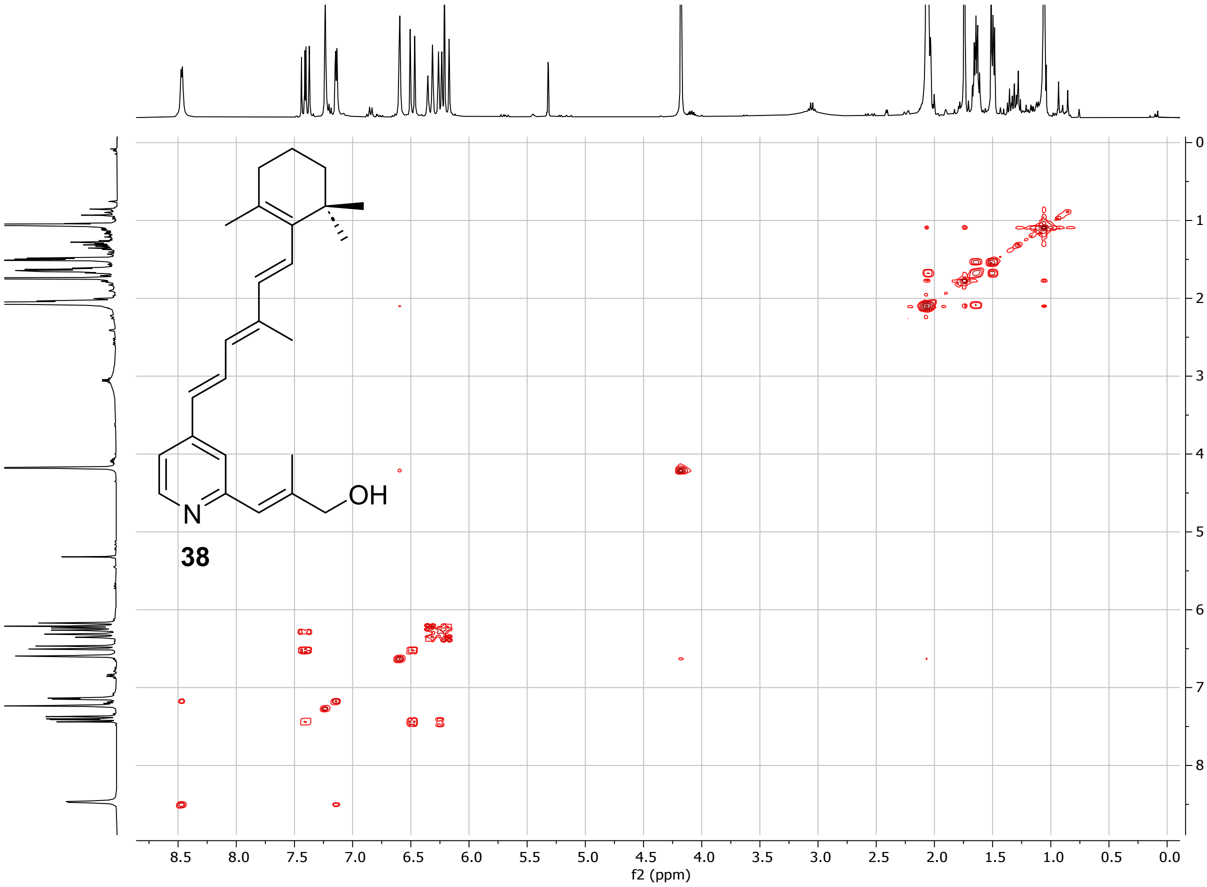

HSQC (CD_2Cl_2)

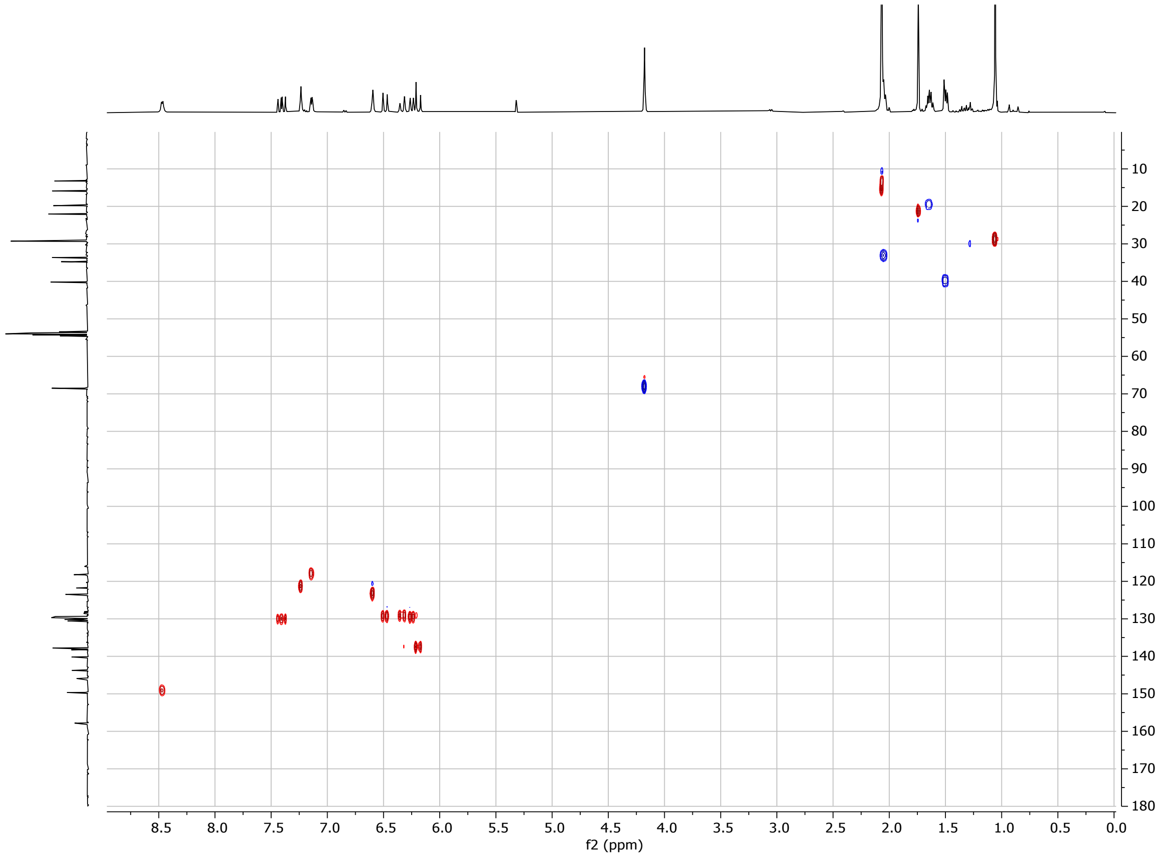

HMBC (CD_2Cl_2)

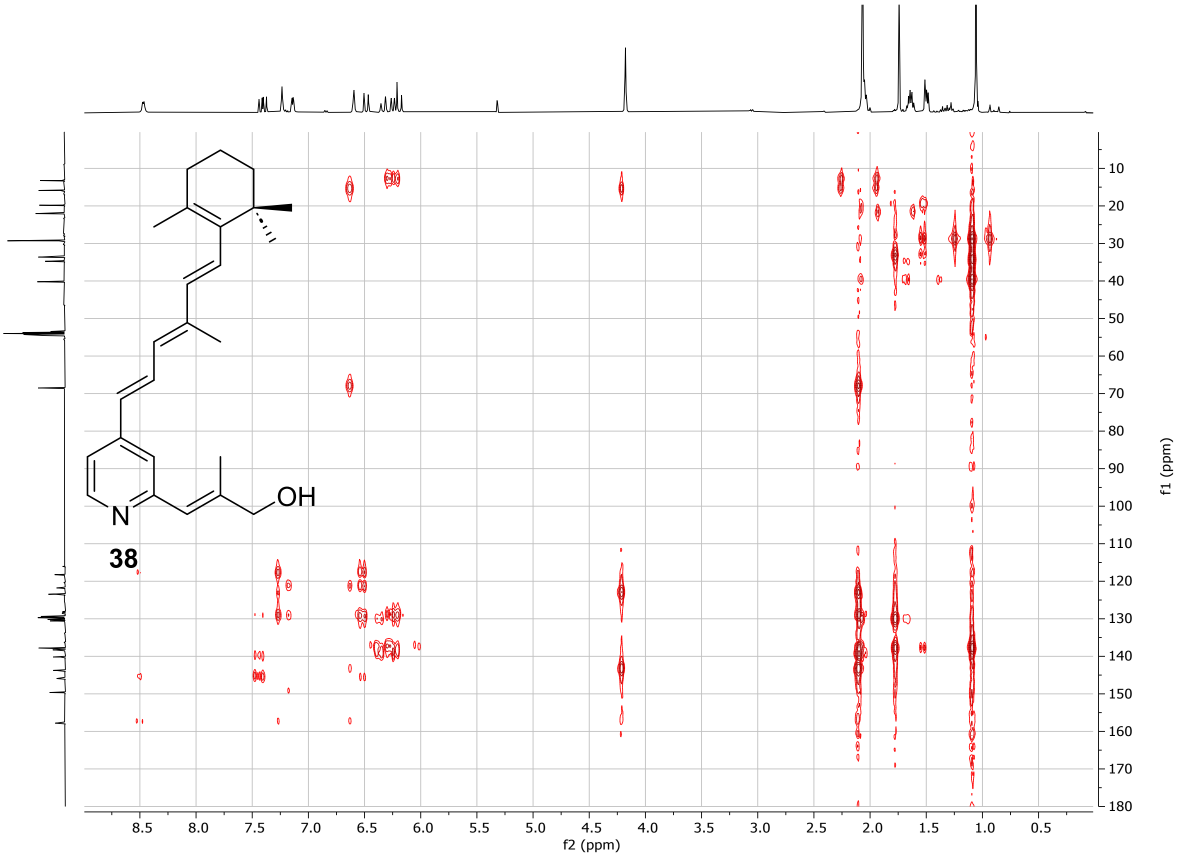

^{1}H-NMR (400.16 MHz, C_6D_6)

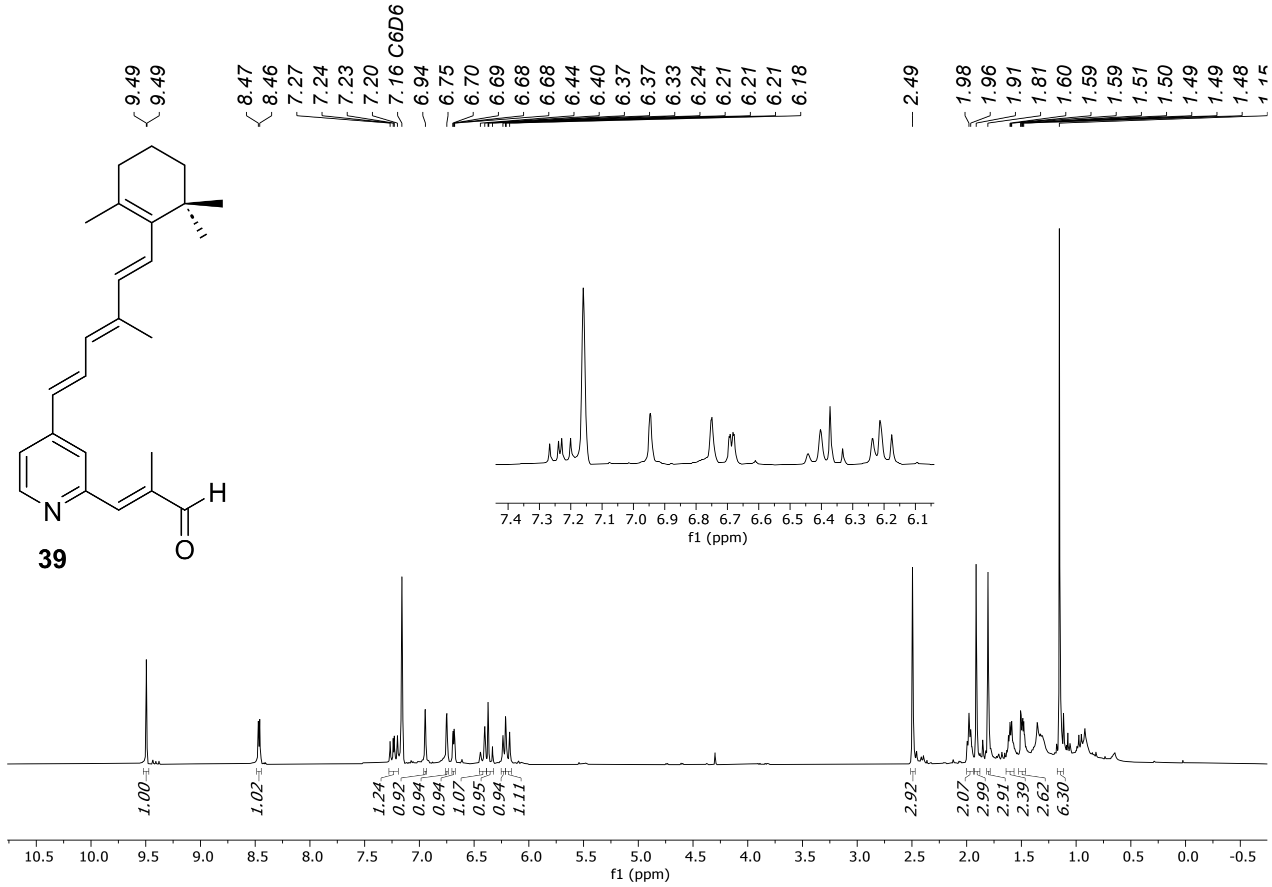

39

^{13}C{^{1}H}-NMR (100.63 MHz, C_6D_6)

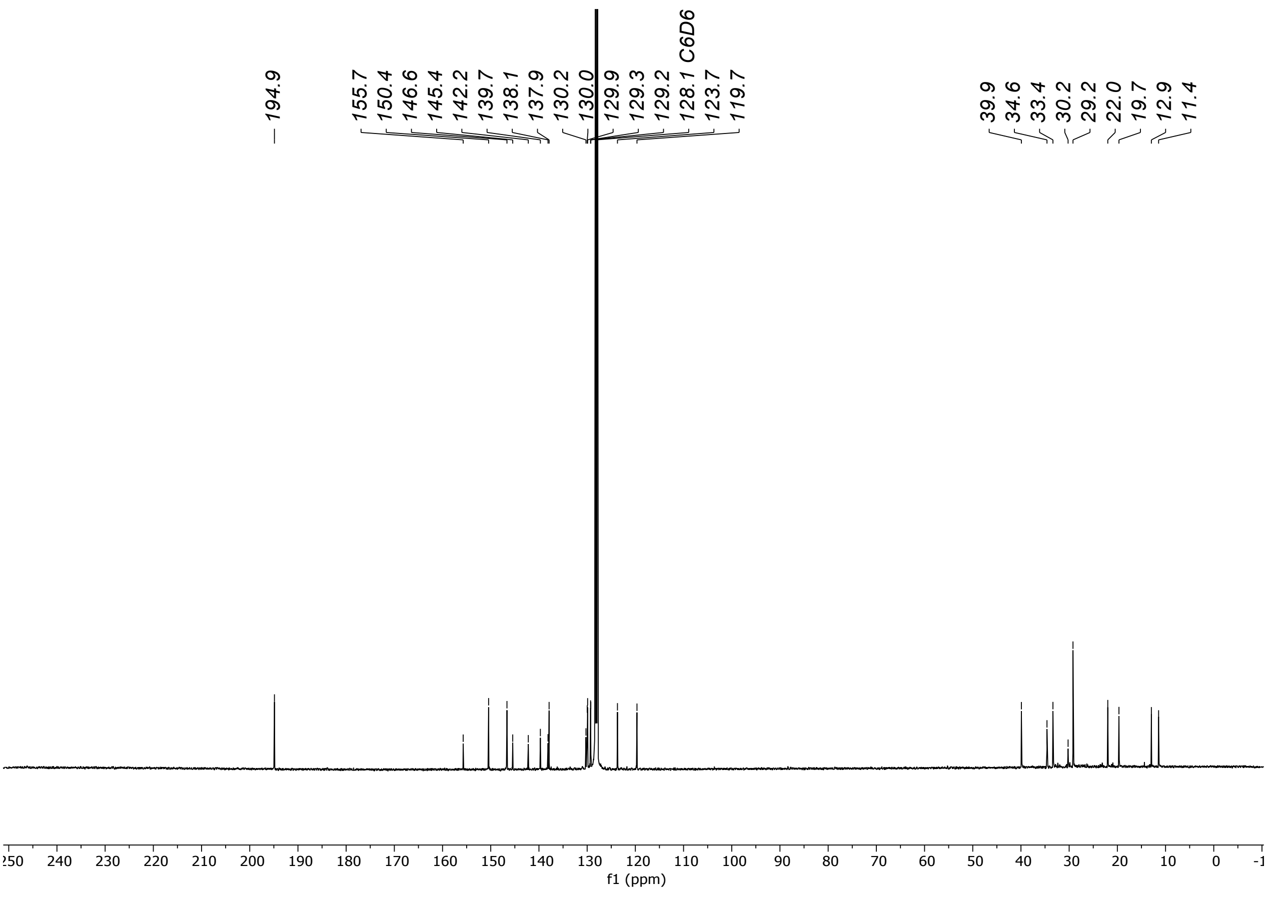

COSY (C_6D_6)

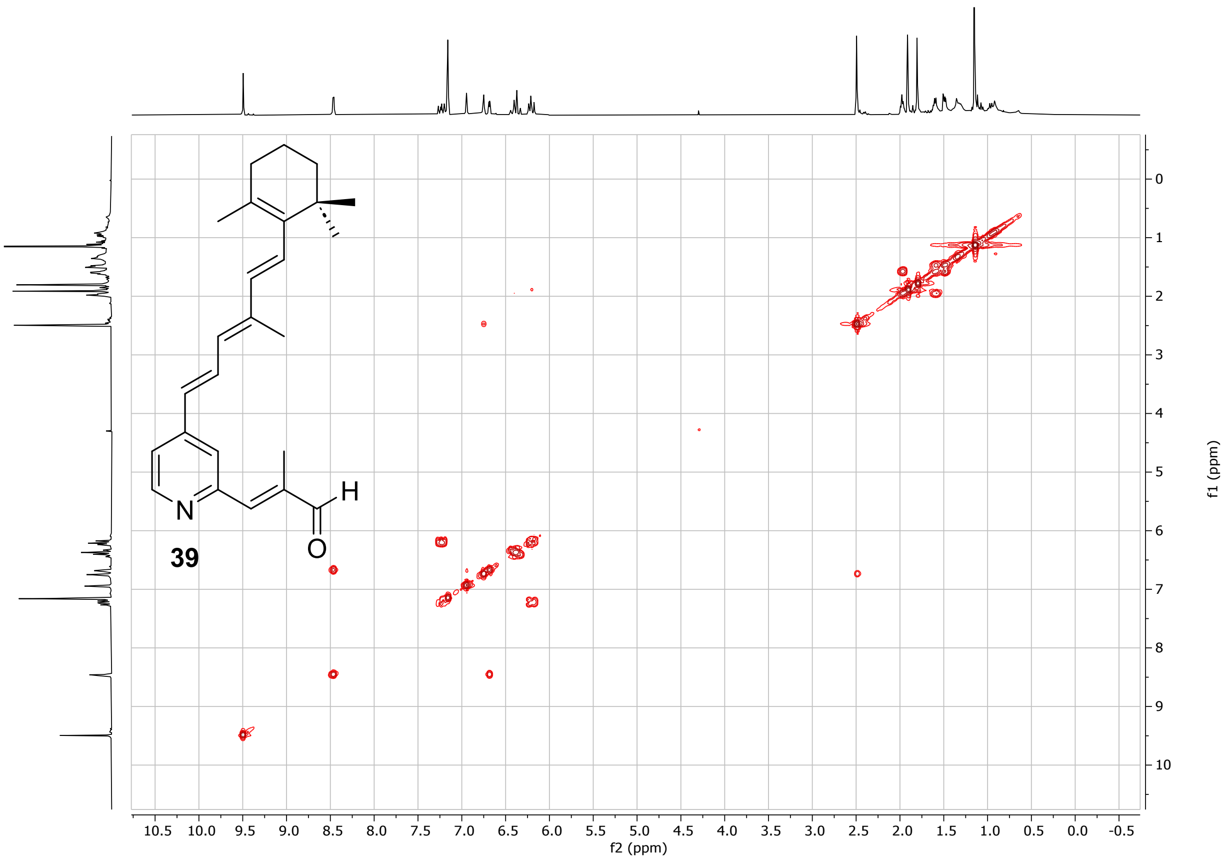

HSQC (C_6D_6)

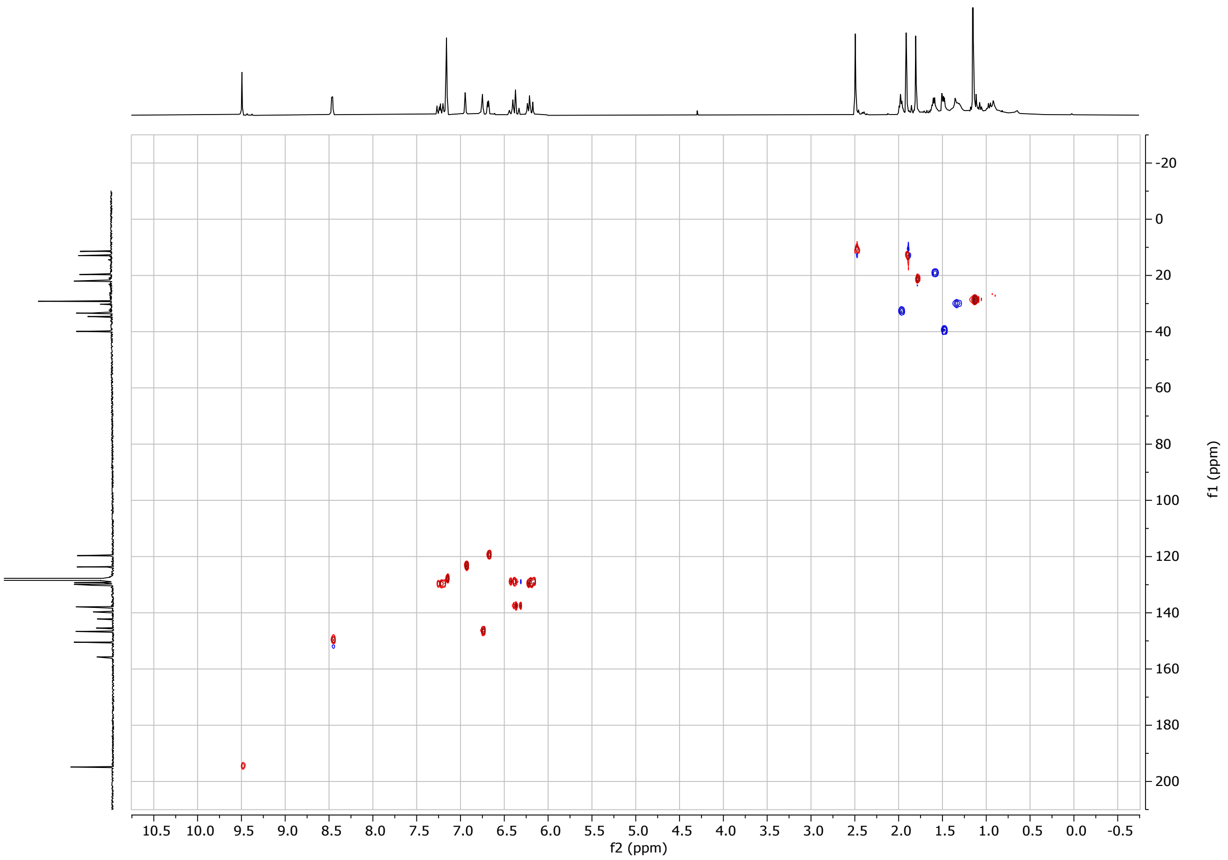

HMBC (C_6D_6)

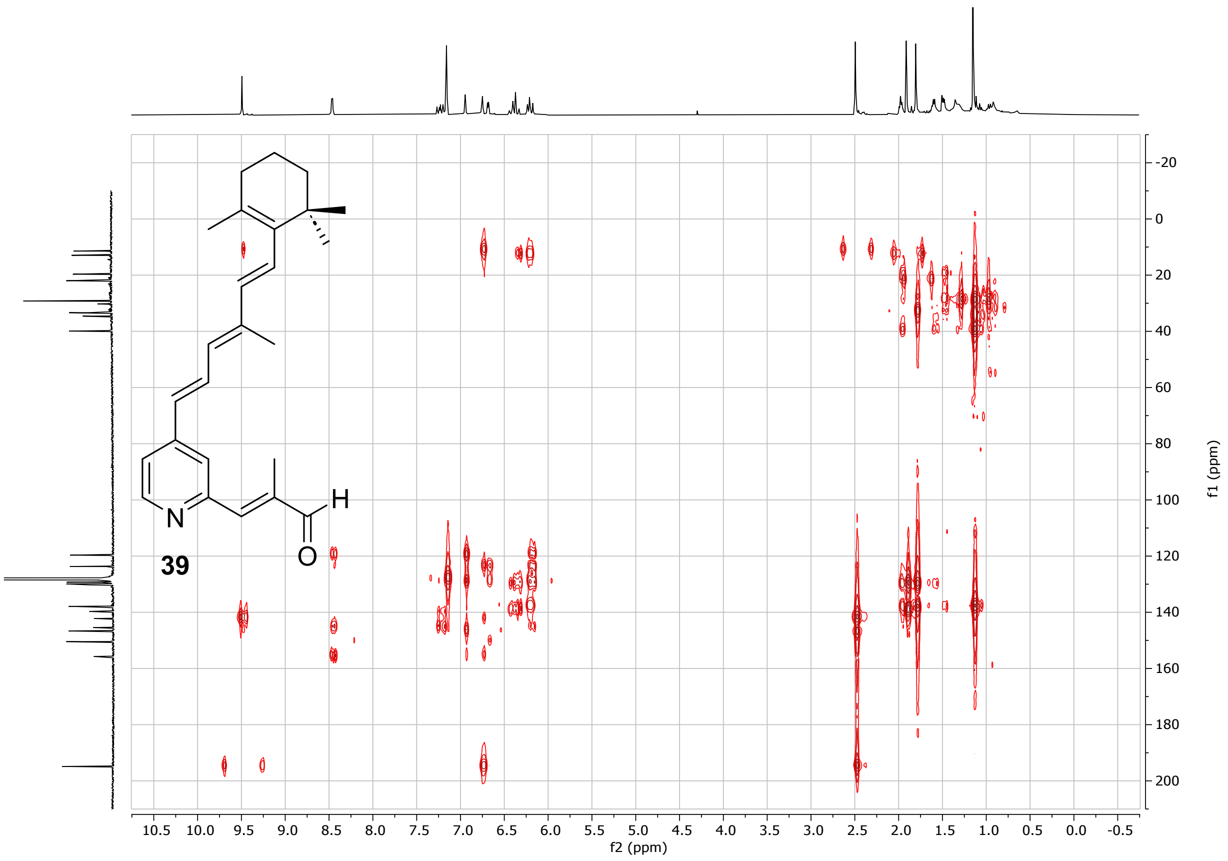

^{1}H-NMR (400.16 MHz, C_6D_6)

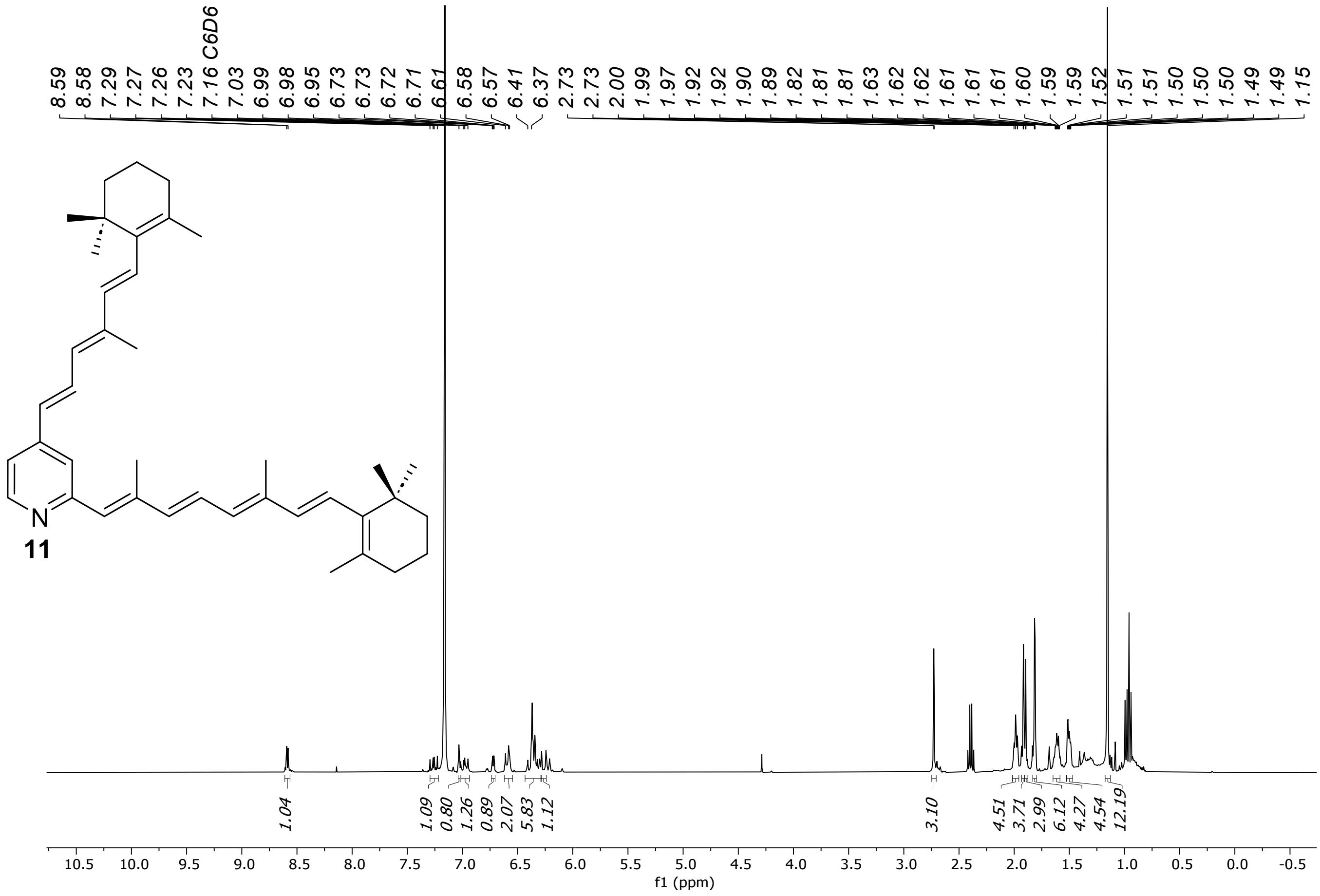

^{1}H-NMR (400.16 MHz, C_6D_6)

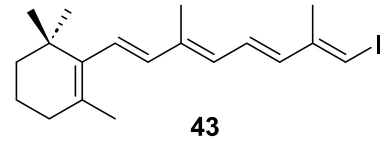
43

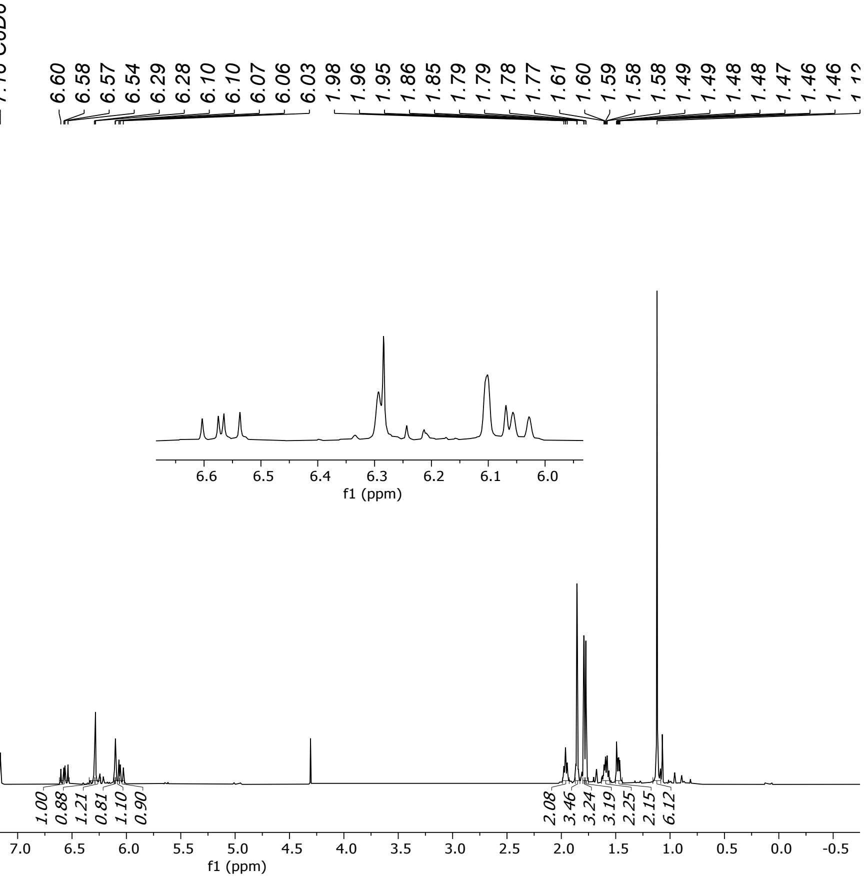

^{13}C{^{1}H}-NMR (100.63 MHz, C_6D_6)

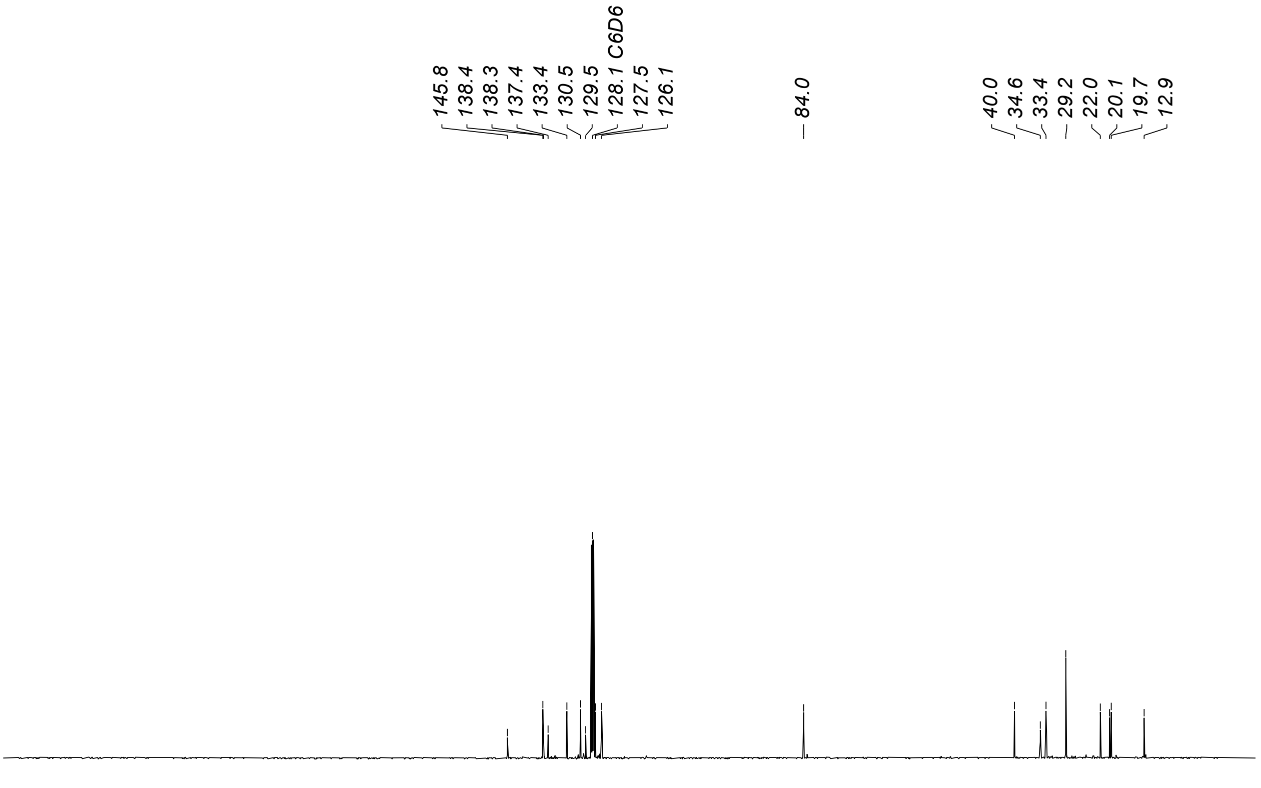

COSY (C_6D_6)

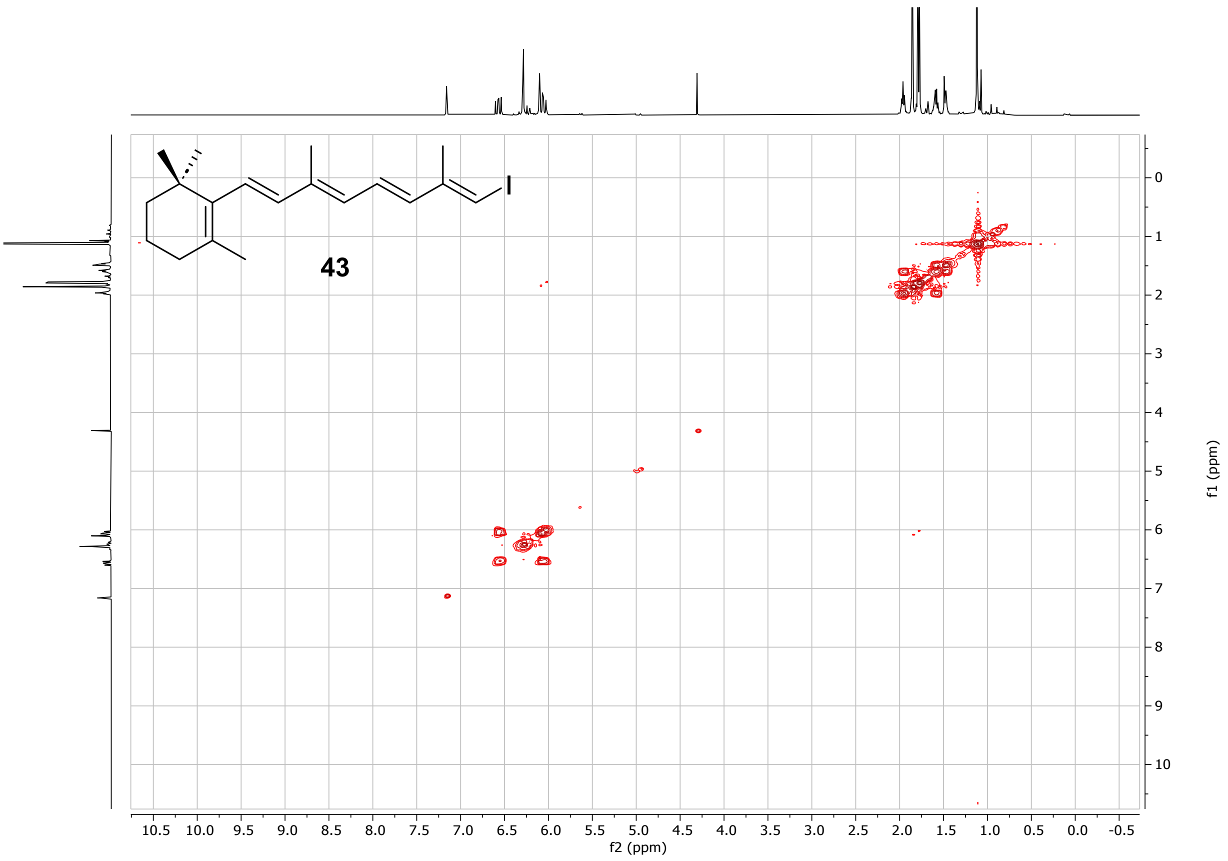

HSQC (C_6D_6)

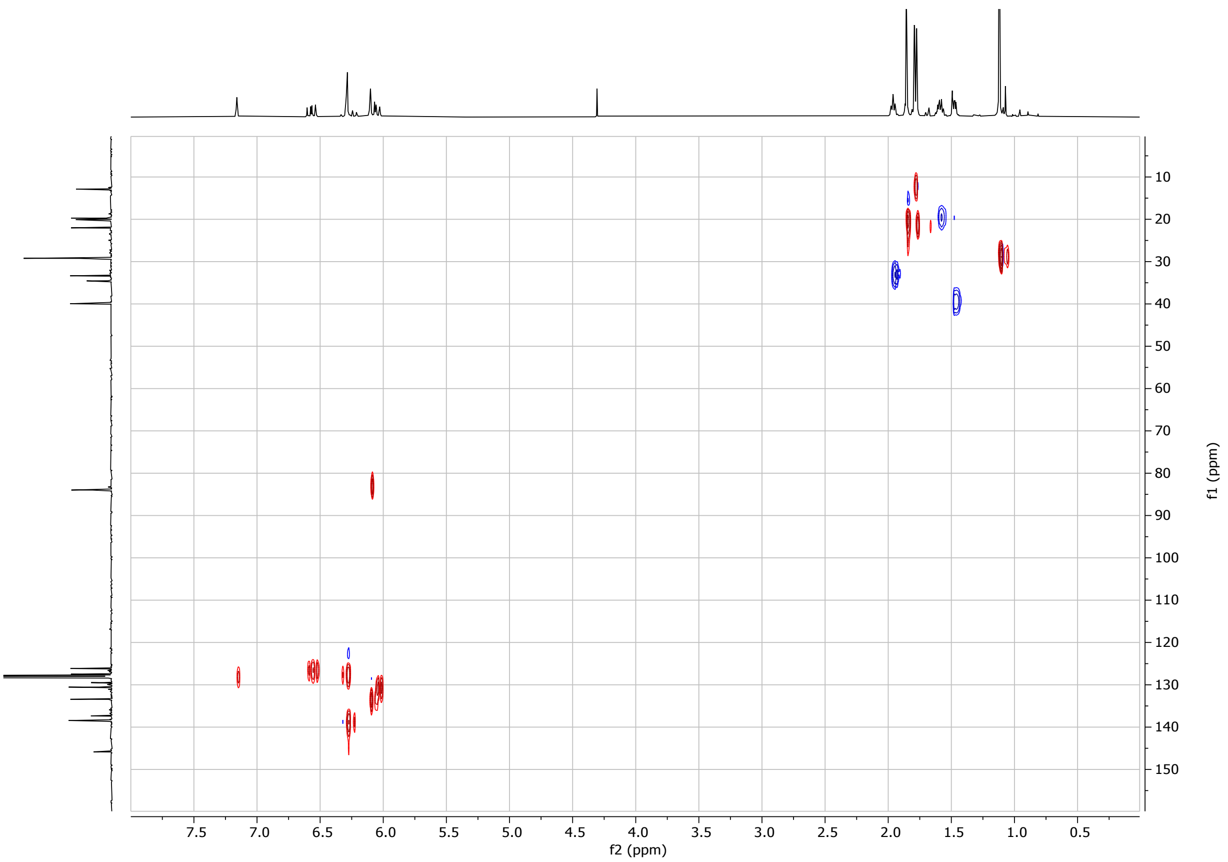

HMBC (C_6D_6)

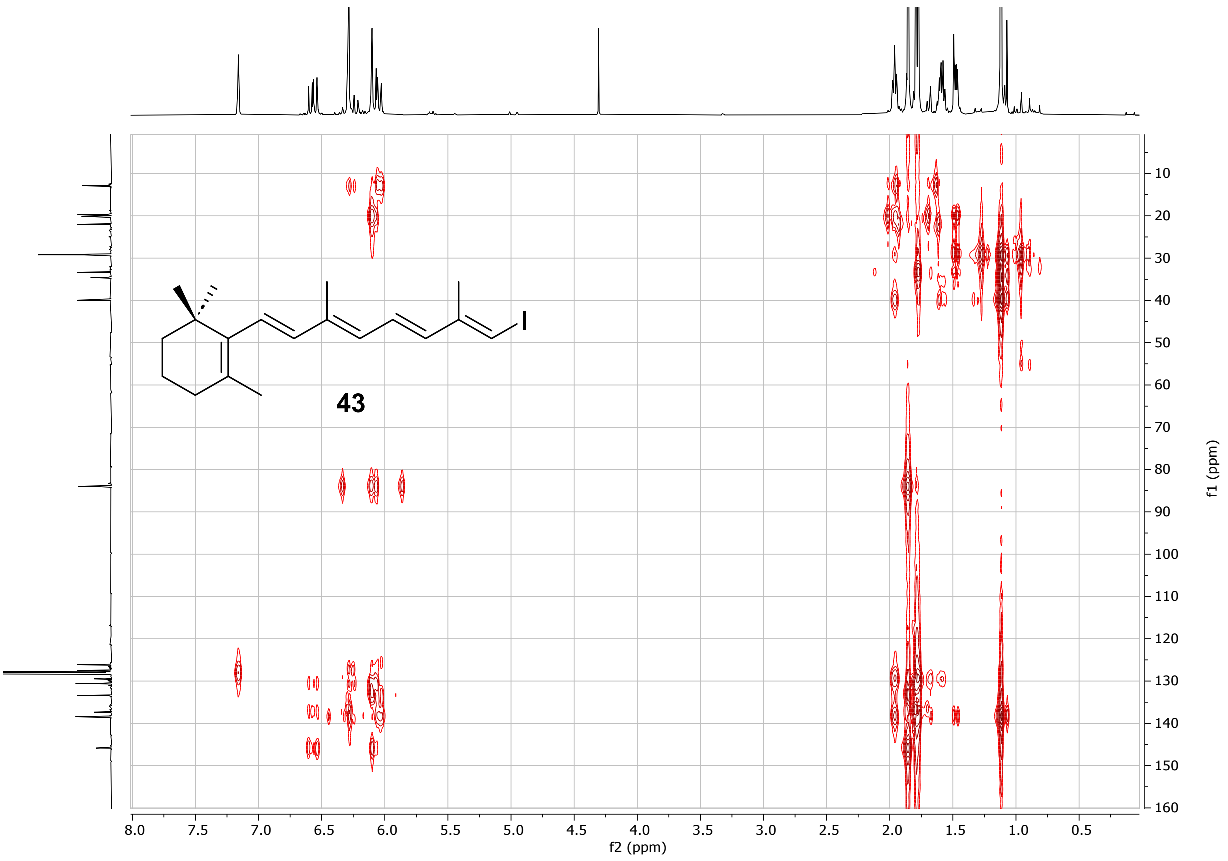
43
I
f2 (ppm)
f1 (ppm)
8.0 7.5 7.0 6.5 6.0 5.5 5.0 4.5 4.0 3.5 3.0 2.5 2.0 1.5 1.0 0.5
10 20 30 40 50 60 70 80 90 100 110 120 130 140 150 160

^{1}H-NMR (400.16 MHz, C_6D_6)

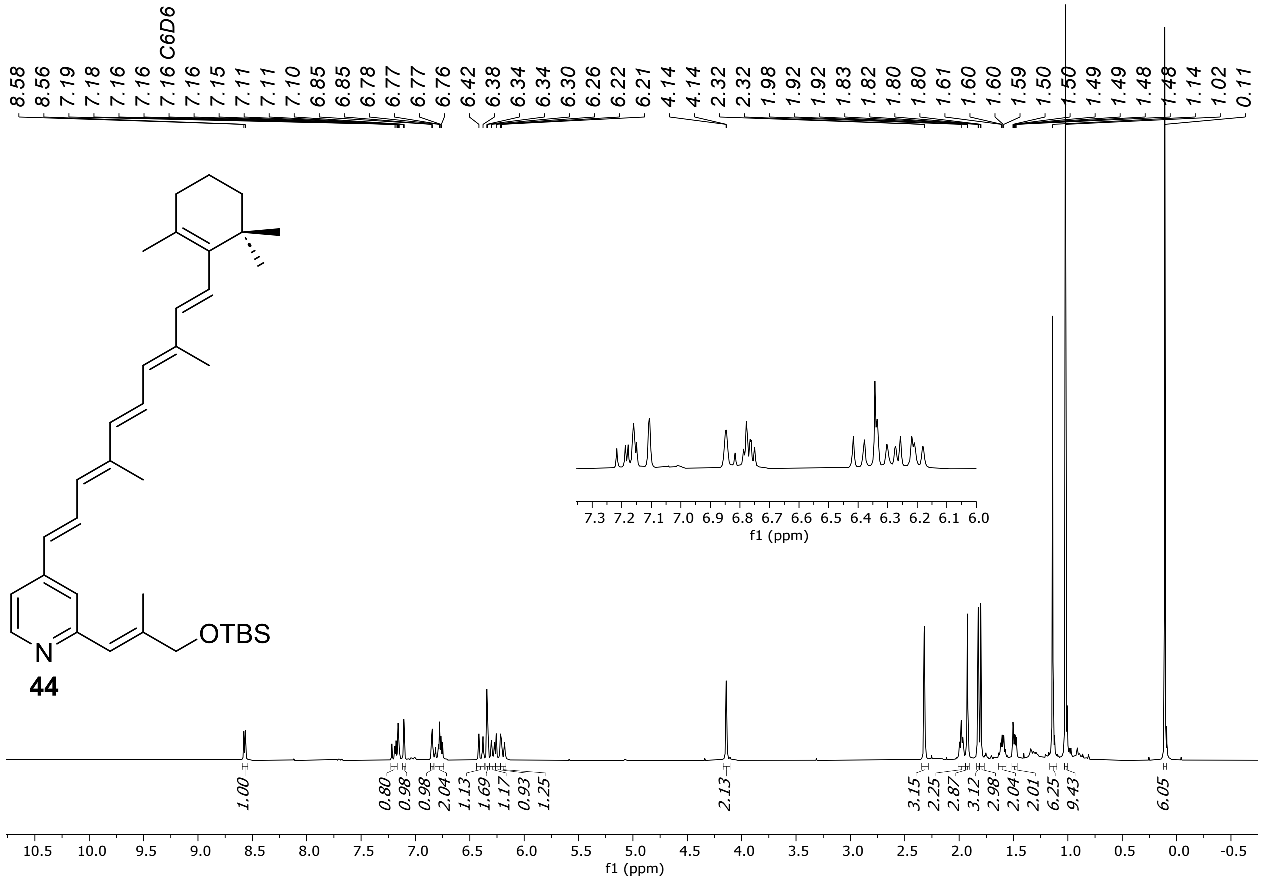

$^{13}C\{^{1}H\}$-NMR (100.63 MHz, C_6D_6)

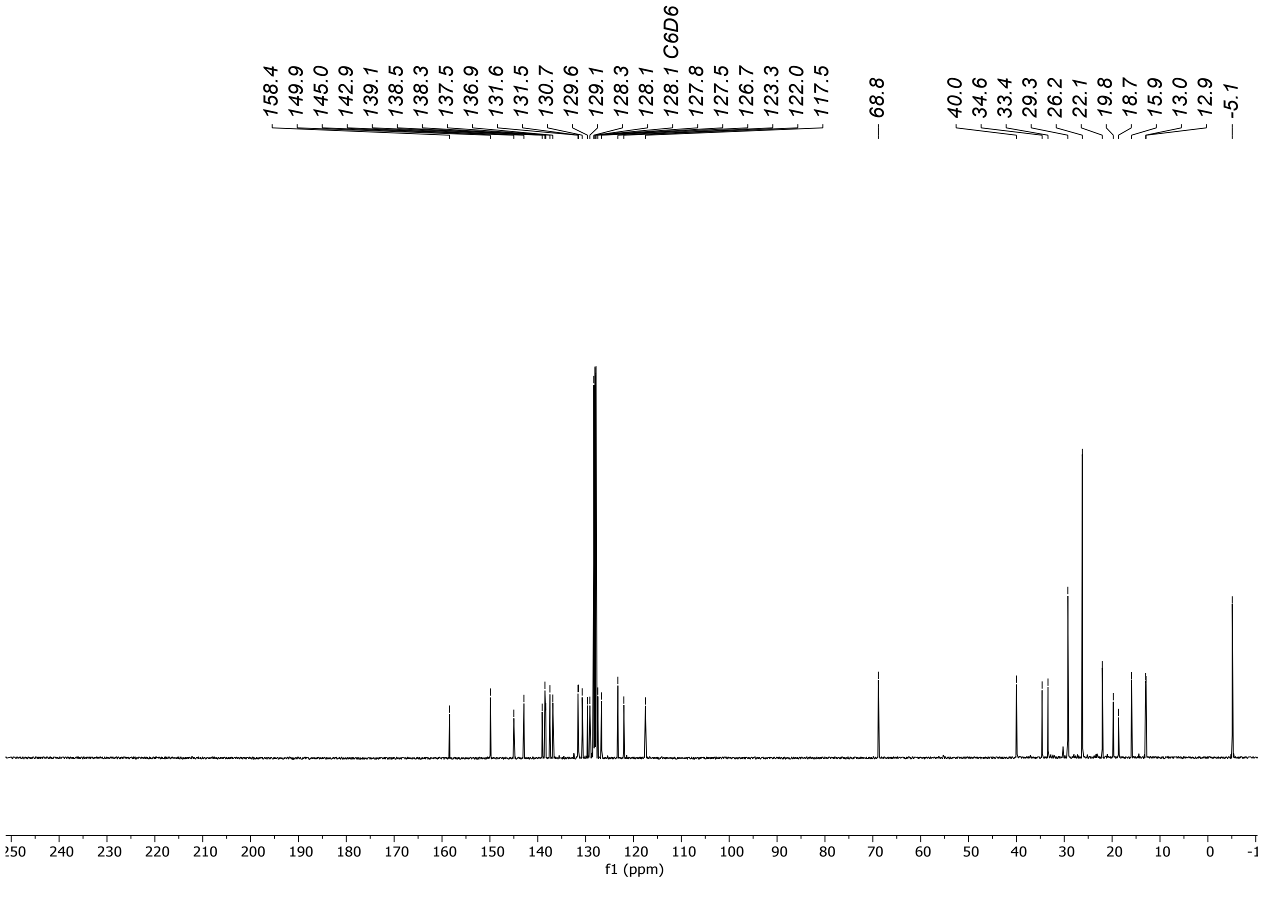

COSY (C_6D_6)

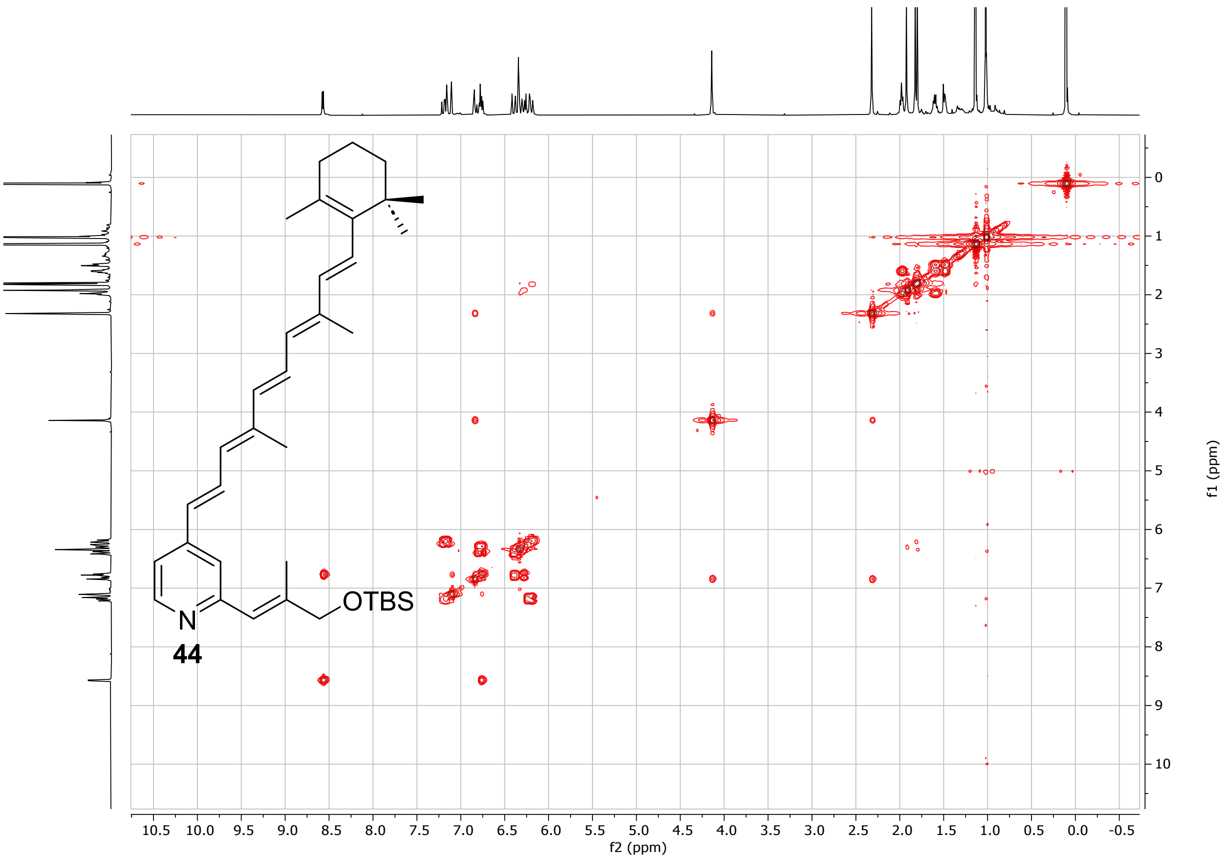

HSQC (C_6D_6)

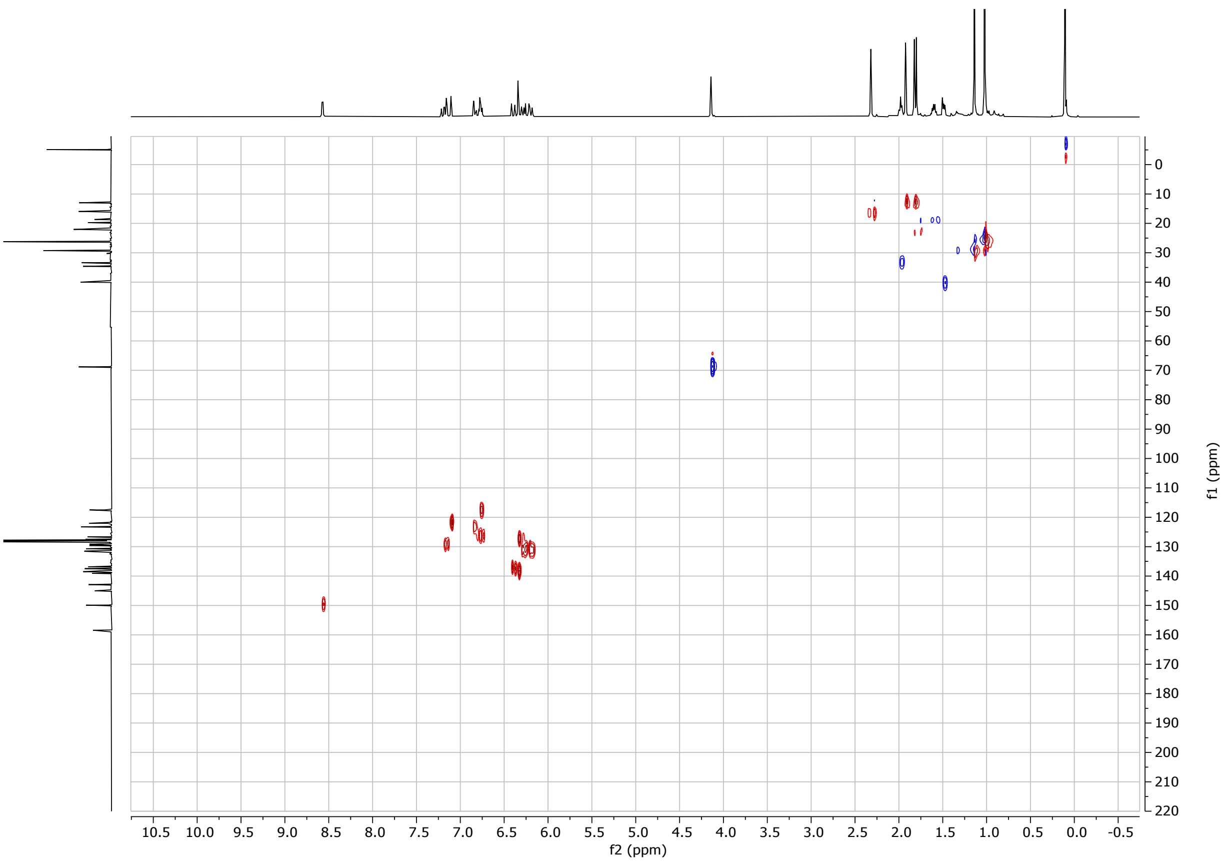

HMBC (C_6D_6)

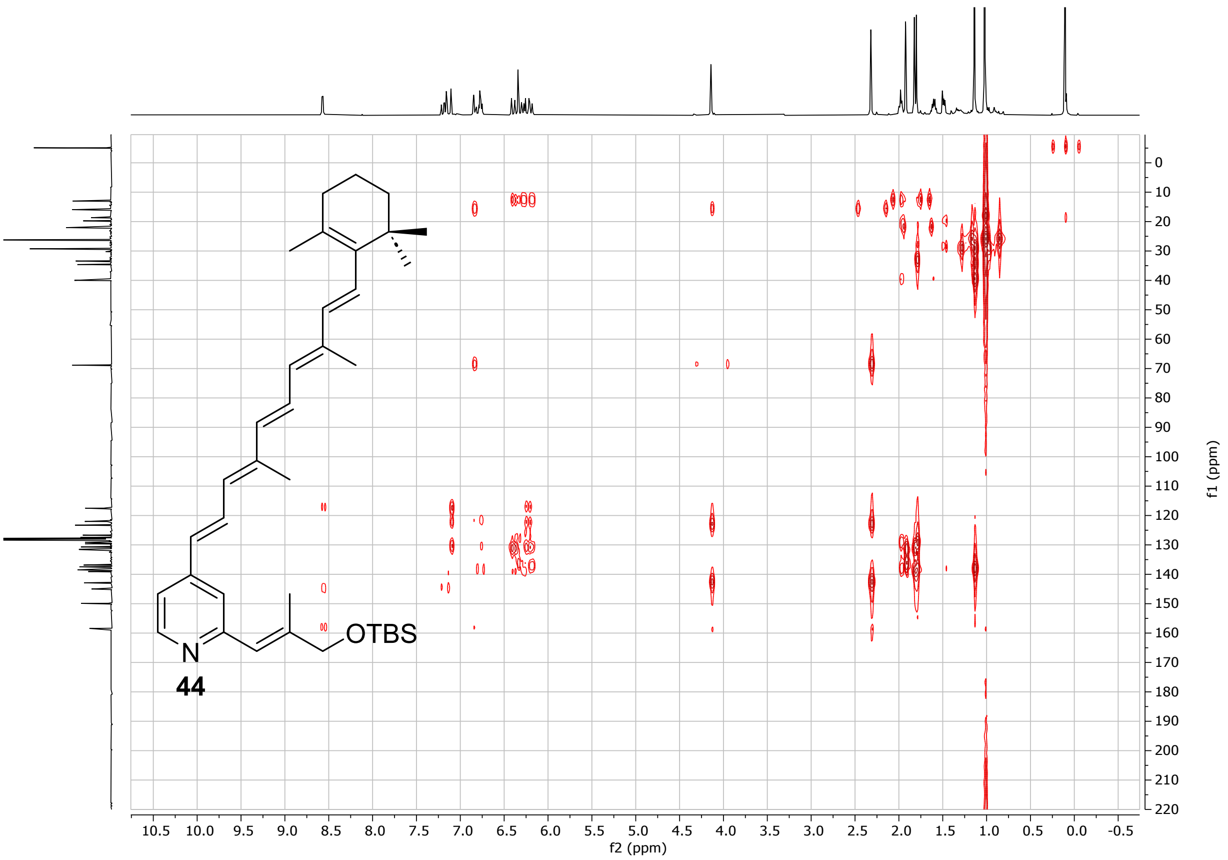

^{1}H-NMR (400.16 MHz, C_6D_6)

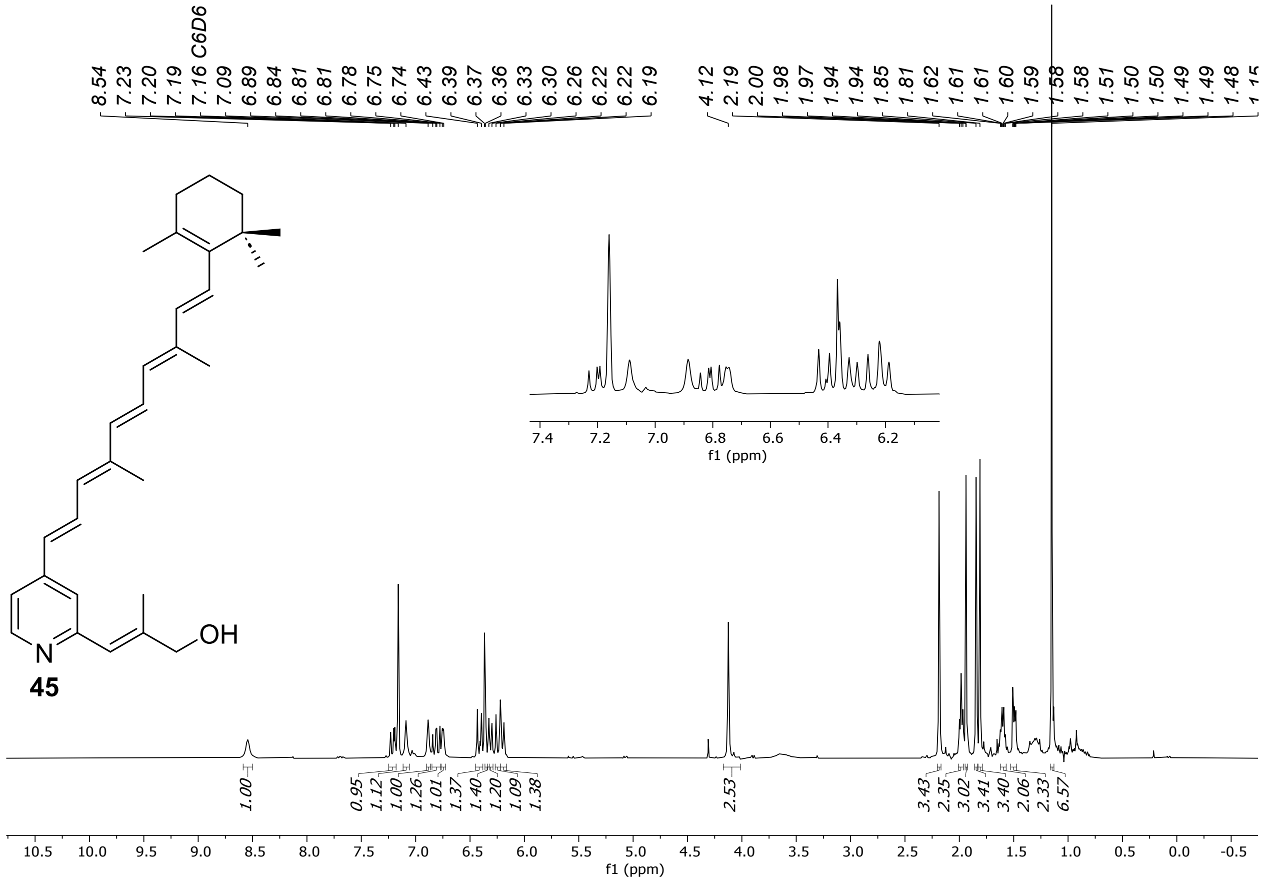

$^{13}C\{^{1}H\}$-NMR (100.63 MHz, C_6D_6)

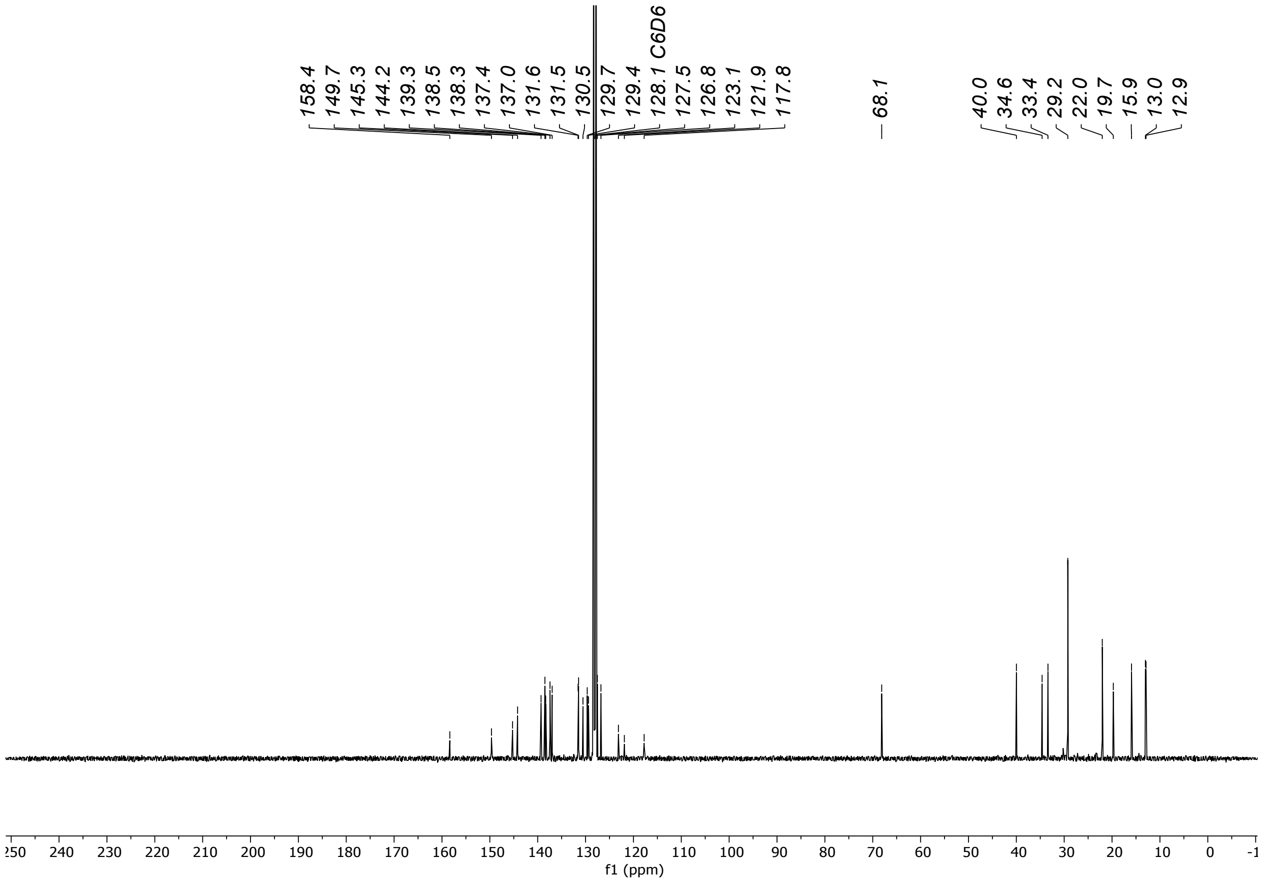

COSY (C_6D_6)

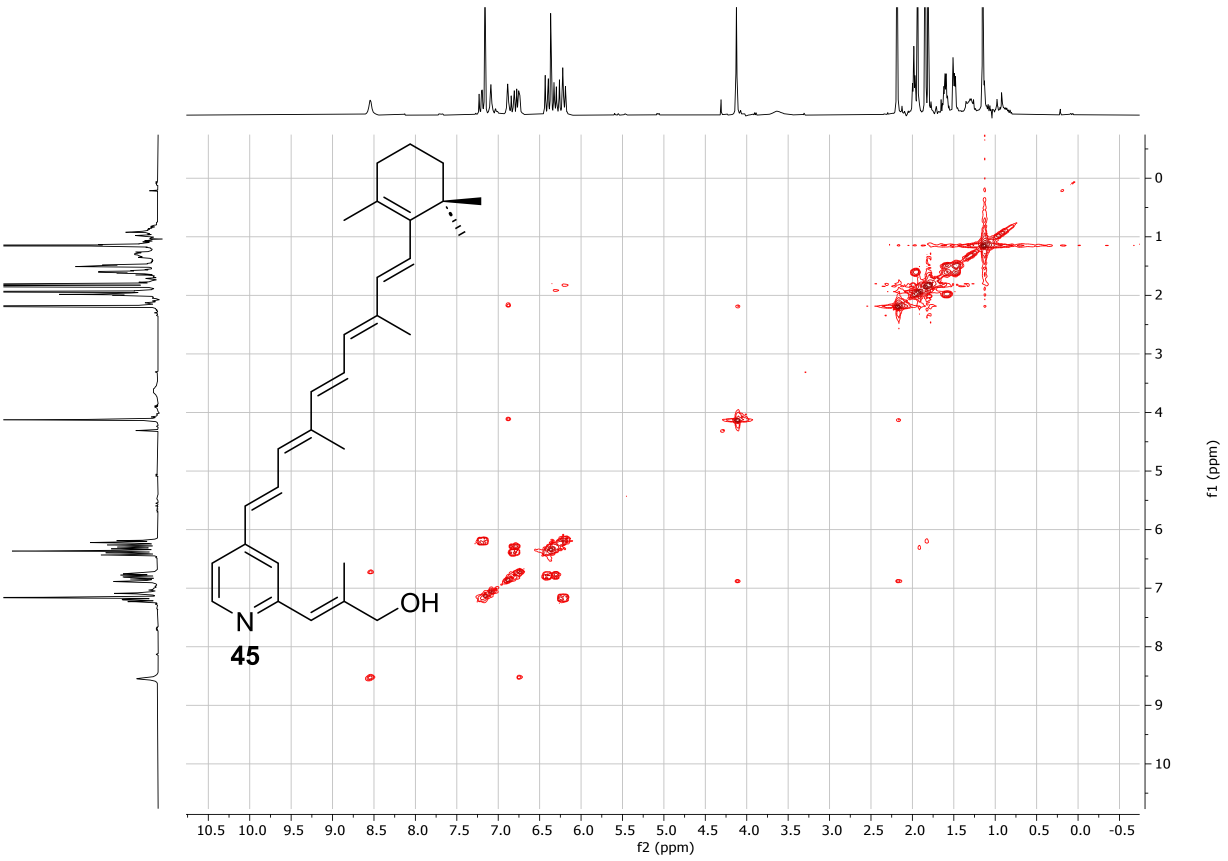

HSQC (C_6D_6)

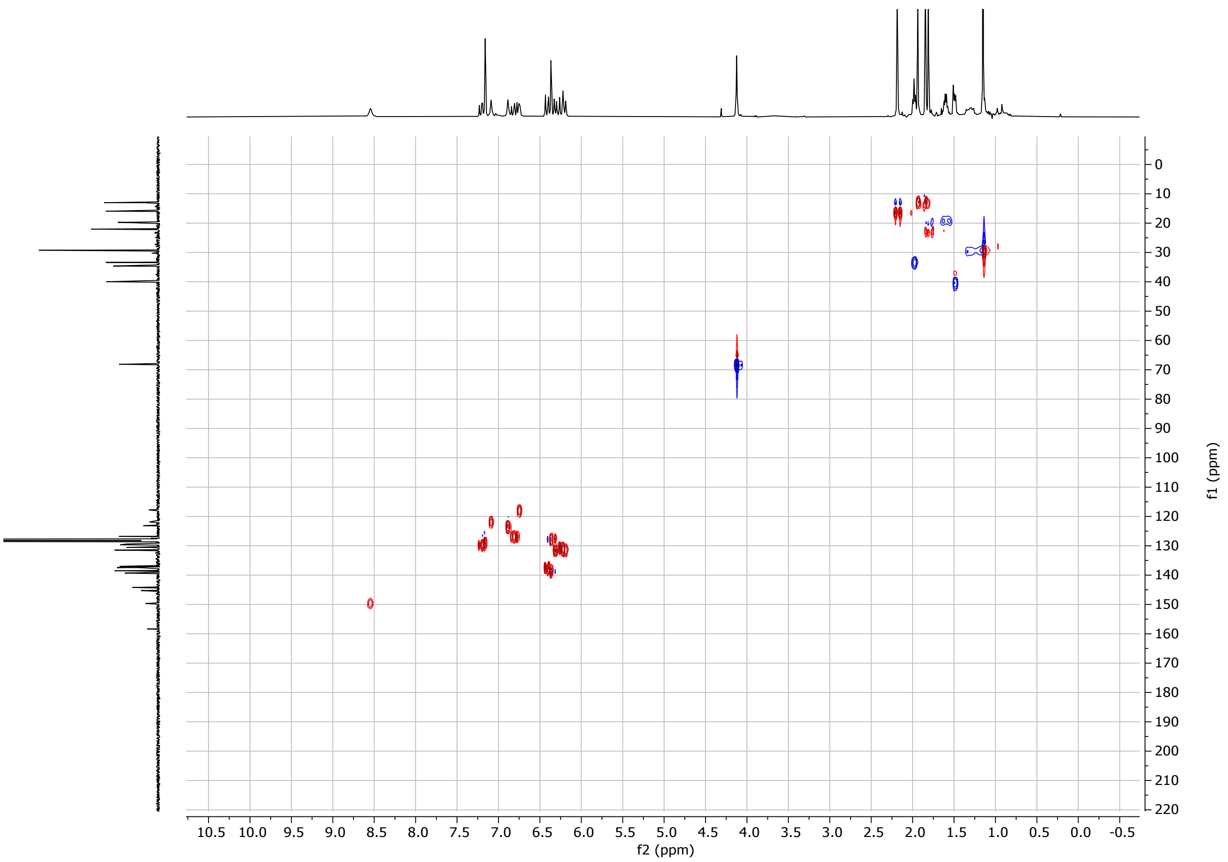

HMBC (C_6D_6)

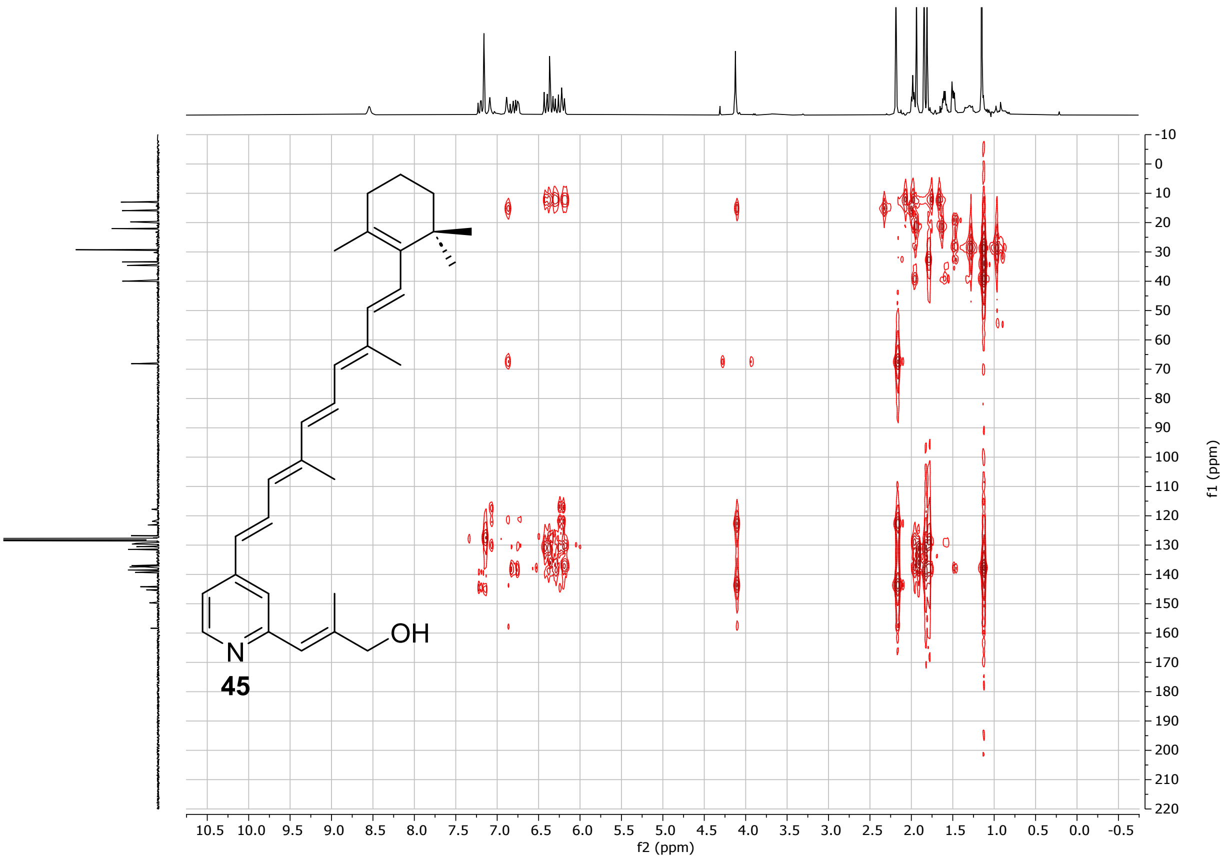

^{1}H-NMR (400.16 MHz, C_6D_6)

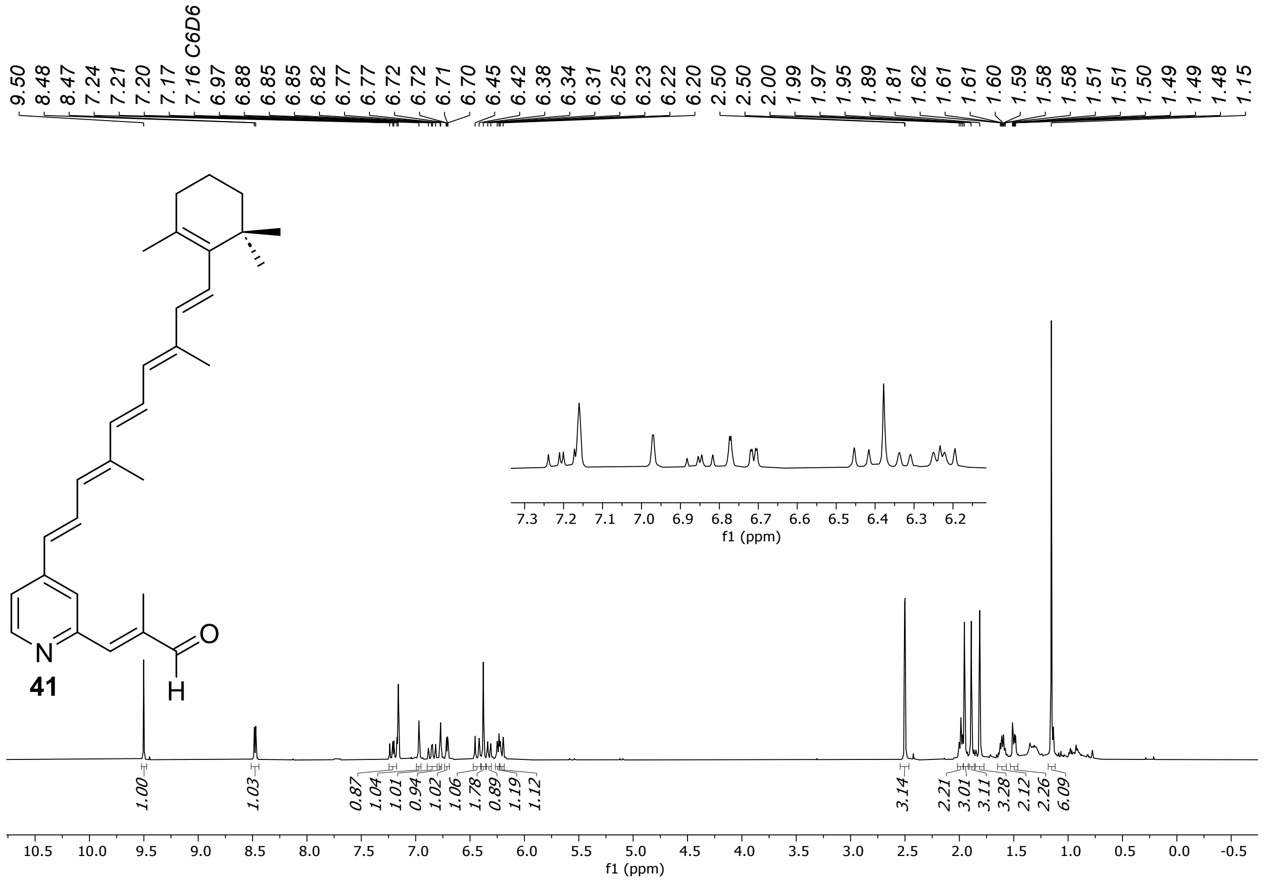

^{13}C{^{1}H}-NMR (100.63 MHz, C_6D_6)

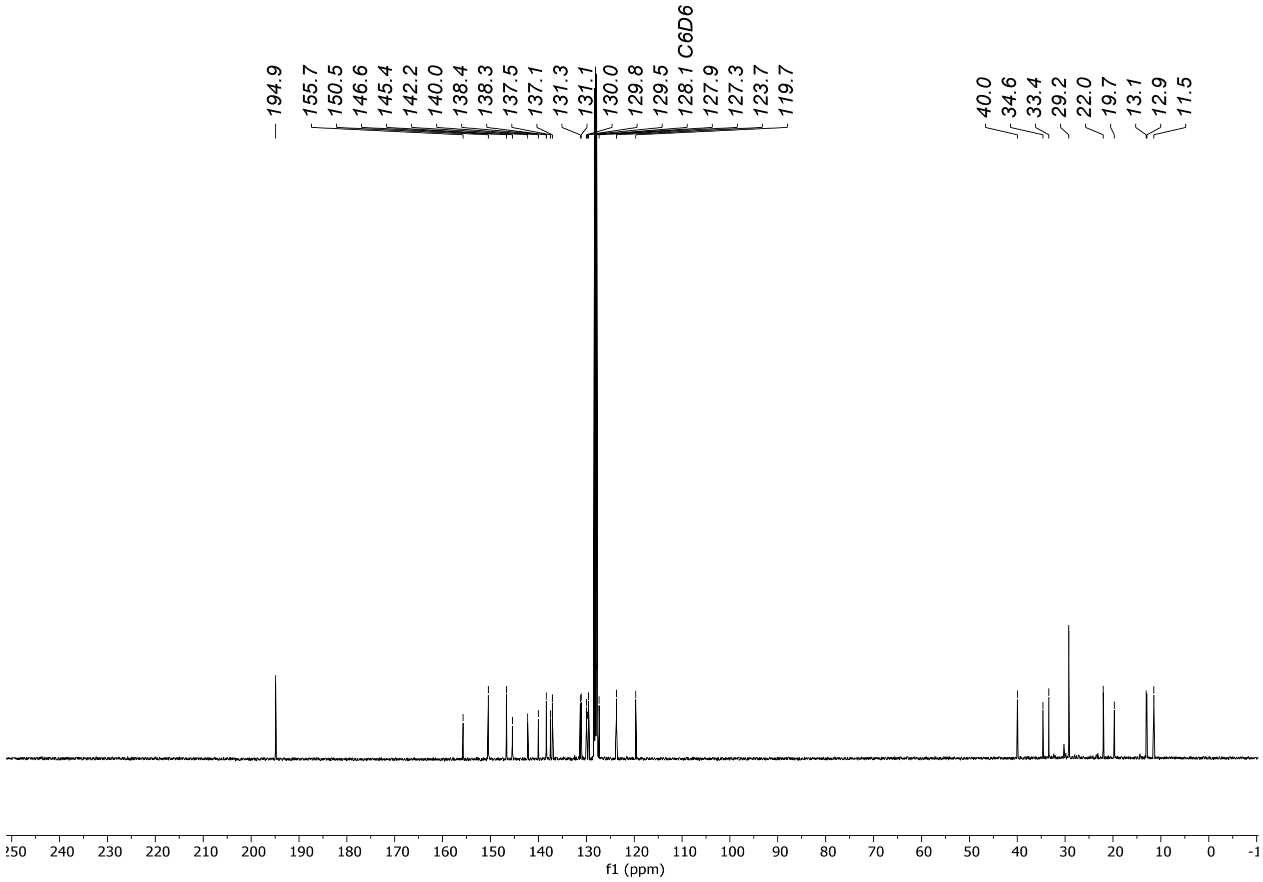

COSY (C_6D_6)

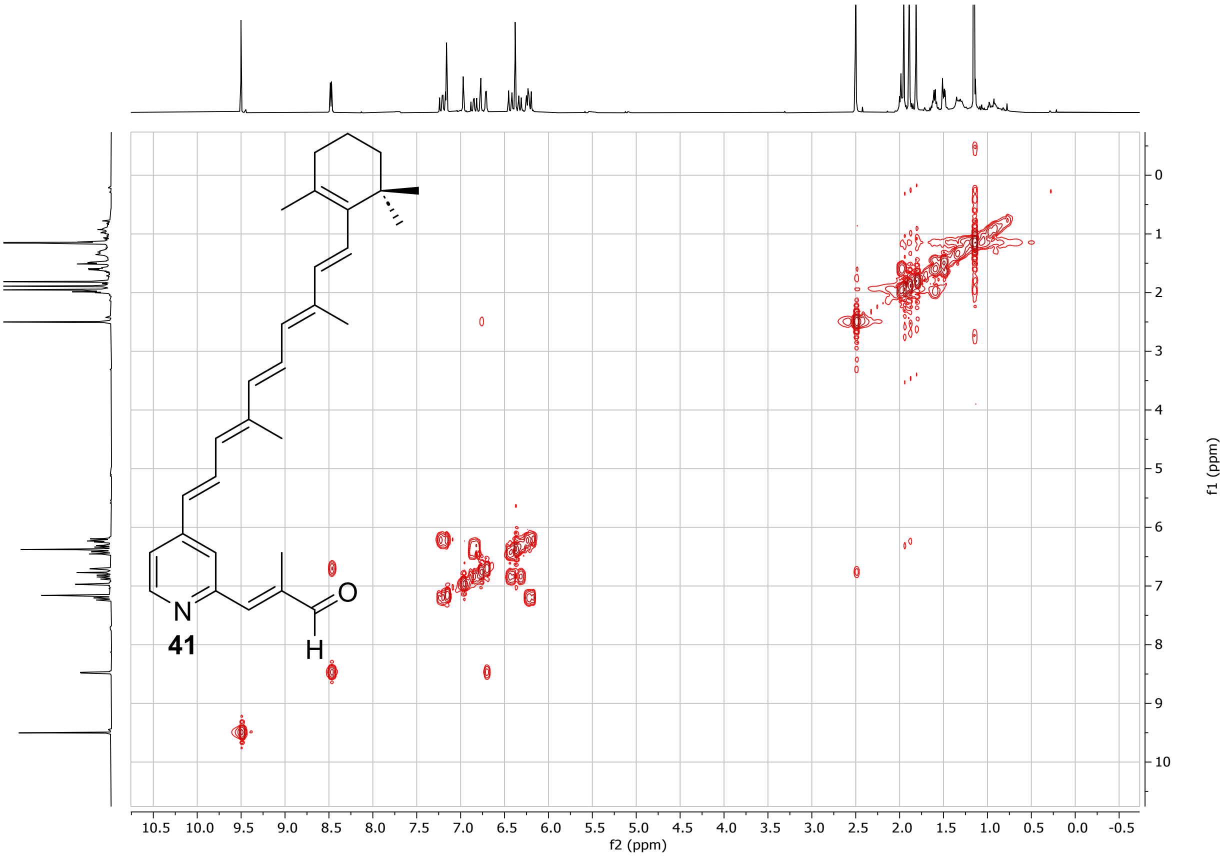

HSQC (C_6D_6)

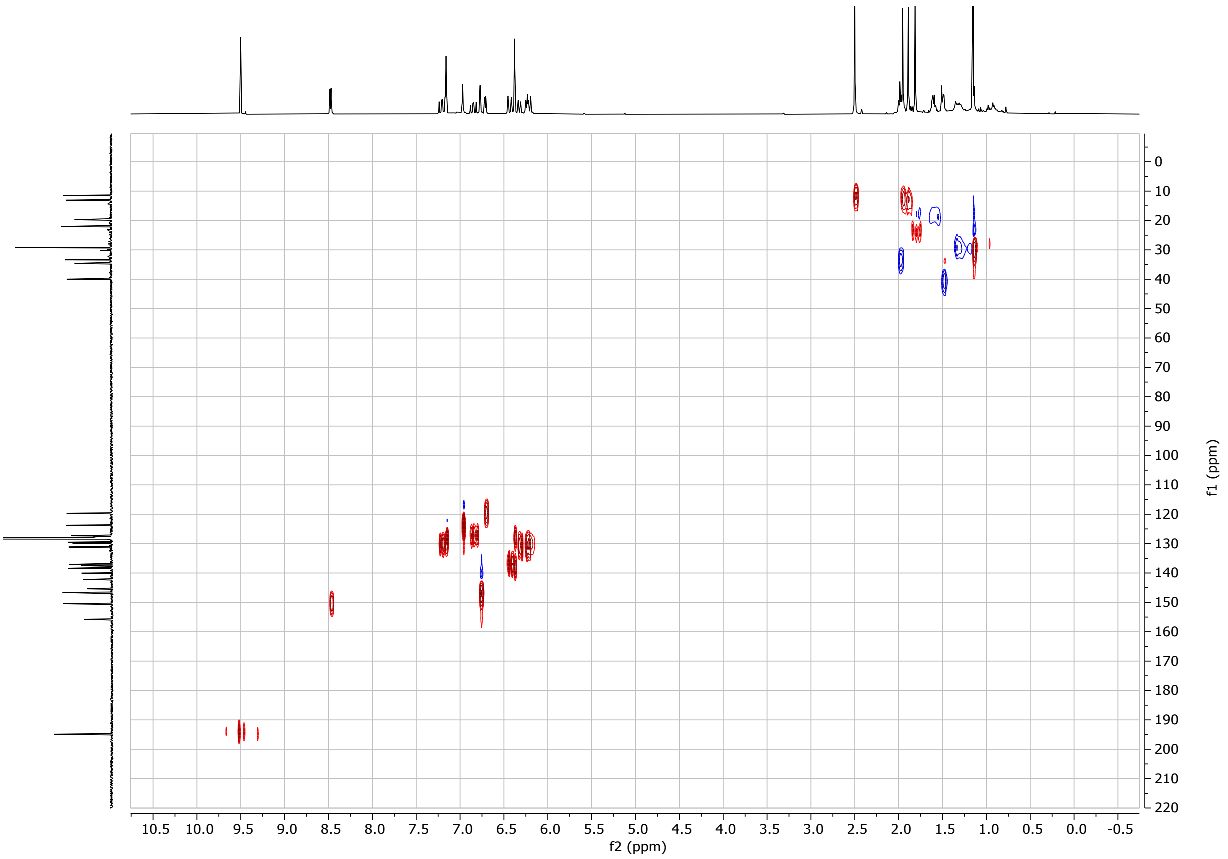

HMBC (C_6D_6)

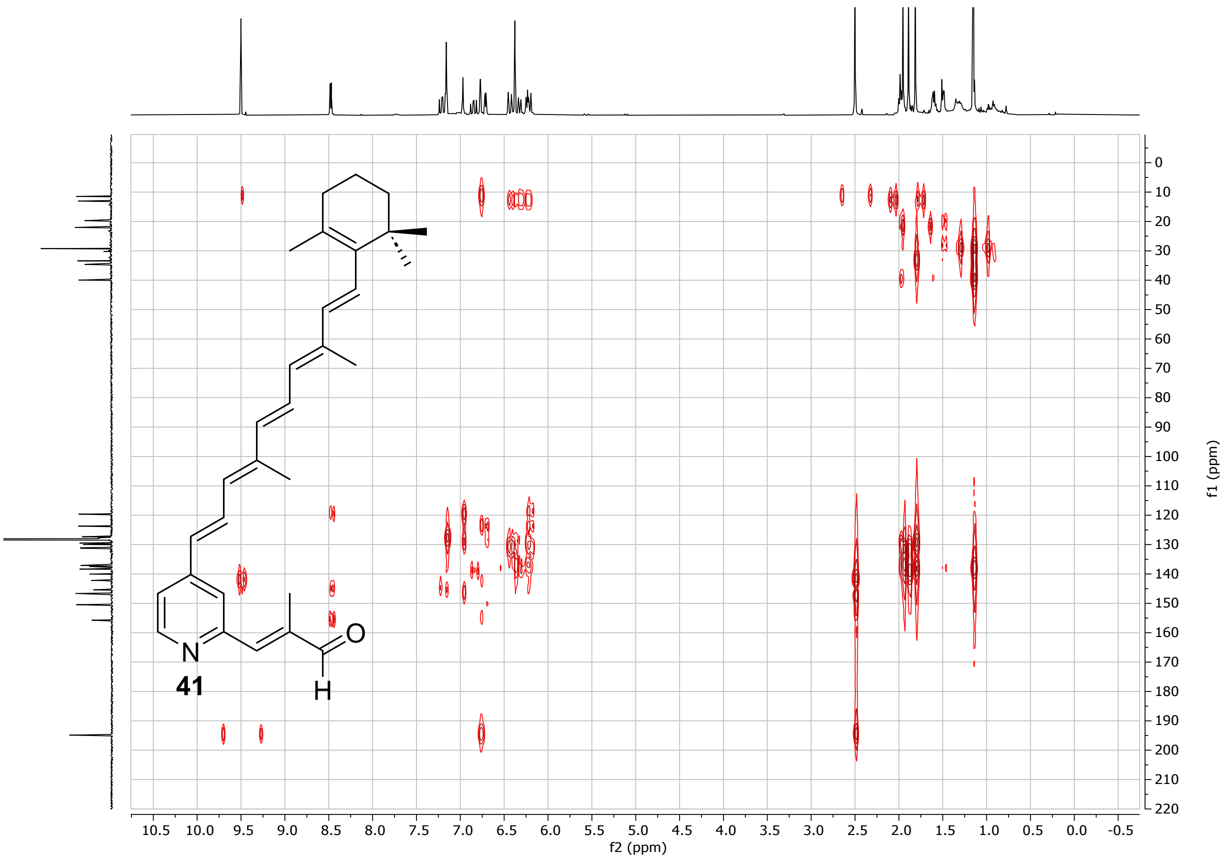

^{1}H-NMR (400.16 MHz, C_6D_6)

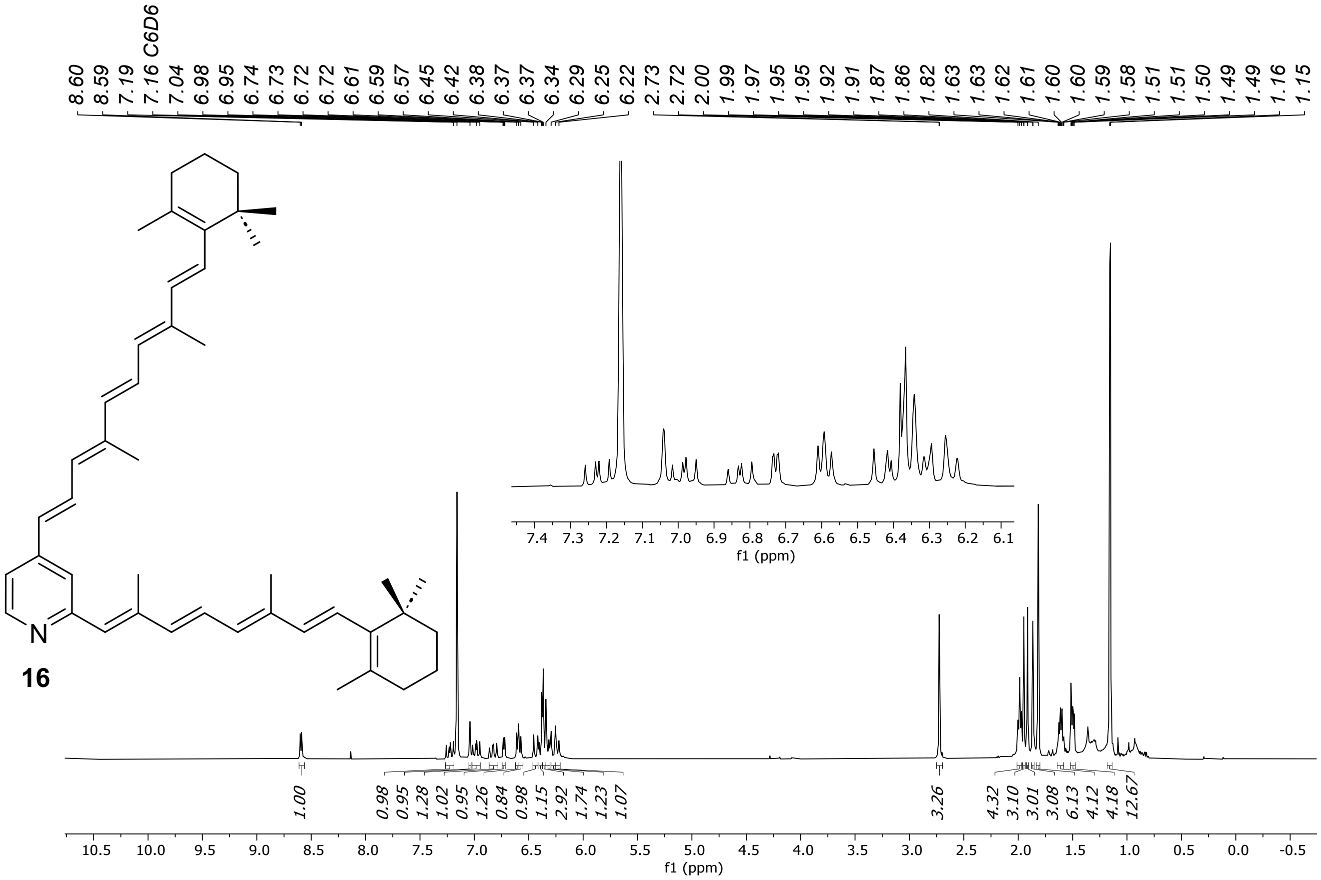

16

$^{13}C\{^{1}H\}$-NMR (100.63 MHz, C_6D_6)

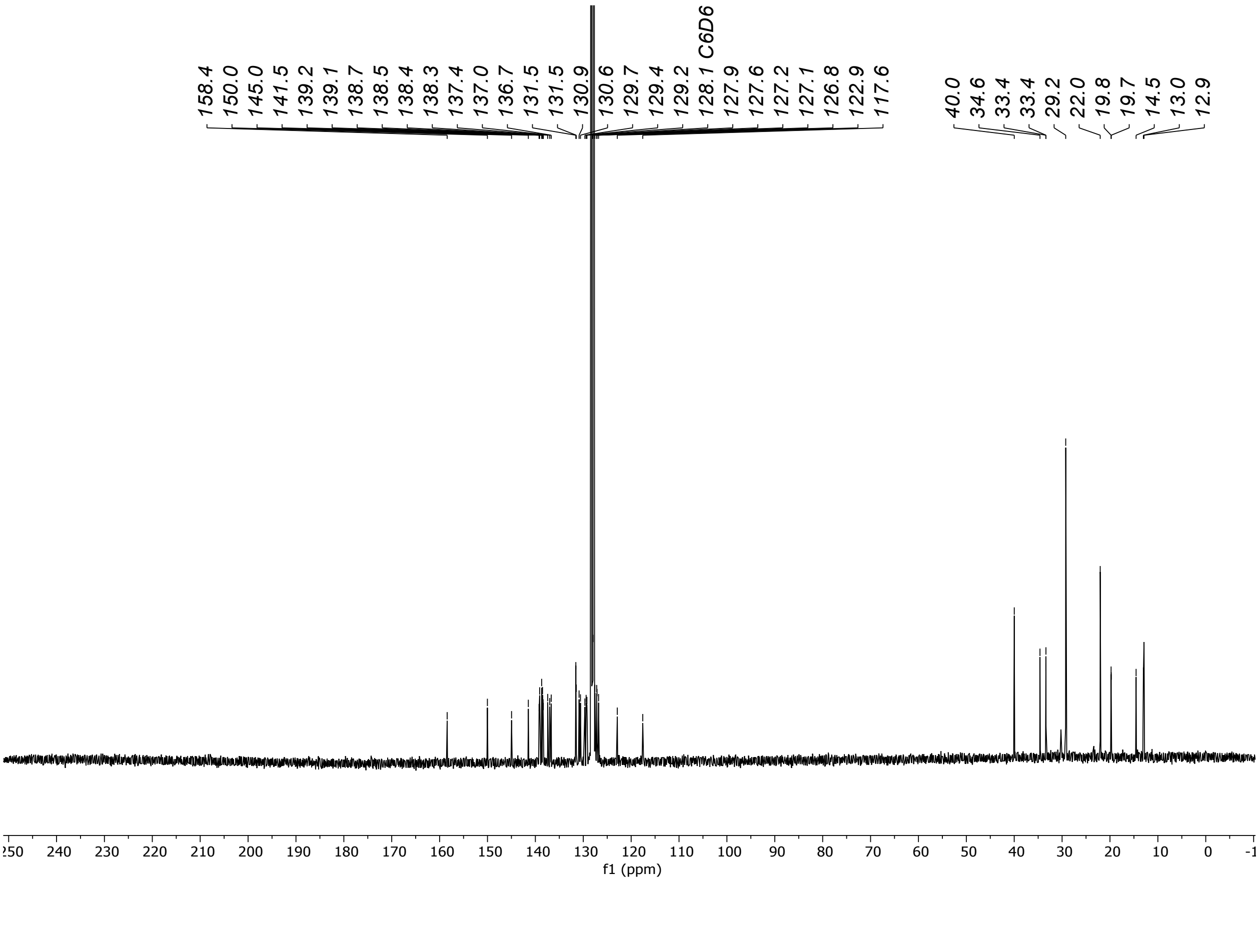

COSY (C_6D_6)

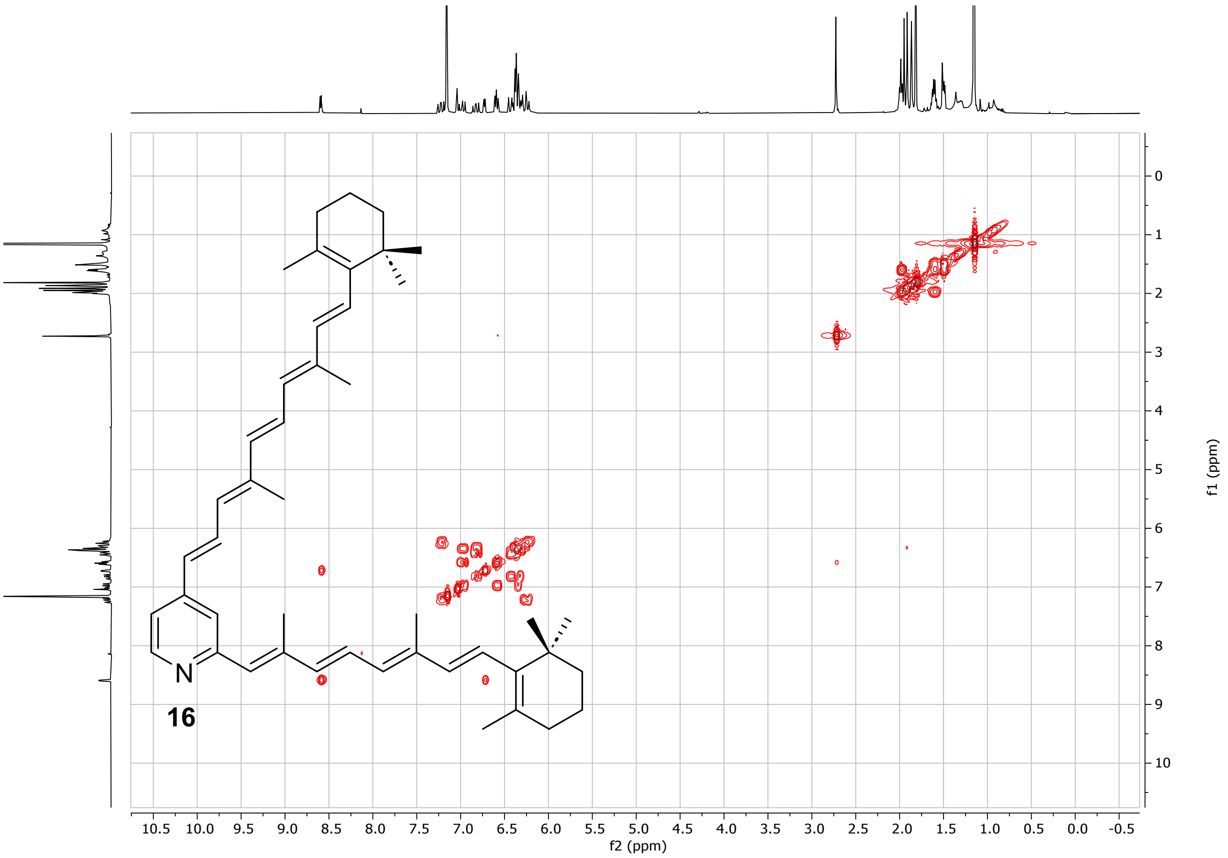

HSQC (C_6D_6)

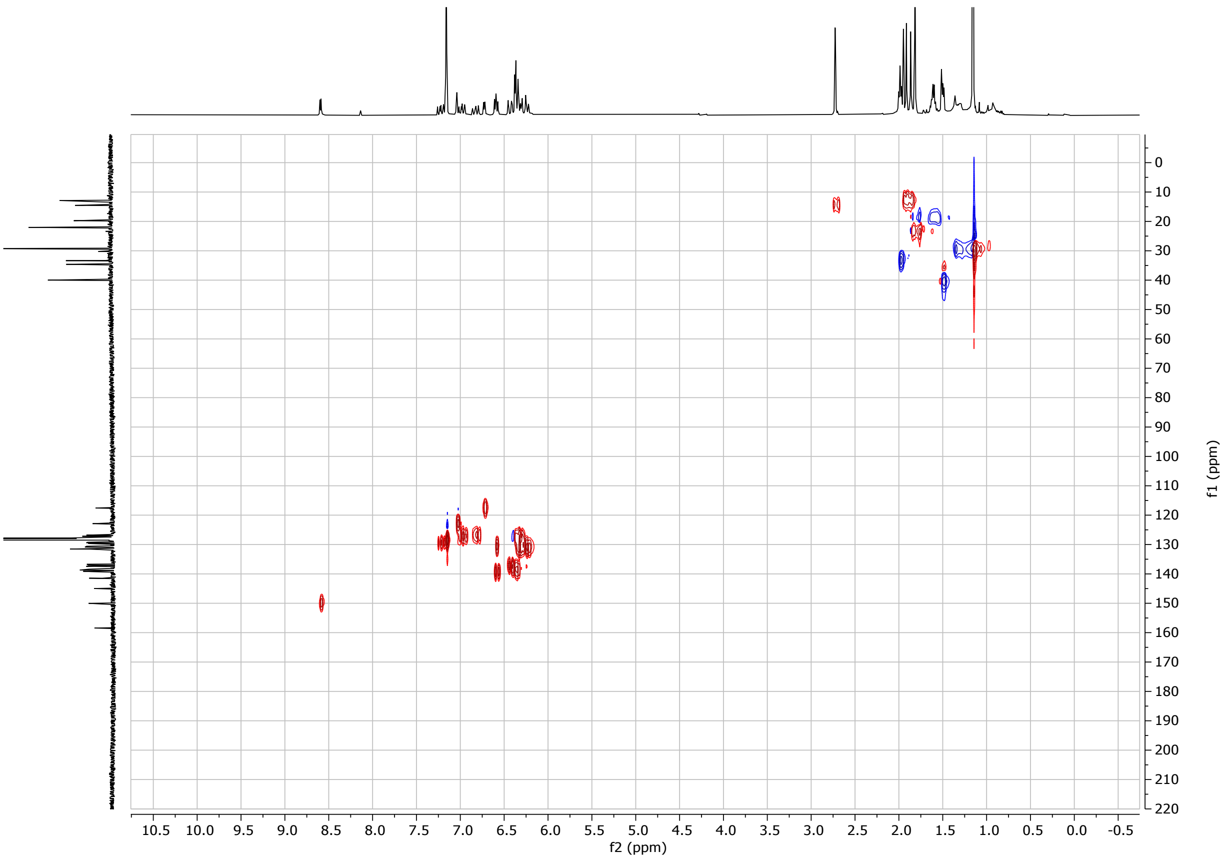

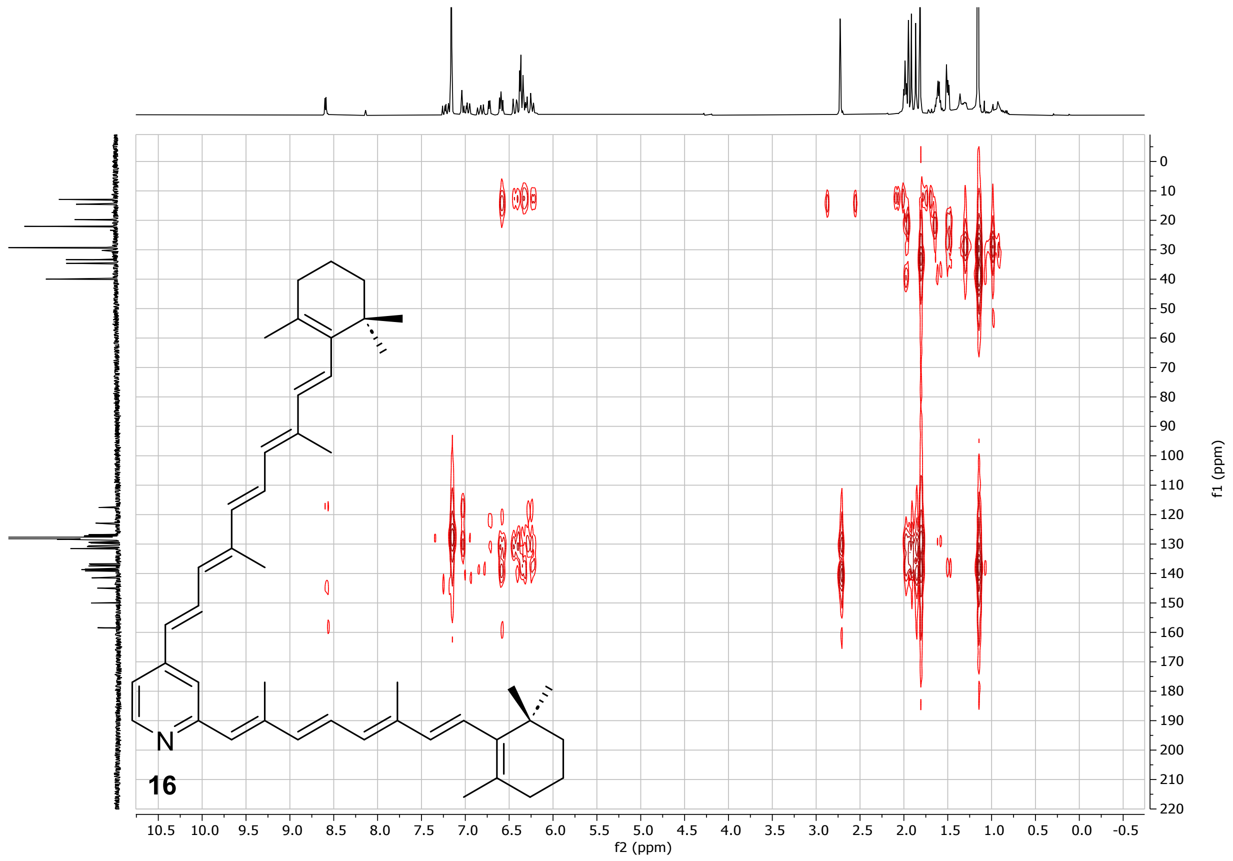
N
16
f2 (ppm)
f1 (ppm)

^{1}H-NMR (400.16 MHz, CD_3OD)

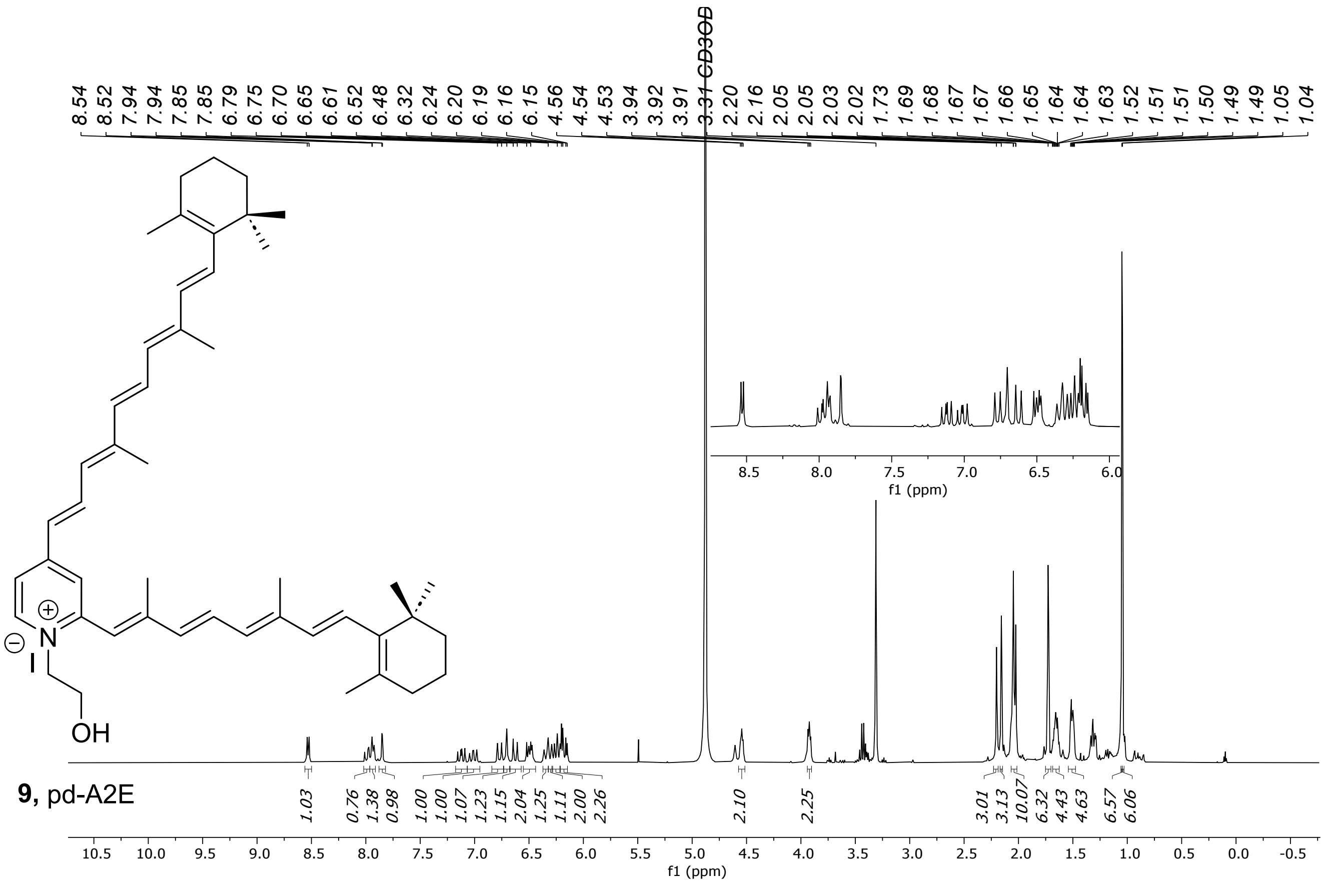

9, pd-A2E

$^{13}C\{^{1}H\}$-NMR (100.63 MHz, CD_3OD)

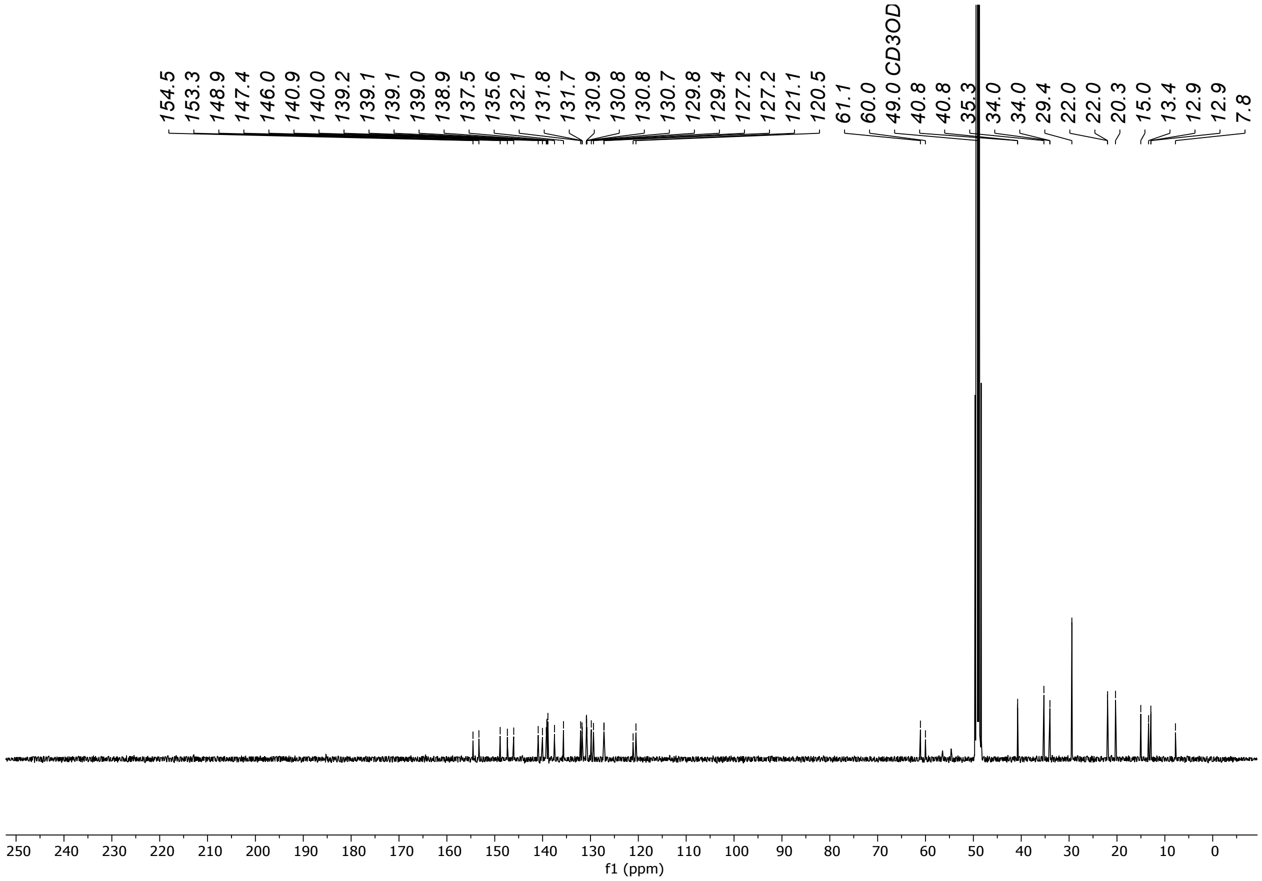

COSY (CD_3OD)

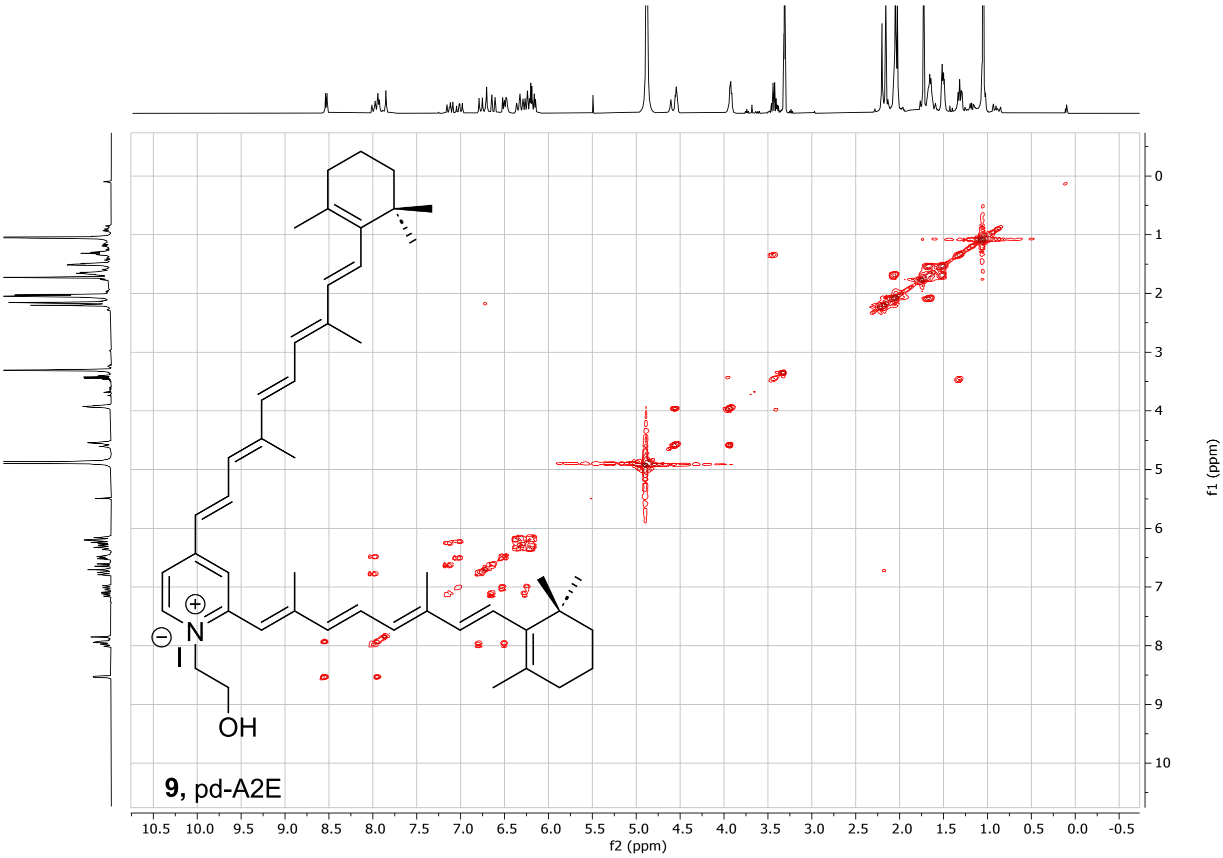

HSQC (CD_3OD)

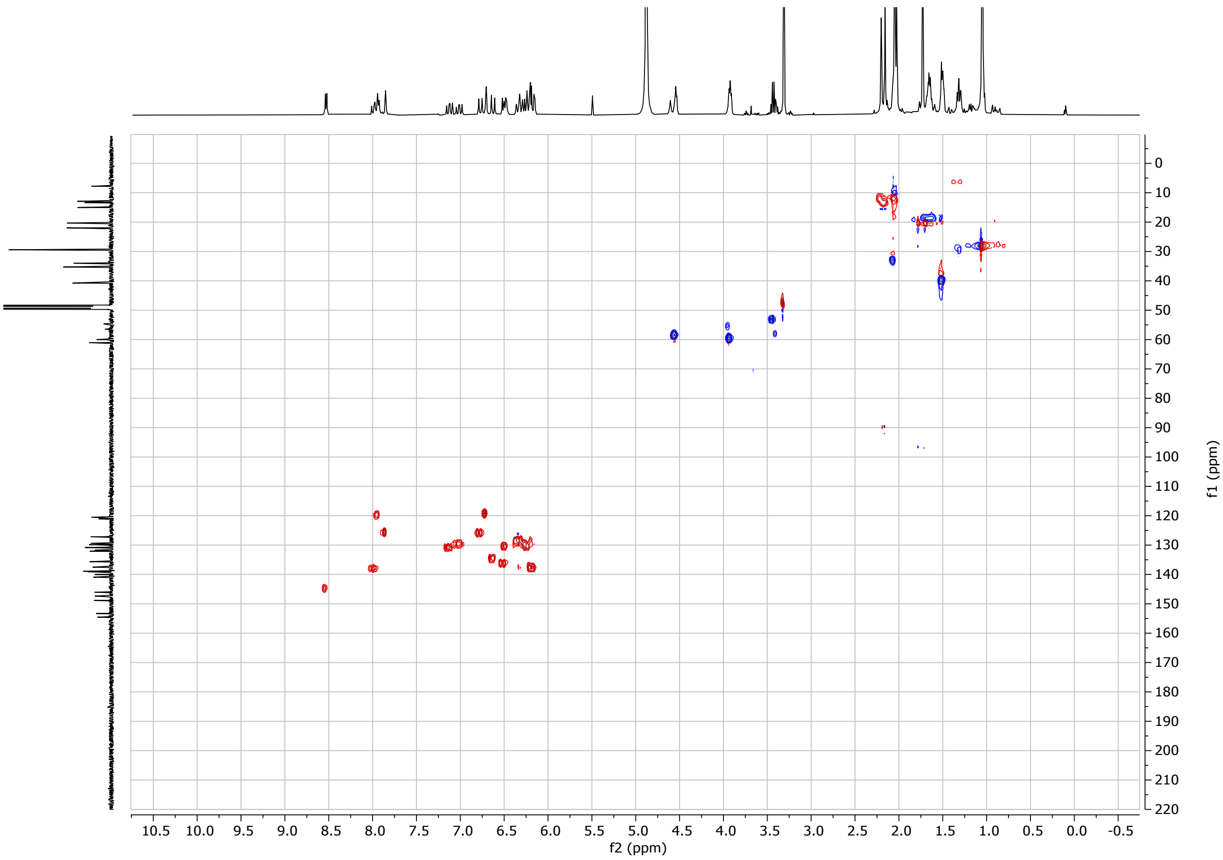

HMBC (CD_3OD)

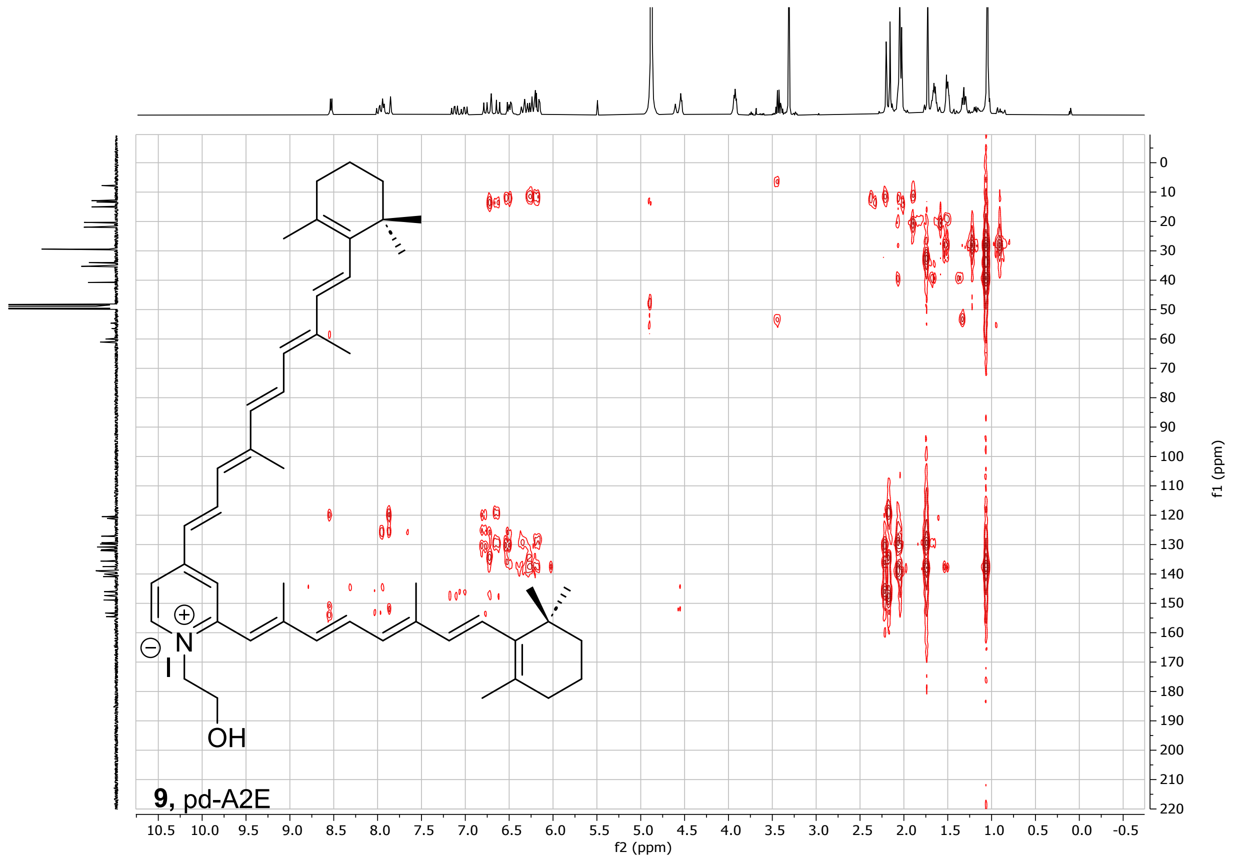

NOE-1D (400.16 MHz, freq. 7.12 ppm, CD_3OD)

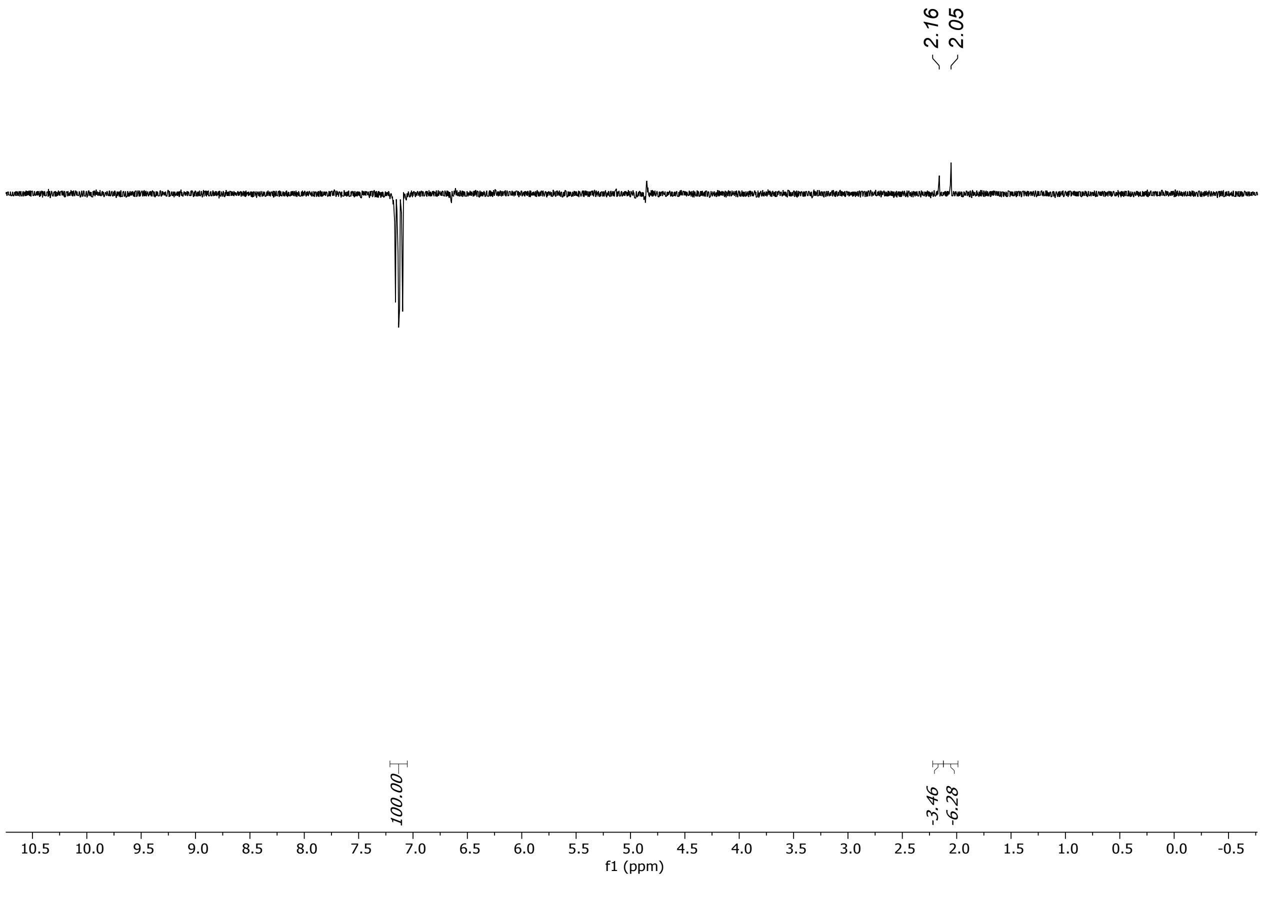

NOE-1D (400.16 MHz, freq. 7.01 ppm, CD_3OD)

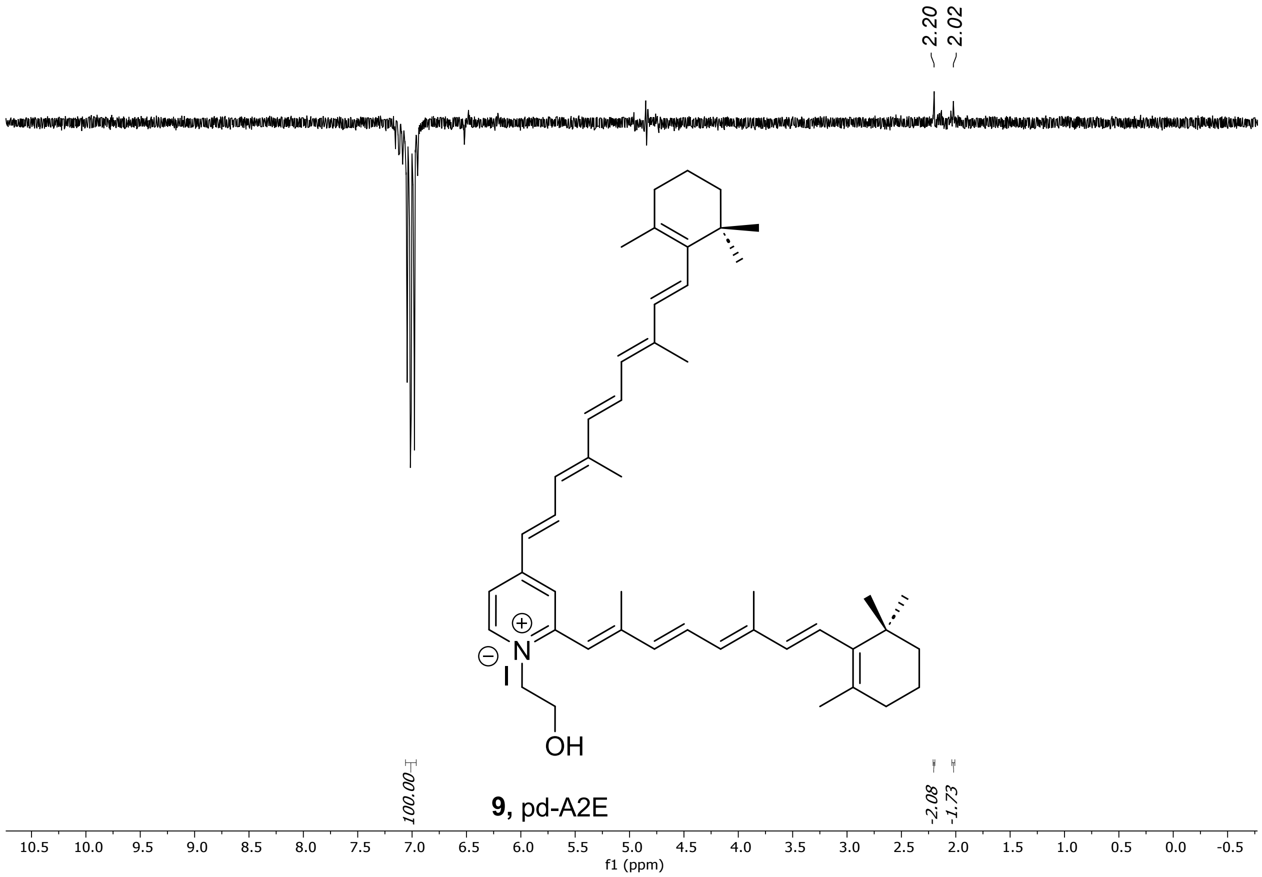

NOE-1D (400.16 MHz, freq. 2.16 ppm, CD_3OD)

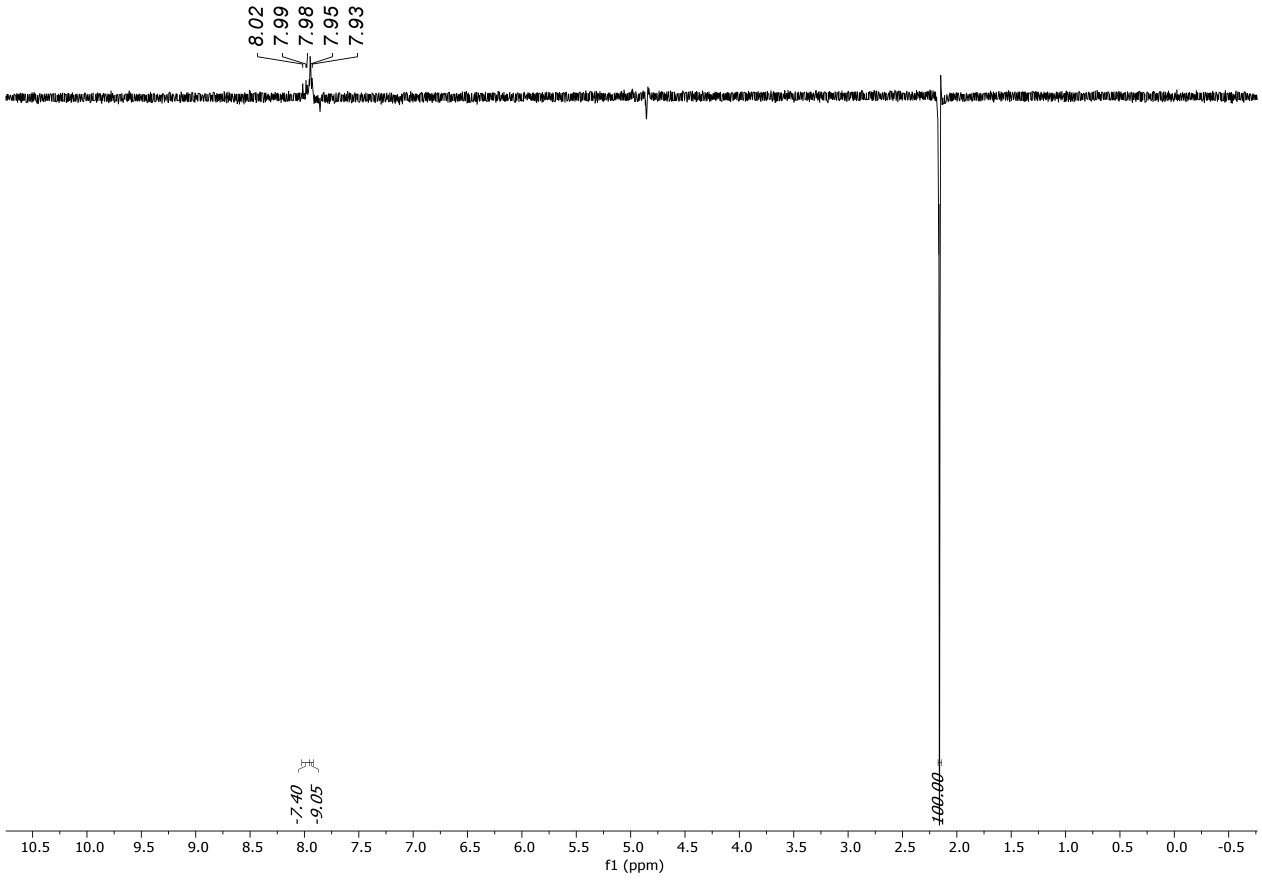

Supplement: Supplementary file 1 [file jo6c00763_si_001.pdf]
